# Engineering a Cysteine-Deficient Functional Candida albicans Cdr1 Molecule Reveals a Conserved Region at the Cytosolic Apex of ABCG Transporters Important for Correct Folding and Trafficking of Cdr1

**DOI:** 10.1128/mSphere.01318-20

**Published:** 2021-02-10

**Authors:** Golnoush Madani, Erwin Lamping, Richard D. Cannon

**Affiliations:** a Sir John Walsh Research Institute, Faculty of Dentistry, University of Otago, Dunedin, New Zealand; University of Georgia

**Keywords:** PDR transporters, *Candida albicans* Cdr1, cysteine-less Cdr1, NPAE motif, cysteine cross-linking, multidrug resistance, *Saccharomyces cerevisiae* hyperexpression

## Abstract

Pleiotropic drug resistance (PDR) ATP-binding cassette (ABC) transporters of the ABCG family are eukaryotic membrane proteins that pump an array of compounds across organelle and cell membranes. Overexpression of the archetype fungal PDR transporter Cdr1 is a major cause of azole antifungal drug resistance in Candida albicans, a significant fungal pathogen that can cause life-threatening invasive infections in immunocompromised individuals. To date, no structure for any PDR transporter has been solved. The objective of this project was to investigate the role of the 23 Cdr1 cysteine residues in the stability, trafficking, and function of the protein when expressed in the eukaryotic model organism, Saccharomyces cerevisiae. The biochemical characterization of 18 partially cysteine-deficient Cdr1 variants revealed that the six conserved extracellular cysteines were critical for proper expression, localization, and function of Cdr1. They are predicted to form three covalent disulfide bonds that stabilize the large extracellular domains of fungal PDR transporters. Our investigations also revealed a novel nucleotide-binding domain motif, GX_2[3]_CPX_3_NPAD/E, at the peripheral cytosolic apex of ABCG transporters that possibly contributes to the unique ABCG transport cycle. With this knowledge, we engineered an “almost cysteine-less,” yet fully functional, Cdr1 variant, Cdr1P-CID, that had all but the six extracellular cysteines replaced with serine, alanine, or isoleucine (C1106I of the new motif). It is now possible to perform cysteine-cross-linking studies that will enable more detailed biochemical investigations of fungal PDR transporters and confirm any future structure(s) solved for this important protein family.

**IMPORTANCE** Overexpression of the fungal pleiotropic drug resistance (PDR) transporter Cdr1 is a major cause of antifungal drug resistance in Candida albicans, a significant fungal pathogen that can cause life-threatening invasive infections in immunocompromised individuals. To date, no structure for any PDR ABC transporter has been solved. Cdr1 contains 23 cysteines; 10 are cytosolic and 13 are predicted to be in the transmembrane or the extracellular domains. The objective of this project was to create, and biochemically characterize, *CDR1* mutants to reveal which cysteines are most important for Cdr1 stability, trafficking, and function. During this process we discovered a novel motif at the cytosolic apex of PDR transporters that ensures the structural and functional integrity of the ABCG transporter family. The creation of a functional Cys-deficient Cdr1 molecule opens new avenues for cysteine-cross-linking studies that will facilitate the detailed characterization of an important ABCG transporter family member.

## INTRODUCTION

Candida albicans is part of the oral microflora in 30% to 50% of healthy individuals (1). However, it can cause serious, life-threatening, invasive fungal infections in immunocompromised individuals (2–4). One of the predominant azole resistance mechanisms in C. albicans infections is overexpression of the plasma membrane multidrug efflux pump Cdr1 (5–8). Cdr1 is a type II ABC transporter of the ABCG family of ABC transporters (9) and one of seven full-size C. albicans pleiotropic drug resistance (PDR) transporters (10).

**FIG 1:**
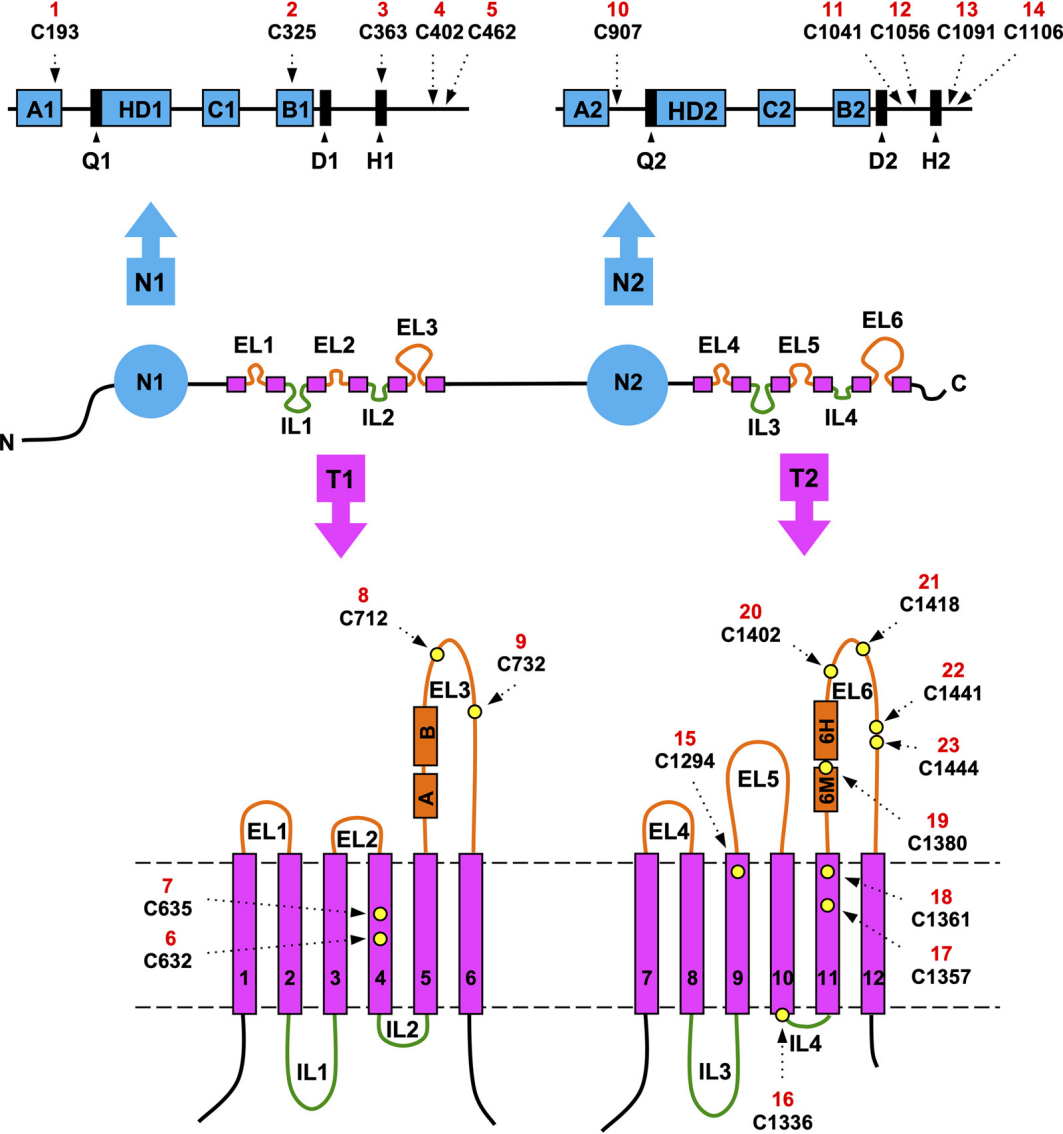
Graphical illustration of C. albicans Cdr1 indicating the location of the cysteine residues. The center panel indicates the presumed topology of Cdr1; N, N terminus; C, C terminus. The positions (dashed arrows) of the 10 cytosolic cysteines (i.e., cysteines 1 to 5 of the N-terminal NBD N1 and cysteines 10 to 14 of the C-terminal NBD N2) are shown above, and the positions of the 13 cysteines of the TMD regions (i.e., cysteines 6 to 9 in T1 and 15 to 23 in T2) are indicated as yellow circles underneath the center panel. Each cysteine was given a unique identifier from N to C terminus of Cdr1 (red numbers 1 to 23). The Walker A1, Walker A2, Walker B1, and Walker B2 motifs, the helical domains HD1 and HD2, and the ABC1 (C1) and ABC2 (C2) signature motifs are shown as blue boxes, and the Q-loop, D-loop, and H-loop regions of NBD1 (Q1, D1, H1) and NBD2 (Q2, D2, H2) are shown as black boxes (top). The TMD topology of Cdr1 at the bottom is drawn approximately to scale. It shows individual TMSs (magenta rectangles) numbered from 1 to 12 and the PDR motifs (11) (orange boxes; PDRA [A] and PDRB [B] and EL6 motif [6M] and EL6 helix [6H]) near the N termini of EL3 and EL6, respectively. Each pair of PDR motifs is separated by a conserved three-residue proline-kink. Plasma membrane boundaries are indicated with dashed gray lines.

C. albicans Cdr1 has 1,501 amino acids, a molecular weight of 170 kDa, and an inverted nucleotide binding domain (NBD)-transmembrane domain (TMD) topology with a 2-fold pseudosymmetry [NBD-TMD]_2_ typical for full-size fungal PDR transporters (Fig. 1). These transporters have six extracellular loops (ELs) and four intracellular loops (ILs) connecting 12 individual transmembrane spans (TMSs). There are four small ELs (EL1, -2, -4, -5) and two large ELs (EL3, -6), and two small ILs (IL2, -4) and two large ILs (IL1, -3), with family-defining PDR motifs (11) near the N termini of EL3 and EL6 (Fig. 1). These unique extracellular domains (EDs) are an attractive antifungal drug target because they are easily accessible to inhibitors (12), and inhibitors targeting them (12, 13) have potentially few side effects because humans lack PDR transporters (14, 15). PDR transporters such as C. albicans Cdr1 are characterized by (i) noninducible high basal ATPase activities (16); (ii) asymmetric NBDs with only one, composite NBD2 (CNBD2), able to hydrolyze ATP (11, 16, 17); (iii) a unique TMD-fold (9, 18–20); and (iv) a unique coupling mechanism between ATP hydrolysis and substrate translocation across the TMDs (9, 18, 19).

There are no atomic-level structures for any full-size plant or fungal PDR transporter, which significantly limits our understanding of this important multidrug efflux pump family. In the absence of high-resolution structures, biochemical analyses can provide important clues about the structure and function of a protein, and are essential for the confirmation of any structure. A number of alanine scanning and site-directed mutagenesis studies of Cdr1 and related ABC transporters have provided important insights into the drug binding pocket(s) (21), the mechanism of translocation (16, 22, 23), and how substrates (21) and/or inhibitors (12, 24–28) interact with Cdr1 and its Saccharomyces cerevisiae ortholog, Pdr5. Nevertheless, many aspects of Cdr1 biology remain obscure (9, 29).

C. albicans Cdr1 contains 23 cysteines; 10 are cytosolic, seven are in the TMDs, and six are in the EDs (Fig. 1). The six extracellular cysteines, two in EL3 and four in EL6 (Fig. 1), are conserved in fungal PDR transporters and predicted to form three extracellular disulfide bonds that stabilize the structure of PDR transporter EDs (11, 24). The existence of three possible disulfide bonds indicated that it may be difficult to create a fully functional cysteine-less (Cys-less) Cdr1 mutant. However, Prasad and colleagues reported the creation of a functional Cys-less Cdr1 molecule in 2012 (30). Most cysteines were replaced with alanine, but cysteines C1056, C1091, C1106, C1294, and C1336 (i.e., Cys 12 to 16; Fig. 1) had to be substituted with serine to obtain a functional efflux pump in S. cerevisiae. Unfortunately, this Cdr1 mutant turned out to be nonfunctional in our laboratory. This study describes the creation of a functional, “almost” Cys-less, Cdr1 molecule and the discovery of a novel motif that is conserved among all ABCG transporters and provides a pivotal contact point between the two NBDs at their cytosolic apex.

## RESULTS

### Characterization of Cys-deficient *CDR1* variants.

In order to determine the effect of replacing cysteine residues with either alanine or serine on Cdr1 expression and localization in S. cerevisiae, a green fluorescent protein (GFP) tag was fused to the C terminus of the *CDR1* open reading frame (ORF). The GFP tag did not affect the efflux pump function of Cdr1; i.e., the fluconazole (FLC) MICs (MIC_FLC_s) of ADΔΔ-CaCDR1P (256 mg/liter) and ADΔΔ-CaCDR1P-GFP (256 mg/liter) were indistinguishable. A plasmid containing the *CDR1-GFP* construct was used as a DNA template to replace cysteines with alanine or serine by PCR, prior to integrative transformation of S. cerevisiae ADΔΔ. To identify which cysteines were most critical for Cdr1 function, Cdr1 was divided into six subdomains, N1, TS1, EL3, N2, TS2, and EL6 (Table 1). The effect of substituting all 23 cysteines (Fig. 1) with either alanine or serine, decisions that were based on previous findings (30), in these subdomains and in all possible subdomain combinations was tested. Seventeen Cys-deficient *CDR1PC-GFP* variants were created (Table 1). A graphical representation of these variants is provided in Fig. S1 in the supplemental material. Substituting the cysteines of individual subdomains provided initial indications of which cysteines were most important for Cdr1 FLC efflux pump function. Surprisingly, substituting all five N1 or all five N2 cysteines actually increased (2-fold; N1) or had no noticeable effect (N2) on Cdr1 FLC efflux pump function (Table 1). However, substituting the two cysteines of TS1 and the five cysteines of TS2 caused 4- and 2-fold-reduced FLC efflux, respectively (Table 1). Even substituting the two conserved cysteines of EL3 had only minor (4-fold) effects. However, substituting the five cysteines of EL6 completely eliminated FLC efflux (Table 1). To assess the effects of combining individual Cys-deficient subdomains on the FLC pump function, we compared their MIC_FLC_s with those of the individual Cys-deficient subdomains. Combining cysteine substitutions of two or three subdomains of either the N- or C-terminal halves of Cdr1 had synergistic effects for most combinations (e.g., NTS1, NT1, NTS2) apart from the T1 variant, which gave the expected, additive, 16-fold-reduced FLC efflux pump function (i.e., 4- × 4-fold = 16-fold-reduced MIC_FLC_; Table 1). NTS1, NT1, and NTS2 had 4× (NTS1), 64× (NT1), and 8× (NTS2) reduced MIC_FLC_s, which were 2×, 8×, and 4× higher than expected for an additive effect (Table 1). For an additive effect of the NTS1 combination, for example, we would have expected a 2× reduced MIC_FLC_ (i.e., +2× improved × 4× reduced). Any subdomain combinations that included subdomains of both halves of the transporter (i.e., N12, TS12) had similarly strong synergistic effects (i.e., 4× and 2× larger than expected; Table 1). However, the NTS12 subdomain combination of NTS1 with NTS2 was additive with an expected MIC_FLC_ of 8 mg/liter (Table 1), and as expected, any subdomain combinations that included EL6 (T2, NT2, EL36, T12) had no FLC efflux pump function (Table 1).

**TABLE 1:** Phenotypes of ADΔΔ strains overexpressing *CDR1P*, *CDR1PC*, and 18 *CDR1PC*-*GFP* variants with the cysteines of the indicated *CDR1P* subdomains replaced with serine or alanine

*CDR1* construct^*a*^	Cdr1 subdomain^*b*^						MIC_FLC_ (mg/liter)	Fold reduced MIC_FLC_
	N1	TS1	EL3	N2	TS2	EL6		
*CDR1P*							256	1
*CDR1PC*							1	256
N1							512	+2^*d*^
TS1							64	4
EL3							64	4
NTS1^S^							64	4
T1^A^							16	16
NT1^SSS^							4	64
N2							256	1
TS2							128	2
EL6							1	256
NTS2^SS^							32	8
T2							1	256
NT2							1	256
N12^SS^							128	2
TS12^S^							16	16
EL36							1	256
NTS12^A^							8	32
T12							1	256
NTS12-S1^*c*^							128	2

a Superscript S, SS, and SSS indicate 2-fold, 4-fold, and 8-fold synergy, respectively, and superscript A indicates an additive effect on FLC transport of the indicated subdomain combinations (see text for further details).

b Gray areas indicate the various Cdr1 subdomains in which the cysteines have been replaced with alanine or serine. The position of individual cysteines and the residue they were replaced with (A or S) are as follows: T1, C193S, C325A, C363A, C402A, C462A; TS1, C632S, C635S; EL3, C712A, C732S; N1, C907A, C1041A, C1056S, C1091S, C1106S; TS2, C1294S, C1336S, C1357A, C1361A; EL6, C1380A, C1402A, C1418A, C1441S, C1444S.

c The ADΔΔ-CaCDR1PC-NTS12-GFP suppressor mutant was renamed ADΔΔ-CaCDR1-CID-GFP. It contained the S1106 of ADΔΔ-CaCDR1PC-NTS12-GFP replaced with I1106.

d +2 means twofold increased.

10.1128/mSphere.01318-20.3 FIG S1 Conserved extracellular loop cysteines (Cys) are critical for Cdr1 folding and/or function. All Cys of NBD1 (N1; 5), TMD1 (TS1; 2), or EL3 (E3; 2) or of NBD2 (N2; 5), TMD2 (TS2; 5), or EL6 (E6; 5) were replaced with Ala or Ser. The domains in which Cys have been replaced with Ala or Ser are indicated in gray, and their MIC_FLC_s are underneath each cartoon of the individual ADΔΔ-CaCDR1PC-GFP variants. NT1 and NTS12 variants were used to select for natural suppressor mutations (red dots) that could partially, or fully, recover Cdr1 function. The mutations are in critically important contact points for the opening and closing of the transporter. Download FIG S1, TIFF file, 2.0 MB. Copyright © 2021 Madani et al. 2021 Madani et al. https://creativecommons.org/licenses/by/4.0/ This content is distributed under the terms of the Creative Commons Attribution 4.0 International license.

These data revealed the EL6 cysteines as the most important residues for proper Cdr1 FLC efflux pump function. Substituting all 16 intracellular cysteines also caused serious disruptions to FLC transport in ADΔΔ/CaCDR1PC-NTS12—its FLC efflux pump function (i.e., MIC_FLC_ = 8 mg/liter) was 32-fold lower than wild-type Cdr1 (Table 1).

### Protein expression levels and ATPase activities of Cys-deficient *CaCDR1PC*-*GFP* mutants.

To assess the effects of the cysteine substitutions of the various *CDR1PC* constructs in more detail, crude plasma membranes were isolated and their Cdr1 expression levels and ATPase activities were determined.

**FIG 2:**
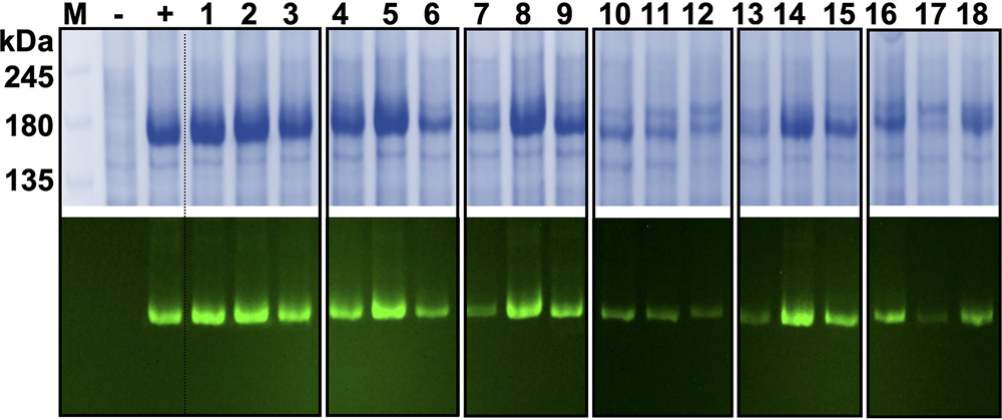
SDS-PAGE and in-gel fluorescence of plasma membrane samples isolated from ADΔΔ cells overexpressing 18 *CDR1PC*-*GFP* variants. SDS-PAGE (7% polyacrylamide) of crude plasma membrane samples (10 μg protein) isolated from ADΔΔ (−), ADΔΔ-CaCDR1P-GFP (+), and ADΔΔ cells overexpressing the *CDR1PC*-*GFP* variants: N-terminal variants N1 (1), TS1 (2), EL3 (3), T1 (4), NTS1 (5), and NT1 (6); C-terminal variants N2 (7), TS2 (8), EL6 (9), T2 (10), NTS2 (11), and NT2 (12); and variants of the indicated N- and C-terminal subdomain combinations N12 (13), TS12 (14), EL36 (15), T12 (16), NTS12 (17), and NTS12-S1 (18). In-gel fluorescence images of the same polyacrylamide gels before Coomassie blue staining are presented underneath; the fluorescence signals were used to quantify the Cdr1 expression levels (Table 2).

There were significant differences in the expression of the various Cdr1 constructs (Fig. 2 and Table 2). The expression levels of N-terminal Cdr1 variants N1 (Fig. 2, lane 1), TS1 (lane 2), and EL3 (lane 3) and the C-terminal variant TS2 (lane 8) were comparable to wild-type Cdr1 (Fig. 2). However, the expression levels of N2 (lane 7) and EL6 (lane 9) were reduced to 30% and 70% of that of wild-type Cdr1, respectively (Fig. 2). The expression of most Cdr1 variants with cysteines replaced in two (Fig. 2, lanes 4 to 6 and 10 to 12) or more (Fig. 2, lanes 13 to 17) subdomains was even more severely affected (i.e., reduced to 20 to 70% wild-type Cdr1) with the exceptions of NTS1 (Fig. 2, lane 5) and TS12 (Fig. 2, lane 14)—the expression levels of which were comparable to wild-type Cdr1 (Table 2).

**TABLE 2:** Cdr1 expression levels and ATPase activities of plasma membrane preparations isolated from ADΔΔ (negative control) and from ADΔΔ strains overexpressing *CDR1P-GFP* (positive control) and the *CDR1PC-GFP* Cys-deficient variants

Strain no.	Strain^*a*^	ATPase (nmol/min/mg)^*b*^^,^^*c*^		MIC_FLC_ (mg/liter)	Cdr1 expression^*d*^	Normalized^*e*^		
						ATPase		MIC_FLC_
	*CDR1P*	307 (14)		256	100	100		100
1	N1	440 (25)	***^g^	512	110	130	***	180
2	TS1	92 (20)	***	64	110	30	***	20
3	EL3	228 (42)	*	64	100	80	*	30
4	T1	−		16	70	−		10
5	NTS1	200 (28)	**	64	110	60	***	20
6	NT1	−		4	60	−		3
7	N2^*f*^	65 (19)	***	256	30	70	*	310
8	TS2	18 (9)	***	128	100	10	***	50
9	EL6	−		1	70	−		−
10	T2	−		1	40	−		−
11	NTS2^*f*^	11 (8)	***	32	30	10	***	40
12	NT2^*f*^	−		1	20	−		−
13	N12^*f*^	75 (10)	***	128	20	120	*	240
14	TS12	−		16	100	−		6
15	EL36	20 (3)	***	1	50	10	***	−
16	T12	−		1	40	−		−
17	NTS12^*f*^	−		8	20	−		10
18	NTS12-S1^*f*^	64 (11)	***	128	60	40	***	90

a The strain symbols are the same as in Table 1.

b The ATPase activities were corrected for the oligomycin-sensitive ADΔΔ background (22 ± 10 nmol/min/mg); values are the means (±SDs) of two technical replicates measured three times.

c The ATPase activities with a − sign were below the detection limit of the assay.

d Cdr1 expression levels (% relative to wild-type Cdr1P-GFP).

e Cdr1 ATPase activities and MIC_FLC_s (% relative to wild-type Cdr1P-GFP) normalized with respect to the different expression levels.

f Heat shock protein *SSA2* was upregulated in these strains (Fig. 4 and text give further details).

g Significant differences between the Cdr1 ATPase activities were determined with the Student *t* test: *, *P *≤ 0.05; **, *P *≤ 0.01; ***, *P *≤ 0.001.

To more accurately interpret the effects of the various cysteine substitutions on the FLC transport (MIC_FLC_s) and the ATPase activities in the Cdr1 mutants, their values were normalized to their expression levels (Table 2). For example, the Cdr1PC-N2 variant had an unchanged MIC_FLC_, yet its expression level was reduced by 70% (Fig. 2, lane 7, and Table 2). This variant was mainly affected in its expression level, but not much in its ATPase activity or its FLC efflux pump function (Table 2). Indeed, the normalized FLC efflux pump function indicated that this variant transported FLC ∼3 times more efficiently than wild-type Cdr1 (Table 2, *P* ≤ 0.05). Another interesting result was the comparison of the FLC efflux pump function of TS2 with that of N12 (Fig. 2, lanes 8 and 13). Both displayed a 2-fold-reduced MIC_FLC_ (128 mg/liter; Table 2), suggesting that the two were equally affected in FLC transport. However, after data normalization, it was evident that although TS2 had reduced FLC transport (∼50%, *P* ≤ 0.001), N12 transported FLC even more efficiently (240%, *P* ≤ 0.001) than wild-type Cdr1 because its expression level was dramatically reduced (∼20% of wild-type Cdr1; Table 2). Interestingly, the normalized *in vitro* ATPase activity of TS2 was even more dramatically affected (by ∼90%; Table 2). The TS1 construct (Fig. 2, lane 2) is a Cdr1 variant for which the reduced ATPase activity (30%) correlated well with its reduced FLC transport (20%; Table 2), whereas the EL3 variant (Fig. 2, lane 3) was minimally affected in its ATPase activity and yet its FLC transport (30%) was as low as TS1; neither TS1 nor EL3 was noticeably affected in its expression level (Table 2).

Thus, it would seem that substituting the two EL3 cysteines was not particularly detrimental to Cdr1 expression/function, but combining this with TS1 (T1) or NTS1 (NT1) almost completely inactivated Cdr1 even though these variants were still expressed quite well (60 to 70% of wild-type Cdr1). Substituting the four conserved EL6 cysteines led to an inactive protein in all EL6-containing variants despite reasonably good expression levels (20 to 70% of wild-type Cdr1; Table 2). In other words, any cysteine-deficient subdomain that was combined with the EL3- and/or EL6-deficient subdomains was almost (EL3) or completely (EL6) inactive (i.e., no detectable ATPase activity and MIC_FLC_ of <16 mg/liter; Table 2), while the function of most other variants, with the exception of TS12 and NTS12, was much less severely affected. Interestingly, substituting the cysteines in the cytosolic NBDs of the N1, N2, and N12 variants improved (2- to 3-fold) FLC transport although their ATPase activities and expression levels were similar to, or even lower than, wild-type Cdr1 (Table 2).

These results demonstrated the importance of the six extracellular cysteines for the structural and functional integrity of Cdr1. The cysteines of NBD1, and particularly those of NBD2, however, were also quite important. While substituting the cysteines in NBD1 and/or NBD2 significantly improved (2- to 3-fold) FLC transport, substituting the five NBD2 cysteines severely reduced Cdr1 expression, suggesting an unstable protein with improved FLC efflux. While the expression of the third group of mutants, with cysteine substitutions in the TMS regions of Cdr1 (TS1, TS2, and TS12), remained unchanged, their ATPase activities and/or FLC efflux pump function were severely reduced (TS1, TS2) or almost completely eliminated (TS12).

### Confocal microscopy of yeast cells expressing Cdr1PC-GFP variants.

**FIG 3:**
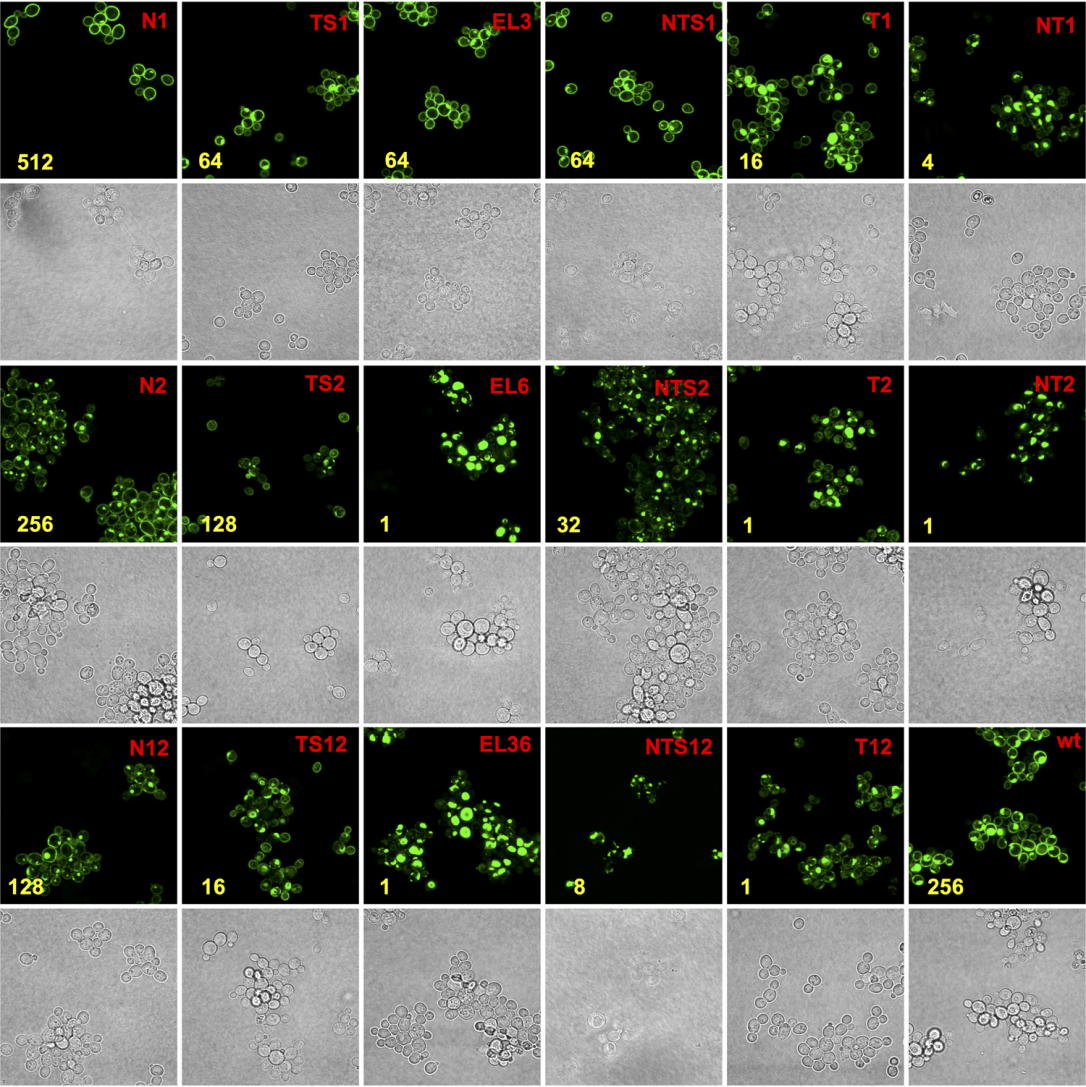
Confocal microscopy of ADΔΔ cells overexpressing Cdr1PC-GFP variants. ADΔΔ/CaCDR1P-GFP (wt; bottom right) was included as a positive control. Brightfield and fluorescent images of cells expressing the N-terminal variants are at the top, images of cells expressing the C-terminal variants are displayed underneath, and variants of N- and C-terminal subdomain combinations are shown at the bottom; see text for further details. MIC_FLC_ values (mg/liter) are indicated in yellow. To allow fair comparisons, all images were taken with identical confocal microscope settings.

Further information about the effects of cysteine substitutions on the folding, expression, and/or function of Cdr1 came from studying Cdr1 localization with confocal microscopy (Fig. 3 and Fig. S2). Cdr1 variants N1, TS1, EL3, and NTS1 with cysteine substitutions in the N-terminal subdomains were properly localized at the plasma membrane (Fig. 3). All other Cdr1 variants showed significant intracellular accumulation. Generally, the more Cdr1 they accumulated inside the cell, the more severely the variants were affected in FLC transport and/or expression. Although the EL3 Cdr1 variant properly localized to the plasma membrane, combining EL3 with TS1 (T1) or NTS1 (NT1) had a detrimental effect on the plasma membrane localization of Cdr1 (Fig. 3). Minor effects on the localization of Cdr1 were also observed for the C-terminal subdomains N2 and TS2 and for TS12, but all EL6 subdomain variants of Cdr1 (EL6, T2, NT2, EL36, and T12) appeared completely stuck inside the cell (Fig. 3). There were two distinct groups of mutants: group I (T1, NT1, EL6, T2, NT2, EL36) had most Cdr1 in one large aggregation near the center of the cell and group II (N2, TS2, NTS2, N12, TS12, T12, NTS12) showed a more diffuse localization pattern with smaller aggregates scattered throughout the cell.

10.1128/mSphere.01318-20.4 FIG S2 Confocal microscopy of ADΔΔ-CaCdr1PC-GFP variants shown in Fig. S1. MIC_FLC_s (mg/liter) are shown in yellow. Download FIG S2, TIFF file, 2.7 MB. Copyright © 2021 Madani et al. 2021 Madani et al. https://creativecommons.org/licenses/by/4.0/ This content is distributed under the terms of the Creative Commons Attribution 4.0 International license.

### Heat shock protein HSP72, Ssa2, is upregulated in N2 subdomain containing Cdr1PC-GFP variants.

**FIG 4:**
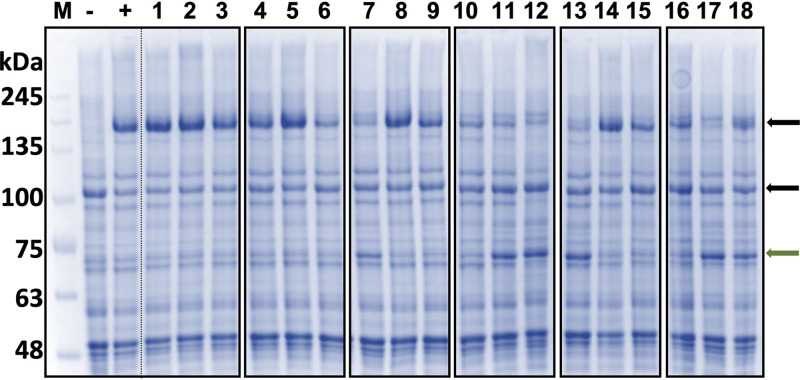
Plasma membrane protein profiles of ADΔΔ cells overexpressing cysteine-deficient Cdr1PC-GFP variants. −, ADΔΔ; +, ADΔΔ-CDR1P-GFP; variant numbers (1 to 18) are the same as in Fig. 2 and Table 2. The major plasma membrane proton pump, Pma1 (∼110 kDa) and Cdr1-GFP (∼200 kDa), bands are indicated with black arrows. Some lanes (7, 11, 12, 13, 17, and 18; N2, NTS2, NT2, N12, NTS12, and NTS12-S1 [i.e., CID], respectively) had a noticeably upregulated ∼70-kDa protein band (Ssa2; green arrow; see Table S2 in the supplemental material). M, Precision Plus protein marker.

Close inspection of the Coomassie blue-stained SDS-PAGE gel of plasma membrane proteins from the 18 cysteine-deficient *CDR1PC*-*GFP* variants identified a prominent ∼70-kDa band significantly upregulated in all N2 subdomain-containing strains (N2, NTS2, NT2, N12, NTS12, and NTS12-S; Fig. 4). Matrix-assisted laser desorption ionization–time of flight/time of flight (MALDI-TOF-TOF) analysis identified the ∼70-kDa band as the yeast heat shock protein HSP72 (Swiss-Prot search), also known as Ssa2 (Table S2). Ssa2 is a highly conserved protein involved in protein refolding and trafficking, ubiquitin-dependent degradation, and vacuolar import of abnormal proteins (31, 32).

It appears that any Cdr1PC-GFP variants with their N2 subdomain cysteines replaced were unstable, and Ssa2 was upregulated to possibly rescue these mutants from degradation. Despite these rescue efforts, most N2-containing variants had severely reduced expression levels (20 to 30%; Table 2). The reduction of Cdr1 expression in NTS12-S1 was less marked (60% of wild-type Cdr1), and Ssa2 was also less prominent (Table 2 and Fig. 4).

### Creation of an almost Cys-less, but functional, Cdr1 molecule.

Based on our investigation of the contribution of cysteines to Cdr1 trafficking and activity, we attempted to create a Cys-less, but functional, Cdr1 molecule. CDR1PC-NT1, with the nine N-terminal cysteines replaced with serine or alanine, was severely affected in its FLC transport, but it could still efflux FLC to some extent (∼3%; Table 2). Therefore, naturally arising ADΔΔ-CaCDR1PC-NT1-GFP suppressor mutants that could rescue its efflux deficiency were selected on plates containing growth-inhibitory concentrations of FLC (20 mg/liter). Four suppressor mutants were isolated, and each was found to have acquired a single point mutation in *CDR1*; suppressor mutant S1 had acquired A1207T, S2 had acquired G521R, and S3 and S4 had each acquired V693L (Table 3 and Fig. S1 and S2). Interestingly, they were either completely (S1) or almost completely (S2 to S4) restored in their ability to efflux FLC, although their transport efficiencies for six different Cdr1 efflux pump substrates varied (Table 3). The CDR1PC-NT1-S1 efflux pump function was most like wild-type Cdr1. Unfortunately, adding the five cysteine substitutions of subdomain N2 to CDR1PC-NT1-S1 (-S1-N2; Table 3 and Fig. S1 and S2) had a very strong synergistic effect resulting in an MIC_FLC_ indistinguishable from that of CDR1PC-NT1 (4 mg/liter) even though either variant alone had wild-type MIC_FLC_s (256 mg/liter; Table 3). Clearly, creating a fully functional Cys-less Cdr1 molecule with such an iterative approach was not possible. The destabilizing effect of N2, discussed above, was evident when N2 was combined with CDR1PC-NT1-S1.

**TABLE 3:** Drug susceptibilities of ADΔΔ-CaCDR1PC-NT1 and -NTS12 suppressor mutants

Strain^*a*^		Mutation	MIC (fold increase)^*b*^						
			ANI	CHX	FLC	CLT	R6G	KTC	NIG
ADΔΔ-CaCDR1P			32	128	256	4,096	64	512	ND
-CaCDR1PC-NT1			2	4	4	64	16	4	ND
-NT1-S1		A1207T	64	128	256	1,024	32	256	ND
-NT1-S2		G521R	16	16	128	32	8	16	ND
-NT1-S3 and -S4		V693L	16	64	128	2,048	128	256	ND
-NT1-S1-N2		A1207T	ND^*c*^	ND	4	ND	ND	ND	ND
-CaCDR1PC-NTS12			ND	ND	8	ND	ND	ND	ND
Group 1	-S1^*d*^	S1106I	64	64	128	1,024	128	256	256
	-S2	S1106I	64	64	128	1,024	128	256	256
	-S3	S1106C	64	64	128	1,024	128	256	256
	-S15	S1106C	64	64	128	1,024	128	256	256
Group 2	-S9	ΔGA4488-9^*e*^	16	32	64	512	64	64	128
	-S10	iA4496^*f*^	16	32	64	512	64	64	128
	-S11	ND	16	32	64	512	64	64	128
	-S12	ND	16	32	64	512	64	64	128

a All *CDR1* variants had a C-terminal GFP tag.

b The MICs for the sensitive control strain ADΔΔ were 0.25 (ANI), 0.015 (CHX), 1 (FLC), 0.002 (CLT), 0.5 (R6G), 0.008 (KTC), and 0.25 (NIG) mg/liter, respectively.

c ND, not determined.

d This strain was renamed ADΔΔ-CaCDR1-CID-GFP.

e Deletion of “GA” causing frameshift and premature termination of Cdr1.

f Insertion of “A” causing frameshift and premature termination of Cdr1.

We therefore decided to create an “almost” Cys-less, but functional, Cdr1 molecule that had all but the six extracellular cysteines substituted with serine or alanine. Such a mutant could still be used for cysteine-cross-linking studies because the six extracellular cysteines form three disulfide bonds (24) which do not interfere with cysteine-cross-linking reactions (33). A search for natural suppressor mutants of ADΔΔ-CaCDR1PC-NTS12 on agar containing FLC (20 mg/liter) produced 15 isolates, of which eight were further characterized. The isolates separated into two distinct groups: four group 1 mutants, which had a single point mutation in S1106 (Table 3), which was reverted back either to C1106 (S3, S15) or to I1106 (S1, S2), and four group 2 mutants, two of which (S9, S10) were sequenced. Both had frameshift mutations that caused a premature stop codon near the end of Cdr1 and, thus, the removal of the last three (S9) or two (S10) Cdr1 residues and the C-terminal GFP tag (Table 3). These results indicated that the C1106S mutation was the major reason for the instability observed in N2 subdomain-containing variants. CDR1PC-NTS12-S1 was renamed CDR1-CID (Cys-less Intracellular Domain).

**FIG 5:**
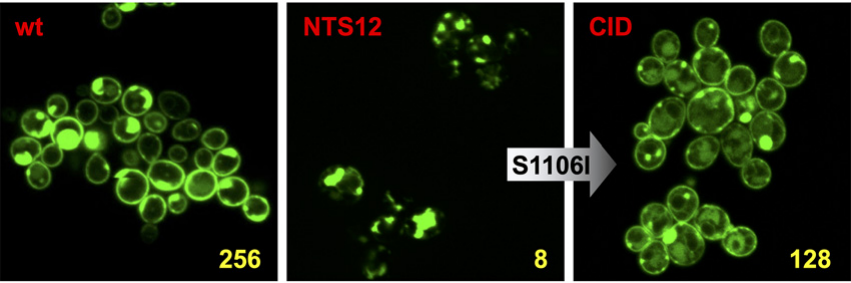
Confocal microscopy of ADΔΔ-CDR1P-GFP (wt), ADΔΔ-CDR1PC-NTS12-GFP, and ADΔΔ-CDR1-CID-GFP. The point mutation S1106I (arrow) recovered plasma membrane localization and function in CDR1PC-NTS12-S1-GFP (i.e., CID). MIC_FLC_ values (mg/liter) are shown in yellow.

CDR1-CID was, however, slightly different from Cdr1 in its transport efficiencies. Relative to Cdr1, CDR1-CID transport efficiency was 2-fold increased for anisomycin (ANI) and rhodamine 6G (R6G); 2-fold reduced for cycloheximide (CHX), FLC, and ketoconazole (KTC); and 4-fold reduced for clotrimazole (CLT) (Table 3). In comparison to CDR1-CID, removal of the C-terminal GFP tag in the group 2 mutants (S9 and S10) did not recover as much wild-type Cdr1 function (Table 3). The normalized FLC efflux pump function of CDR1-CID was comparable to wild-type Cdr1, but its expression level (∼60%) and ATPase activity (∼40%) were slightly reduced (Table 2). The localization of Cdr1-CID was also altered and similar to CDR1PC-N2 and CDR1PC-NTS2 (Fig. 3 and 5). The slight mislocalization of CDR1-CID correlated with a small upregulation of Ssa2 (Fig. 4).

### Cdr1-C1106 is part of a conserved motif at the cytosolic apex of ABCG transporters.

It was apparent that replacing S1106 with either C1106 or I1106 was critical for restoring Cdr1 function. We noticed that Cdr1-C1106 was only three amino acids N-terminal of the recently discovered NPXD/E motif that is conserved in ABCG half transporters (18). This motif is thought to provide an important contact point between the two NBDs converging at the cytoplasmic apex of the inside-open conformation of the human sterol transporter, ABCG5/8 (18). Others have noted a conserved NPADF motif in human ABCG1 (34) that mediates cholesterol efflux onto lipoprotein A particles.

**FIG 6:**
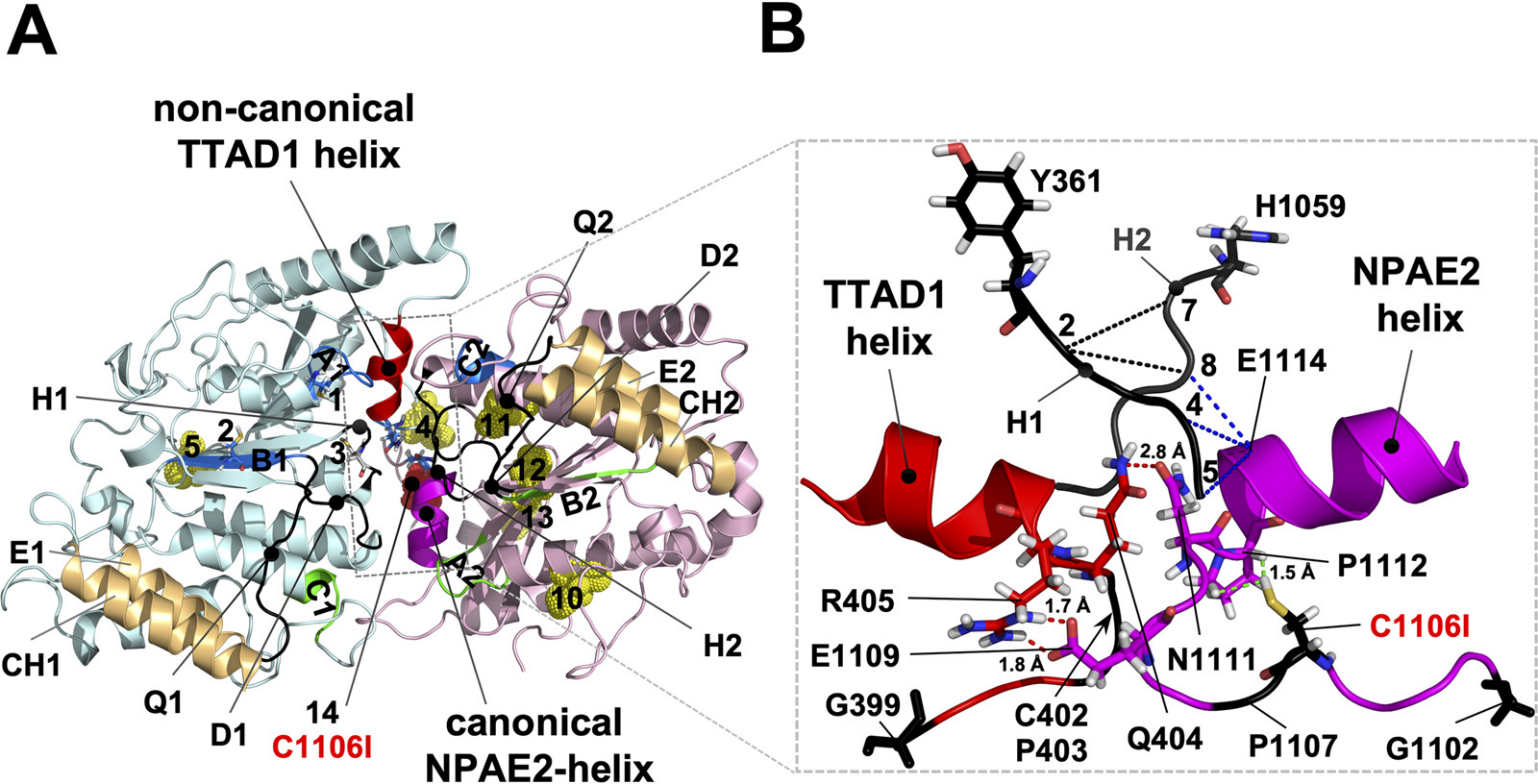
Architecture of the GX_2[3]_CPX_3_NPAD/E loop-helix motifs that are in contact with each other at the cytosolic apex between the two NBDs of Cdr1. (A) Model, viewed from the top, of the nucleotide-free conformation of the NBDs of Cdr1 (24) based on the ABCG5-G8 structure (18); the TMDs were removed for clarity. The N- (turquoise) and C-terminal (pink) halves are color coded. The characteristic signature motifs of the noncanonical CNBD1 (A1, B1, C2; for abbreviations see Fig. 1) and the catalytically active canonical CNBD2 (A2, B2, C1) are shown in blue and green, respectively, and the Q-, D-, and H-loops (Q1, Q2, D1, D2, H1, and H2) are in black and indicated with lines pointing toward their center. The N-terminal (4 and 5) and C-terminal (10 to 13) cysteines are shown as yellow dots; the N-terminal cysteines 1 to 3 that are part of A1, B1, and H1, respectively, are shown as sticks; and C1106 (14) is highlighted as a red dot near the center of the converging NBDs. The coupling helices (CH1 and CH2), unique ABCG transporter features which connect TMD1 and TMD2 with the E-helices (E1 and E2) of NBD1 and NBD2, respectively, are highlighted brown. The two GX_2[3]_CPX_3_NPAD/E loop-helix motifs providing tight contact between the two NBDs at the cytosolic apex are highlighted red (NBD1; degenerate motif; TTAD1 helix) and magenta (NBD2; canonical motif; NPAE2 helix). The canonical NPAE2 helix on the edge of one half of the centrally located NBD1-NBD2 contact region is positioned right underneath the canonical CNBD2, and the noncanonical TTAD1 helix (red) on the edge of the other half of the NBD1-NBD2 contact region is positioned right underneath the noncanonical CNBD1. (B) Close-up side-on view of the peripheral contact region near the center of the NBDs delineated with dashed gray lines in panel A. To show how H1 and H2 interact closely with the conserved contact motifs underneath, the remainder of the NBDs were removed. The noncanonical H1-Y361 and the canonical H2-H1059 are shown as black sticks. The six conserved CP and G residues of the noncanonical NBD1 (red) and the canonical NBD2 (magenta) GX_2[3]_CPX_3_NPAD/E motifs are in black (C402, P403, P1107) with the two N-terminal G residues (G399 and G1102) and C1106 shown as sticks. Residues of the loop regions that are in close proximity (<3 Å) near the center of the two NBDs are also shown as sticks: Q404 is in close contact with N1111 (red dashed line), R405 forms a possible salt bridge (red dashed lines) with E1109, and C1106 is in close contact (green dashed lines) with P1112. C1106 is part of CP2, and N1111 and P1112 are part of the canonical NPAE2 helix. Close contacts (<3 Å) between H1 and H2 with E1114 of the NPAE2 motif are indicated with black (H1-H2) and blue (H1-E1114 and H2-E1114) dashed lines, respectively. The five H1 residues YQCSQ were numbered 1 to 5, and the five H2 residues HQPSAL were numbered 6 to 11, respectively. Further details of the architecture surrounding this region are provided in Fig. S5 in the supplemental material.

A detailed analysis of the region surrounding the NPXD/E motif in plant, fungal, and human ABCG/WBC half transporters with the equivalent NBD1 and NBD2 regions of a representative set of full-size plant and fungal PDR transporters is presented in Fig. S3. The phylogenetic relationships of the transporters are shown in Fig. S4. There was a clearly defined, and conserved, 8- or 9-amino-acid loop comprising an N-terminal G residue, two or three random (X_2[3]_) residues, two highly conserved C and P residues, and a further three random (X_3_) residues (i.e., GX_2[3]_CPX_3_). A conserved 10-residue alpha-helix including the N-terminal NPXD/E motif followed (Fig. 6; i.e., GX_2[3]_CPX_3_NPXD/E). This motif was typically 37 residues C-terminal of the H-loop H residue in ABCG/WBC half transporters. It was also 37 residues C-terminal of the NBD1 H-loop H in symmetric fungal cluster F PDR transporters, the common ancestor of all plant and fungal PDR transporters (Fig. S4), and the noncanonical H-loop “switch” residues L and Y in plant and fungal PDR transporters (Fig. 1), respectively. In the NBD2 regions, however, the motif was typically 38, 42, or 43 residues C-terminal of the canonical H-loop H’s in fungal cluster F PDR transporters and in plant and fungal PDR transporters. All PDR transporters had an additional conserved G residue (i.e., Cdr1-G1078) inserted just before, and plant and fungal PDR transporters typically had four more residues (i.e., GX_2_G; Cdr1-G1085-G1088) inserted just after, beta-sheet 5 (a model of where these five additional NBD2 residues were inserted in Cdr1 is shown in Fig. S5). Although there were clear variations in the newly discovered GX_2[3]_CPX_3_NPXD/E loop-helix motif, individual variations were shared within the various groups of ABCG transporters (see Fig. S3 for further details). The most notable variations were a “degenerate” NBD1-NPXD/E motif (red helices in Fig. S3) in fungal and plant PDR transporters. There were also significant changes to the canonical GX_2[3]_CPX_3_NPXD/E motif in the NBD2 of full-size ABCG transporters. Fungal cluster C, D, and G PDR transporters, for instance, were the only ABCG transporter families that lacked the “conserved” P residue, and cluster G PDR transporters also lacked the “conserved” G residue in the NBD2-GX_2[3]_CPX_3_NPXD/E motif (Fig. S3), whereas plant PDR transporters had the typically conserved C, P, and negatively charged D/E residues replaced with a small hydrophobic (h; mostly an I), a random (X), or a T or A residue, and they had two additional residue insertions, one just before the conserved G (often a P) and the other just before the conserved small hydrophobic h residue (either a K or P; fungal cluster A, B, C, D, and some H1 PDR transporters shared a similar insertion just before their conserved C residue; Fig. S3).

10.1128/mSphere.01318-20.5 FIG S3 Sequence alignments of the GX_2[3]_CPX_3_NPAD/E loop-helix motif(s). The figure shows the alignment of the highly conserved GX_2_CPX_3_NPAD/E loop-helix motif of ABCG/WBC half transporters (top three panels) with the corresponding N-terminal (NBD1; left panels) and C-terminal (NBD2; right panels) regions of full-size ABCG transporters. The ABCG transporters are grouped into plant, fungal, and human ABCG/WBC half transporters (the fungal ABCG half transporters are C. albicans 19.3120 orthologs). Full-size ABCG transporters are also commonly known as PDR transporters. Fungal cluster F PDR transporters (F_F_; i.e., S. cerevisiae YOL075C orthologs) are the common ancestor of all plant and fungal PDR transporters (PDR_anc_). Representative plant and fungal PDR transporter subclusters F_A_, F_B_, F_C_, F_D_, F_G_, F_H1_, and F_H2_ (11) are shown. Fungal cluster E (F_E_) PDR transporters, which are cholesterol importers, were excluded from the alignment. The alignments were created with ClustalW (default settings), and the ClustalX color scheme of the JalView sequence editing software was chosen to display sequence conservation. Plant PDR transporters had one additional residue inserted just before the conserved G residue of the GX_2[3]_CPX_3_NPAD/E motif (see NBD2 alignments above). Numbers to the left denote the first residue number of individual sequences. The types of residues conserved in individual positions of the GX_2[3]_CPX_3_NPAD/E motif are displayed separately underneath their alignments for each group of ABCG transporters. A magenta helix indicates a highly conserved NPAD/E helix motif whereas red helices emphasize the noncanonical, degenerate, nature of the “NBD1-NPAD/E” helices of plant and fungal PDR transporters. The lines and numbers between the conserved G, CP, and NPAD/E motifs represent the numbers (2 or 3) of residues inserted between them for the various groups of ABCG transporters. X = any, and h = small hydrophobic, amino acids (see text for further details). The phylogenetic relationship and a list of the individual sequences with species abbreviations can be found in Text S1. Download FIG S3, TIFF file, 2.7 MB. Copyright © 2021 Madani et al. 2021 Madani et al. https://creativecommons.org/licenses/by/4.0/ This content is distributed under the terms of the Creative Commons Attribution 4.0 International license.

10.1128/mSphere.01318-20.6 FIG S4 Maximum likelihood tree of representative plant, fungal, and human half ABCG/WBC transporters (A) and full-size plant and fungal PDR transporters (B). The maximum likelihood trees for representative plant, fungal, and human ABCG/WBC half transporters (A) and full-size plant and fungal PDR transporters (B) that were used in Fig. S3 are midpoint rooted. (A) Display of the phylogenetic tree for the selected plant (green), fungal (brown), and human (blue) ABCG/WBC half transporters. Although fungal ABCG half transporters separate into a clearly distinct clade, plant and human ABCG half transporters do not divide into distinct plant and human clades, indicating multiple common ancestors of ABCG half transporters that cross major species boundaries. (B) Full-size PDR transporters divide into three major types (100% bootstrap support) of symmetric fungal cluster F (F_F_; brown) PDR transporters (i.e., S. cerevisiae YOL075C orthologs; the common ancestor of all PDR transporters) and typical asymmetric plant (green) and fungal PDR transporters. Fungal *sensu stricto* PDR transporters separate into eight major clusters (F_A_ [red], F_B_ [purple], F_C_ [orange], F_D_ [magenta], F_E_ [not shown], F_G_ [blue], F_H_) with cluster H PDR transporters further dividing into two distinct subclusters H_1_ (light blue) and H_2_ (light green) (11). Arrows 1 to 3 point to times when the unique degenerate characteristics of the GX_2[3]_CPX_3_NPA[D/E] motifs evolved in plant and fungal PDR transporters (see text for further details). The scale bar indicates the number of amino acid substitutions per position. Bootstrap support for 100 replicates is shown above individual branches. Download FIG S4, TIFF file, 1.9 MB. Copyright © 2021 Madani et al. 2021 Madani et al. https://creativecommons.org/licenses/by/4.0/ This content is distributed under the terms of the Creative Commons Attribution 4.0 International license.

10.1128/mSphere.01318-20.7 FIG S5 Model of the GX_2[3]_CPX_3_NPAD/E loop-helix motifs and surrounding regions of the NBDs of Cdr1. Panels A and B are a front and rear view, respectively, of the model presented in Fig. 6 including the regions surrounding the NBDs of Cdr1 to show how these motifs interact with the NBDs. The helical regions (i.e., residues between Walker A and the ABC signature motif) above the ATP-binding site were removed for clarity, but the E-helices just after the Q-loop that are in close contact with the CHs of the TMDs were included to provide context. The color codes, important contact regions, lines, and descriptions are the same as in Fig. 6 (see text for further details). Download FIG S5, TIFF file, 2.6 MB. Copyright © 2021 Madani et al. 2021 Madani et al. https://creativecommons.org/licenses/by/4.0/ This content is distributed under the terms of the Creative Commons Attribution 4.0 International license.

### The two GX_2[3]_CPX_3_NPXD/E motifs provide a network of contacts between each other and the H-loops that converge at the cytosolic apex of Cdr1.

PDR transporters are asymmetric, hydrolyzing only one ATP at CNBD2 (green regions; Fig. 6A) per transport cycle while ATP remains bound to, but is not hydrolyzed (16, 17) at, CNBD1 (blue regions; Fig. 6A). It would seem that the degenerate TTAD1 helix (red in Fig. 6 and Fig. S3) just underneath CNBD1 (Fig. 6A) may have evolved to accommodate the characteristic asymmetric nature of plant and fungal PDR transporters. A close-up side-on view of the centrally located contact region and key interacting residues is shown in Fig. 6B. Cdr1-Q404 is in close contact (<3 Å) with N1111 of Cdr1-NPAE2 where the degenerate TTAD1 and NPAE2 helices meet. The critically important C1106 residue, which is part of the CP2 loop-motif (i.e., C1106 and P1107), is in close contact with P1112 of NPAE2. A third notable interaction is a possible salt bridge between R405 and E1109 (Fig. 6B). Cdr1-Q404 is not well conserved, unlike N1111, suggesting that this interaction may not be as important as the R405-E1109 interaction. These two residues are highly conserved (almost invariably an R and a D or E) in fungal cluster A, B, and C PDR transporters (Fig. S3). Interestingly, in plant PDR transporters the C1106 equivalent residue is almost invariably an I, but P1112 is conserved (Fig. S3). This supports our findings that replacing C1106 with I1106 is the “preferred option” for keeping the C1106-P1112 interaction intact. Replacing C1106 with S1106 in N2 subdomain-containing *CDR1PC*-*GFP* variants had devastating effects on the protein. Cdr1-Q404 was also significantly further apart (2.8 Å) from Cdr1-N1111 than the other two, possibly more important, contact pairs (i.e., R405-E1109 [1.7 Å] and C1106-P1112 [1.5 Å; Fig. 6B]). The two tightly interacting Cdr1-loop-helix motifs were closely tucked in between a cover of five beta-sheets on one side, provided by their own respective NBDs, and a helical region underneath and along the other side provided by the opposing NBD (a clear view of that region is provided in Fig. S5). The beta-sheets of the two NBDs are named β1 to β5 (NBD1) and β1′ to β5′ (NBD2), respectively. The single G residue (G1078) just before β5′ is conserved in all full-size ABCG transporters, and the four-residue loop-insertion (red GX_2_G; i.e., G1085-X_2_-G1088) between β5′ and the conserved alpha-helix just before the GX_2[3]_CPX_3_NPAD/E loop-helix motif is a characteristic insertion found only in plant and fungal PDR transporters (see Fig. S5). But what is the significance of one of the most conserved negatively charged residues, the D/E of the GX_2[3]_CPX_3_NPXD/E motifs? Only plant PDR transporters had T or A residues in its place in their canonical NPA[T/A]2 motif (Fig. S3). There were no obvious residues in close proximity (<3 Å) of D410 of the degenerate Cdr1-TTAD1 helix. Cdr1-E1114 of NPAE2, however, was in close contact with three H-loop residues (blue dashed lines, Fig. 6B): H-loop 1 residues 4 (S364) and 5 (Q365) and H-loop 2 residue 8 (P1061). Perhaps the conserved D/E residues of the NPXD/E motif provide important contacts to the H-loop switch regions during the ABCG transport cycle.

## DISCUSSION

This study was initiated to create a Cys-less version of Cdr1 that maintains its pump function. A Cys-less, or nearly Cys-less, protein provides a powerful tool to assess structural and mechanistic aspects of multidrug efflux pumps (33, 35–37). We found that substituting N-terminal cysteines was less detrimental to expression, folding, and/or function than substituting C-terminal cysteines. Replacing the four conserved EL6 cysteines was most detrimental. It eliminated plasma membrane localization and Cdr1 function. The four conserved EL6 cysteines are obviously critically important for the structural integrity of Cdr1. Conservative substitution of these residues caused the structural destabilization and/or partial misfolding of Cdr1, and the protein did not reach the plasma membrane. Substituting the five cysteines of the C-terminal NBD2 (N2) also had an effect on the stability of Cdr1, but it was less severe. Most CDR1PC-N2-GFP protein reached the plasma membrane, and it was even more efficient (3 times) in FLC transport than wild-type Cdr1, as its expression level was severely (3-fold) reduced. Further analysis revealed that in all N2 subdomain-containing *CDR1* variants, Ssa2 expression was upregulated. S. cerevisiae Ssa2 is a subunit of the chaperonin-containing T-complex and an important HSP72 family member. It has a protein refolding activity in protein translocation, and it is also required for the ubiquitin-dependent degradation of short-lived proteins (31, 32).

**FIG 7:**
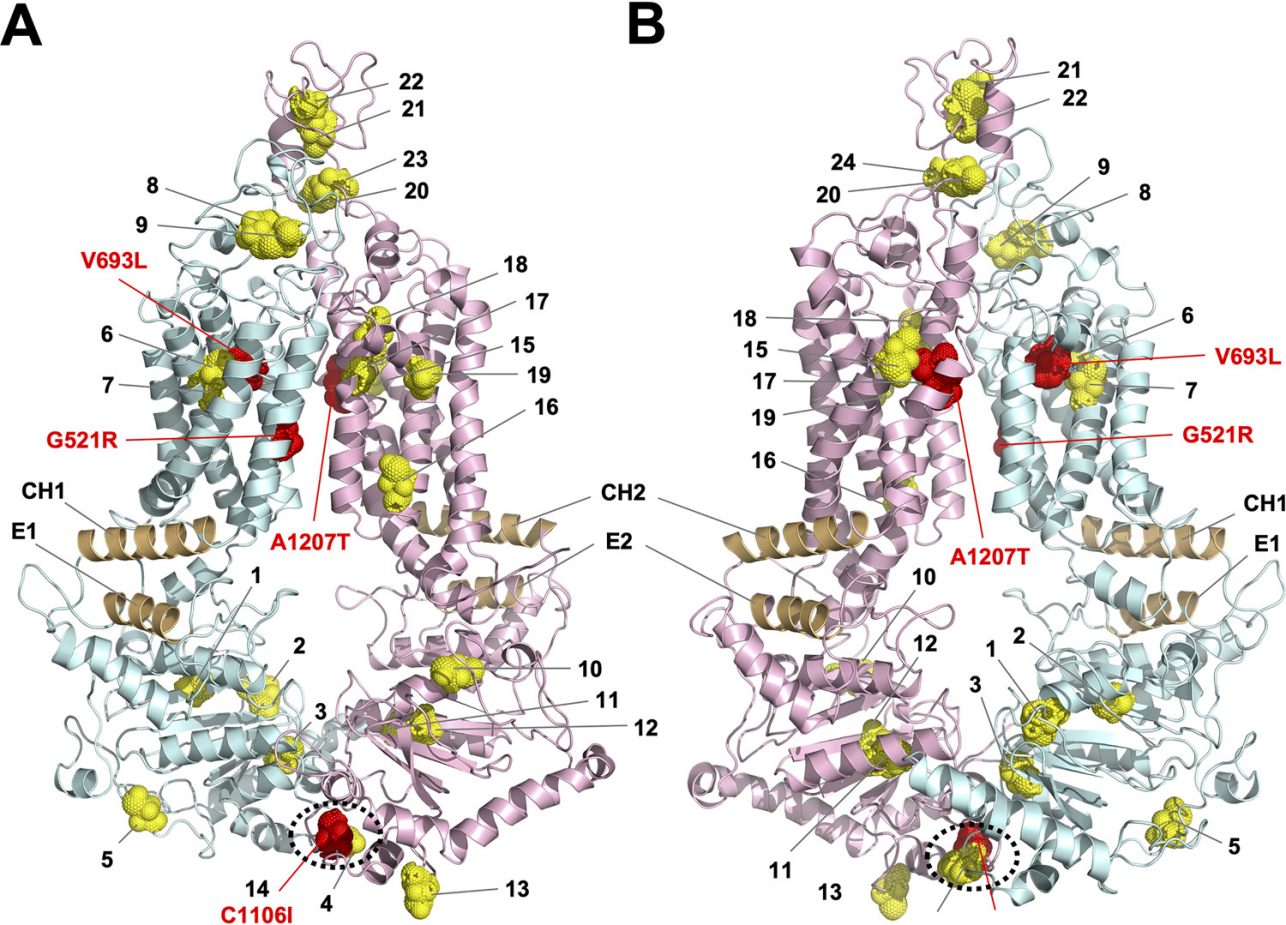
Location of the 23 cysteines and key residues (red) critically important for recovering efflux pump function of Cdr1PC-GFP variants. Model of Cdr1 (24) based on the ABCG5-G8 structure (18). The N-terminal (turquoise) and C-terminal (pink) halves are color coded, and the 23 cysteines (1 to 23) are shown as yellow dots, apart from C1106 (14), which is red. The model is viewed from the front (A) or back (B). The coupling helices (CH1 and CH2), unique PDR transporter features that connect TMD1 and TMD2 with the E-helices (E1 and E2) of NBD1 and NBD2, respectively, are highlighted brown. The characteristic contact points at the bottom of the two NBDs are encircled with black dotted lines (A and B). Mutations of key contact residues that could recover the function of various Cys-less Cdr1 variants are shown in red.

Initially, we attempted to create a Cys-less version of Cdr1 that remained functional, by iteratively combining Cys-less, but functional, Cdr1 subdomains. However, combining these Cys-less subdomains reduced Cdr1 activity greatly. The four CDR1PC-NT1 suppressor mutants isolated in an attempt to recover activity had point mutations in G521R, V693L, and A1207T. The mutation identities and location were revealing (Fig. 7). G521 and A1207 are pseudosymmetric residues at the center of TMS1 and TMS7, respectively, and V693 is part of PDRB (11). G521 and A1208, one residue C-terminal of A1207, were previously recognized as key residues in the interdomain communication pathway that allows Cdr1 to transport various substrates through the substrate transport channel (38). Changing these residues affected the substrate specificity of Cdr1 (38). V693, at the N terminus of PDRB, may be another key residue for the opening and closing of Cdr1. Cdr1-V693 is near the central binding pocket between TMS2, TMS4, and TMS5 and close to cysteines 6 (C632) and 7 (C635), which are in the center of TMS4 (Fig. 7B). We speculate that G521 and A1207/A1208 are critical contact points between TMD1 and TMD2 and that V693 is possibly a key residue for correct realignments of TMS2, TMS4, and TMS5 during opening and closing of Cdr1. The 9 cysteine substitutions in CDR1PC-NT1 possibly affected the evolutionarily conserved interdomain communication and led to the collapse of the ATPase activity and efflux pump function. This defect could, however, be rescued to some degree by changing just one of three key residues (G521R, A1207T, V693L). These changes, however, also affected the substrate specificity of Cdr1; each suppressor mutant exhibited a significantly altered substrate specificity. Unfortunately, combining CDR1PC-NT1-S1 with the N2 Cys-less subdomain reverted the MIC_FLC_ of CDR1PC-NT1-S1-N2 back to a level (4 mg/liter) indistinguishable from that of CDR1PC-NT1.

Therefore, we decided to create an “almost Cys-less” version of Cdr1 instead. Isolating suppressor mutants of CDR1PC-NTS12 revealed there were only two options for the mutant to regain efflux pump function. One was the removal of the C-terminal GFP tag (group 1 mutants; Table 3), and the other was to revert S1106 back either to its wild-type residue, C1106, or to I1106 (group 2 mutants; Table 3). It appears that allosteric interference of the C-terminal GFP tag was sufficient to destabilize CDR1PC-NTS12, although it had no measurable effect on wild-type Cdr1. We previously reported inactivation of wild-type Cdr1 by a C-terminal GFP tag in the related *Candida* species, C. utilis (39). This defect could, however, be overcome by adding a small linker between the protein and the GFP tag. Together, these findings suggest that (i) the C1106S substitution was most likely the main reason for the destabilization of CDR1PC-GFP variants containing N2 subdomain Cys replacements and (ii) Cdr1-C1106 is a key residue for the structural integrity of Cdr1.

Confocal microscopy images of Cdr1, Cdr1PC-NTS12, and Cdr1PC-NTS12-S1 (Fig. 5) showed how substituting S1106 with I1106 restored proper plasma membrane localization of Cdr1PC-NTS12-S1. This almost Cys-less, but functional, Cdr1 variant was renamed Cdr1PC-CID. Removal of the GFP tag confirmed that the GFP tag did not interfere with the FLC efflux pump function of Cdr1PC-CID. The MIC_FLC_ of cells overexpressing Cdr1PC-CID with or without a GFP tag was 128 mg/liter.

Cdr1-C1106 and -P1112 and the ABCG1 NPADF motif are both part of the newly discovered GX_2[3]_CPX_3_NPXD/E motif which has been noted as an important contact point between the two NBDs at the cytosolic apex of human ABCG5/G8 (18). Recent structures of the human multidrug efflux pump ABCG2 in both the open (20) and closed (19) conformation, and the previous ABCG5/G8 structure, provided important insights into how ABCG transporters work. The authors characterized a substrate binding cavity between TMS2 and TMS5 (19). In addition, they proposed (i) a rigid-body motion of the two TMDs and the two NBDs with the coupling helices acting as pivot points and (ii) that ABCG2 hydrolyzes two ATP molecules per transport cycle (20). A comparison between the two ABCG2 conformations (19) reveals the two NPAD helices as a possible third important pivot point around which the NBDs rotate at the peripheral apex of the converging NBDs. Interestingly, the human cholesterol transporter ABCG5/G8 is an asymmetric transporter that hydrolyzes only one ATP molecule per transport cycle at the catalytic G5-NBD (40, 41). ABCG8 has a noncanonical Walker A motif, and ABCG5 has a noncanonical ABC signature motif. Although the G8-NBD did not contribute to ATP hydrolysis, it was essential for normal cholesterol transport (40). The vast evolutionary distances between ABCG2, ABCG5, ABCG8, ABCG1, ABCG4, and plant and fungal half and full-size ABCG transporters (see Fig. S4 in the supplemental material and the work of Lee et al., 2016 [18]) would certainly allow for the evolution of different mechanisms of ATP hydrolysis and/or transport of various ABCG lineages. What is conserved, however, is the GX_2[3]_CPX_3_NPXD/E motif that appears to be an important pivot point at the cytosolic apex of all ABCG transporters. Various ABCG families may have evolved alternative degenerate GX_2[3]_CPX_3_NPXD/E motifs to accommodate their differences in ATP hydrolysis and/or transport.

### Conclusion.

In an attempt to create a Cys-less, but functional, Cdr1 molecule, a number of important residues (G521 of TMS1, V693 of PDRB, C1106, part of the CP2 motif, and A1207 of TMS2) were discovered as potentially critical contact points in the rigid-body motions of Cdr1 during its transport cycle. We have also discovered a novel ABCG transporter-specific loop-helix motif just underneath, and perpendicular to, the ATP-binding pockets of Cdr1. The canonical NBD2-GX_3_CPX_3_NPAE motif and possibly also the degenerate NBD1-GX_2_CPX_3_TTAD motif are in close contact with both H-loops. The six extracellular cysteines are critically important for the structural integrity of Cdr1. Conservation of these residues suggests a similar function in all fungal PDR transporters. As a result of our investigations, we were able to create an “almost Cys-less” Cdr1 molecule that is functional and can be used for cysteine-cross-linking studies of the prototype fungal PDR transporter, C. albicans Cdr1. The creation of this mutant opens new avenues for studying ATP hydrolysis and substrate translocation of fungal PDR transporters in greater detail. It also provides an indispensable tool for the verification of any future structure(s) solved for this important family of efflux transporters.

## MATERIALS AND METHODS

### Strains and culture conditions.

The yeast strains and plasmids used in this study are listed in Tables 4 and 5, respectively. S. cerevisiae strains were cultured in yeast extract-peptone-dextrose (YPD) medium: 1% Bacto yeast extract (Difco Laboratories, Detroit, MI), 2% Bacto peptone (Difco), 2% glucose. Uracil prototroph transformants were selected on complete supplement mixture without uracil (CSM-URA) dropout agar plates: 0.077% CSM-URA (Bio 101, Vista, CA), 0.67% yeast nitrogen base without amino acids (Difco), 2% glucose, 2% agar (Difco).

**TABLE 4:** S. cerevisiae strains used in this study

Strains^*a*^	Genotype/description	Reference
ADΔ	*MAT*α *his1* Δ*ura3*::*dpl200 PDR1-3* Δ*yor1*::*hisG* Δ*ycf1*::*hisG* Δ*snq2*::*hisG* Δ*pdr3*::*hisG* Δ*pdr5*::*hisG* Δ*pdr10*::*hisG* Δ*pdr11*::*hisG* Δ*pdr15*::*hisG*	51
ADΔΔ	ADΔ, Δ*his1*::*dpl200*	52
ADΔΔ-pABC3	ADΔΔ, *Δpdr5*::*pABC3*	This study
ADΔΔ-CaCDR1P	ADΔΔ, *Δpdr5*::*CaCDR1P*	This study
ADΔΔ-CaCDR1P-GFP	ADΔΔ, *Δpdr5*::*CaCDR1P-GFP*	This study
ADΔΔ-CaCDR1PC	ADΔΔ, *Δpdr5*::*CaCDR1PC*	This study
ADΔΔ-CaCDR1PC-GFP	ADΔΔ, *Δpdr5*::*CaCDR1PC-GFP*	This study
-N1	ADΔΔ, *Δpdr5*::*CaCDR1PC-N1-GFP*	This study
-TS1	ADΔΔ, *Δpdr5*::*CaCDR1PC-TS1-GFP*	This study
-EL3	ADΔΔ, *Δpdr5*::*CaCDR1PC-EL3-GFP*	This study
-T1	ADΔΔ, *Δpdr5*::*CaCDR1PC-T1-GFP*	This study
-NTS1	ADΔΔ, *Δpdr5*::*CaCDR1PC-NTS1-GFP*	This study
-NT1	ADΔΔ, *Δpdr5*::*CaCDR1PC-NT1-GFP*	This study
-NT1-S1	ADΔΔ, *Δpdr5*::*CaCDR1PC-NT1-S1-GFP*	This study
-NT1-S2	ADΔΔ, *Δpdr5*::*CaCDR1PC-NT1-S2-GFP*	This study
-NT1-S3	ADΔΔ, *Δpdr5*::*CaCDR1PC-NT1-S3-GFP*	This study
-NT1-S1-N2	ADΔΔ, *Δpdr5*::*CaCDR1PC-NT1-S1-N2-GFP*	This study
-N2	ADΔΔ, *Δpdr5*::*CaCDR1PC-N2-GFP*	This study
-TS2	ADΔΔ, *Δpdr5*::*CaCDR1PC-TS2-GFP*	This study
-EL6	ADΔΔ, *Δpdr5*::*CaCDR1PC-EL6-GFP*	This study
-T2	ADΔΔ, *Δpdr5*::*CaCDR1PC-T2-GFP*	This study
-NTS2	ADΔΔ, *Δpdr5*::*CaCDR1PC-NTS2-GFP*	This study
-NT2	ADΔΔ, *Δpdr5*::*CaCDR1PC-NT2-GFP*	This study
-N12	ADΔΔ, *Δpdr5*::*CaCDR1PC-N12-GFP*	This study
-TS12	ADΔΔ, *Δpdr5*::*CaCDR1PC-TS12-GFP*	This study
-EL36	ADΔΔ, *Δpdr5*::*CaCDR1PC-EL36-GFP*	This study
-T12	ADΔΔ, *Δpdr5*::*CaCDR1PC-T12-GFP*	This study
-NTS12	ADΔΔ, *Δpdr5*::*CaCDR1PC-NTS12-GFP*	This study
-NTS12-S1^*b*^	ADΔΔ, *Δpdr5*::*CaCDR1PC-NTS12-S1-GFP*	This study
-CID	ADΔΔ, *Δpdr5*::*CaCDR1PC-NTS12-S1*	This study

a N1, TS1, EL3, T1, N2, TS2, EL6, T2, etc., denote ADΔΔ strains overexpressing *CDR1P* mutants whose cysteines of the indicated subdomains have been replaced with serine, alanine, or isoleucine (Tables 1 and 3 give further details).

b This strain was renamed ADΔΔ-CaCDR1P-CID-GFP.

**TABLE 5:** Plasmids used in this study

Plasmids	Genotype/description	Reference
pABC3-CaCDR1P^*a*^	C. albicans *CDR1P* cloned into plasmid pABC3	This study
pABC3-CaCDR1PC^*a*^	Cys-less C. albicans *CDR1PC* cloned into pABC3	This study
pABC3-CaCDR1P-GFP^*a*^	*CDR1P-GFP* cloned into pABC3	This study
pABC3-CaCDR1PC-GFP^*a*^	*CDR1PC*-*GFP* cloned into pABC3	This study

a These plasmids contain the wild-type (P) (6) or the Cys-less (PC) (30) C. albicans *CDR1* alleles cloned into the PacI/NotI restriction sites of either plasmid pABC3 or pABC3-GFP (51).

### PCR.

PCR was used to amplify DNA fragments. A 50-μl PCR mixture contained 20 to 50 ng genomic DNA (gDNA) or 1 to 10 ng plasmid DNA template, 5 μl 10× Phusion Hot Start Flex (HF) reaction buffer, 1 μl 10 mM deoxynucleoside triphosphates (dNTPs) (New England Biolabs, Ipswich, MA), 8 μl DNA oligonucleotide primers (3.2 μM), and 0.5 μl (1 U) high-fidelity Phusion HF DNA polymerase (New England Biolabs). A list of primers (Sigma-Aldrich NZ Ltd., Auckland, New Zealand) used in this study is provided in Table S1 in the supplemental material. Routine PCRs were performed with the following cycling protocol: denaturation at 98°C for 30 s, followed by 25 cycles (for plasmid templates) or 35 cycles (for gDNA templates) of 10 s denaturation at 98°C, 10 s annealing at 65°C, and 15- to 30-s/kb extension of DNA fragments at 72°C, and a final 5-min step at 72°C.

10.1128/mSphere.01318-20.8 TABLE S1 DNA oligonucleotide primers used in this study. Download Table S1, DOCX file, 0.02 MB. Copyright © 2021 Madani et al. 2021 Madani et al. https://creativecommons.org/licenses/by/4.0/ This content is distributed under the terms of the Creative Commons Attribution 4.0 International license.

10.1128/mSphere.01318-20.9 TABLE S2 Mass spectrometry analysis of the ∼70-kDa protein band upregulated in N2 subdomain containing ADΔΔ-CaCDR1PC-GFP variants. The ∼70-kDa band was identified as S. cerevisiae heat shock protein Ssa2 (score: 2,156; database: Swiss-Prot). The identified peptides (bold) covered 43% of the protein. Download Table S2, DOCX file, 0.02 MB. Copyright © 2021 Madani et al. 2021 Madani et al. https://creativecommons.org/licenses/by/4.0/ This content is distributed under the terms of the Creative Commons Attribution 4.0 International license.

KOD Fx Neo (Toyobo Co. Ltd., Osaka, Japan) was used to confirm correct yeast transformants by PCR amplification of the 8-kb transformation cassette. The 10-μl PCR mixture contained 5 μl 2× PCR buffer, 2 μl dNTPs (2 mM), 0.8 μl DNA oligonucleotide primers (3.2 μM), 0.2 μl KOD Fx Neo DNA polymerase (1 U/μl), and 1.2 μl cell suspension of transformants picked directly from CSM-URA agar plates. Colony PCR was performed by denaturation at 98°C for 30 s, followed by 45 cycles of 10 s denaturation at 98°C, 10 s annealing at 65°C, and 1-min/kb extension of DNA fragments at 68°C. A final step was performed at 68°C for 5 min.

### Yeast transformation.

Transformation-competent S. cerevisiae ADΔΔ or ADΔ cells, prepared using adaptations of a previous protocol (42), were used fresh or stored at −80°C. Fresh cells, or frozen competent cells defrosted for 5 min in a 30°C water bath, were aliquoted (50 μl) into 1.5-ml microcentrifuge tubes, harvested by centrifugation (1 min, 18,000 × *g*), and kept on ice until transformed. Salmon sperm carrier DNA (2 mg/ml) was denatured for 10 min in a boiling water bath and kept on ice. Fifty-microliter aliquots of the salmon sperm DNA were mixed with 14 μl (0.5 to 2 μg) DNA containing equimolar amounts of the transforming PCR fragments and kept on ice. For each transformation, 260 μl 50% (wt/vol) polyethylene glycol (PEG 3350) and 36 μl 1 M lithium acetate (LiAc) were mixed, by repeat pipetting, with the 64-μl ice-cold DNA mixtures. This PEG-LiAc-DNA mixture was then used to resuspend the ice-cold competent cell pellets by thorough vortexing for 10 s. The cell suspension was incubated in a 30°C water bath for 1 h. The transformed cells were harvested by centrifugation (18,000 × *g*, 10 s), the supernatant was discarded, and the cell pellet was resuspended in 80 μl double-distilled water (ddH_2_O) and spread onto CSM-URA agar plates. The plates were incubated at 30°C for 2 to 3 days until uracil prototroph transformants were clearly visible. To confirm that uracil prototroph transformants contained the desired DNA elements correctly inserted at the chromosomal *PDR5* locus, colony PCR was performed with the primer pair PDR5-up/PDR5-down, and the correct ∼8-kb Ca*CDR1* transformation cassettes were confirmed by sequencing the entire *CDR1* ORF.

### Construction of yeast strains expressing Cdr1-GFP variants.

The yeast strain expressing Cys-less *CDR1PC*, ADΔΔ-CaCDR1PC-GFP (P indicates the wild-type Cdr1 allele that Prasad’s research team used to create the “Cys-less” *CDR1* mutant [30] and C indicates “Cys-less”) had to be first corrected for eight additional mutations, six of which caused amino acid changes, in the original construct kindly provided by R. Prasad. ADΔΔ strains expressing the various *CDR1* mutants, in a genetic background where 7 endogenous efflux pumps have been deleted, were created with a one-step transformation protocol (43, 44) of up to five individual PCR fragments each overlapping by 25 bp. An illustration of this one-step cloning strategy is provided in Fig. S6. The individual DNA fragments were PCR amplified from various DNA templates (i.e., plasmids pABC3-CaCDR1P-GFP and pABC3-CaCDR1PC-GFP or from gDNA extracts of yeast strains expressing the necessary portions of *CDR1*). The ADΔΔ strain is prone to acquiring petite mutations. To ensure that the transformants were not petite mutants, all transformants were tested for growth on YPG agar plates: 1% Bacto yeast extract (Difco Laboratories, Detroit, MI), 2% Bacto peptone (Difco), 2% glycerol. Petite mutants cannot grow on nonfermentable carbon sources such as glycerol.

10.1128/mSphere.01318-20.2 FIG S6 Direct one-step multifragment cloning strategy to create ADΔΔ cells overexpressing *CDR1PC*-*GFP* variants. Up to four individual DNA fragments of the various *CDR1* transformation cassettes that overlapped by 25 bp were amplified by PCR with the indicated forward (pro [pPDR5-pro] and P1- to P3-for) and reverse (ter [pPDR5-ter] and P1- to P3-rev) primers to amplify the indicated PCR fragments. The *PDR5* promoter and downstream sequences, the gene of interest, the *PGK1* terminator, and the *URA3* selection marker are in blue, brown, green, and light blue, respectively. The homologous crossover events (2, 3, 4, or 5) that were necessary to integrate the entire transformation cassette in one piece (top), or 2, 3 or 4 pieces (underneath), into the genomic *PDR5* locus are indicated with gray crosses. The yellow lines depict desired mutations that were introduced by primer design of the indicated *CDR1*-specific -for and -rev primer pairs. Correct transformants were verified by colony PCR with a primer pair (up [pPDR5-up] and down [pPDR5-down]) that binds ∼40 bp upstream and downstream of the chromosomal integration site, respectively. Download FIG S6, TIFF file, 1.1 MB. Copyright © 2021 Madani et al. 2021 Madani et al. https://creativecommons.org/licenses/by/4.0/ This content is distributed under the terms of the Creative Commons Attribution 4.0 International license.

### Isolation of *CDR1PC-*NT1 and -NTS12 suppressor mutations.

Naturally arising ADΔΔ-CaCDR1PC-NT1-GFP or -NTS12-GFP suppressor mutants with reduced FLC efflux pump function were selected by plating 10^6^ to 10^7^ logarithmic-phase cells on CSM agar plates supplemented with high concentrations of FLC (∼4× to 8× MIC_FLC_) and incubating the plates for up to 2 weeks at 30°C, as previously described (12, 24). Individual colonies were tested for possible mutations by sequencing the entire *CDR1* ORF.

### Determination of azole susceptibilities.

The minimum growth-inhibitory concentration (MIC) assay (45) was used as a proxy to measure the functionality of the various *CDR1* constructs. FLC (Diflucan; Pfizer Laboratories, Auckland, New Zealand), CLT (Bayer, Osaka, Japan), KTC, ANI, CHX, R6G, and nigericin (NIG; Sigma-Aldrich New Zealand, Auckland, New Zealand) were used as xenobiotic test compounds. Twofold serial dilutions of FLC (0.5 to 512 mg/liter), ANI (0.016 to 16 mg/liter), CHX (0.004 to 4 mg/liter), CLT (0.002 to 2 mg/liter), R6G (0.5 to 64 mg/liter), KTC (0.004 to 4 mg/liter), and NIG (0.063 to 64 mg/liter) were prepared in 100 μl CSM (pH 7.0) in the wells of 96-well microtiter plates. A logarithmic-phase cell culture was diluted with fresh CSM (pH 7.0) to an optical density at 600 nm (OD_600_) of 0.01, and 100 μl of the cell suspension (∼10,000 cells) was added to the diluted drugs in individual microtiter plate wells. A no-drug positive growth control was also included. Cell growth was monitored after incubation at 30°C for 48 h. MIC_FLC_s for yeast strains were routinely determined as technical duplicates repeated twice for three separate transformants. Positive (ADΔΔ-CaCDR1P-GFP) and negative (ADΔΔ-pABC3) controls were included in each experiment.

### An optimized small-scale yeast plasma membrane preparation protocol.

A single yeast colony from a YPD-agar plate was used to inoculate 10 ml YPD medium and incubated at 30°C with shaking at 200 rpm for ∼7 to 8 h. This preculture was used to inoculate 40 ml YPD medium which was incubated at 30°C with shaking at 200 rpm for ∼16 h until the culture reached an OD_600_ of 1 to 3 (i.e., ∼1 × 10^7^ to 3 × 10^7^ cells/ml). Forty OD units (ODU; e.g., 1 ml at an OD_600_ of 1 = 1 ODU) of these cells were harvested by centrifugation (4,200 × *g*, 5 min, 4°C) and washed once with 40 ml ice-cold ddH_2_O. The cell pellet was resuspended in 1 ml ice-cold ddH_2_O, transferred to a 1.5-ml microcentrifuge tube, and placed on ice. Cells were harvested by centrifugation (18,000 × *g*, 3 min, 4°C) and resuspended in 0.5 ml homogenizing buffer (HB; 50 mM Tris-Cl, pH 7.5; 0.5 mM EDTA; 20% [vol/vol] glycerol freshly supplemented with 1 mM phenylmethylsulfonyl fluoride [PMSF]). Cell suspensions were stored at −80°C for future use or kept on ice for immediate breakage. Approximately 1 g ice-cold 0.5-mm-diameter silica beads was added to the 0.5-ml cell suspension (frozen cells were thawed on ice for ∼1 h) to reach a total volume of ∼1 ml. The cells were broken by 6 cycles of vortexing at maximum shaking intensity for 1 min and 3-min cooling periods on ice. The broken cell homogenate was transferred to a new 1.5-ml microcentrifuge tube by making a hole with a hot needle in the bottom of the tube. The tube was placed into a new open 1.5-ml microcentrifuge tube, and the cell homogenate was collected through the hole into the lower microcentrifuge tube with a 10-s low-speed (∼200-rpm) spin; the silica beads remained in the original tube. The 0.5-ml cell homogenate was centrifuged (5,156 × *g*, 5 min, 4°C) to remove cell debris, unbroken cells, and nuclei. Most of the supernatant (450 μl) was transferred into an ice-cold 1.5-ml microcentrifuge tube, and 1 ml ice-cold HB freshly supplemented with 1 mM PMSF was added. The crude plasma membranes were harvested by centrifugation (18,000 × *g*, 1 h, 4°C) and resuspended in 100 μl ice-cold HB (freshly supplemented with 1 mM PMSF).

### Cdr1 ATPase activities.

The Cdr1 ATPase activities of the crude plasma membrane preparations were determined in triplicate in the absence or presence of 20 μM Cdr1-ATPase inhibitor oligomycin (Sigma-Aldrich). The ATPase assay cocktail (75 mM morpholineethanesulfonic acid [MES]-Tris, pH 7.5, 75 mM potassium nitrate, 0.3 mM ammonium molybdate, 7.5 mM sodium azide) and 28.8 mM Mg-ATP (pH 7.0) were equilibrated to 30°C for ∼1 h before the ATPase assay was undertaken. Aliquots of the prewarmed ATPase assay cocktail (90 μl), with or without inhibitor, were distributed into wells of a 96-well microtiter plate, and portions (5 μl) of crude plasma membrane preparations containing ∼5 to 10 μg of crude plasma membrane protein, or 5 μl of phosphate standards (0 to 100 nmol P_i_), were added to the appropriate wells. The assay was started by adding 25 μl prewarmed 28.8 mM Mg-ATP to each well and incubating the reaction mixture at 30°C for 30 min. The ATPase assay was stopped by adding 130 μl 1.6% sodium l-ascorbate, 1% SDS, 12% ammonium molybdate in 6 M sulfuric acid to each well, and the dye was left to develop for 10 min at room temperature (RT) before the *A*_750_ was measured with a microtiter plate reader (Synergy 2 Multi-Detection; BioTek Instruments, Inc., Winooski, VT). The total and oligomycin-sensitive (OS; 20 μM oligomycin) ATPase activities (nmol/min/mg) of plasma membrane preparations were determined, and the OS ATPase activities were corrected by subtracting the background OS ATPase activity of the negative-control strain, ADΔΔ, to obtain the Cdr1-specific ATPase activities.

### Quantification of Cdr1 expression levels.

Expression levels of C-terminally GFP-tagged Cdr1 variants were quantified after separation of plasma membrane proteins (10 or 20 μg) by SDS-PAGE and quantifying the in-gel green fluorescence intensities emitted by Cdr1-GFP with the Bio-Rad Gel-Doc system (Gel-Doc EZ Imager; Bio-Rad, Hercules, CA).

### Confocal microscopy of yeast cells expressing Cdr1-GFP variants.

CSM medium (2 ml) was inoculated with a single yeast colony and incubated at 30°C for 16 h with shaking (200 rpm). A portion of the culture was used to inoculate 1 ml CSM to an OD_600_ of ∼0.5, and the culture was incubated at 30°C with shaking (200 rpm) for another ∼2 to 4 h to reach an OD_600_ of 1. The cells were harvested by centrifugation (18,000 × *g*, 1 min), resuspended in ∼0.1 ml ice-cold CSM (OD_600_ = 10), and kept on ice. Ten microliters of the ice-cold cell suspensions was transferred onto agarose-coated glass slides, to avoid movement of the cells, and covered with a cover slip. A thin agarose layer was created on the slide by placing 1 to 2 ml molten agarose (0.7% [wt/vol]) onto the slide and letting it dry for ∼1 h in a laminar flow cabinet. Images of cells and their GFP signals were obtained with a Zeiss LSM 710 confocal laser scanning microscope at ×630 magnification using a 488-nm argon laser for excitation of the GFP fluorophore. GFP fluorescence was detected at an emission wavelength window of 495 to 598 nm.

### Analysis of phylogenetic relationships and sequence alignments of conserved NBD regions.

Phylemon 2.0 (46), a publicly available web tool, was used for sequence alignments and tree reconstruction. Sequence alignments were performed with ClustalW v2.0.10 (47) and viewed and edited with Jalview14.0 (48). FigTree v1.4.0 (http://tree.bio.ed.ac.uk/software/figtree/) was used to produce publication-ready figures. Phylogenetic relationships were calculated by maximum likelihood analysis using PhyML v3.0 (49, 50).

10.1128/mSphere.01318-20.1 TEXT S1 FASTA file of select representative half and full-size ABCG transporters from plants and fungi including YOL075C orthologs of various fungi. Download Text S1, DOCX file, 0.06 MB. Copyright © 2021 Madani et al. 2021 Madani et al. https://creativecommons.org/licenses/by/4.0/ This content is distributed under the terms of the Creative Commons Attribution 4.0 International license.
