# Supplementary figures and images for: Engineering a Cysteine-Deficient Functional Candida albicans Cdr1 Molecule Reveals a Conserved Region at the Cytosolic Apex of ABCG Transporters Important for Correct Folding and Trafficking of Cdr1
Source: mSphere. 2021 Feb 10;6(1):e01318-20. doi: 10.1128/mSphere.01318-20 (PMC8544900; doi:10.1128/mSphere.01318-20)

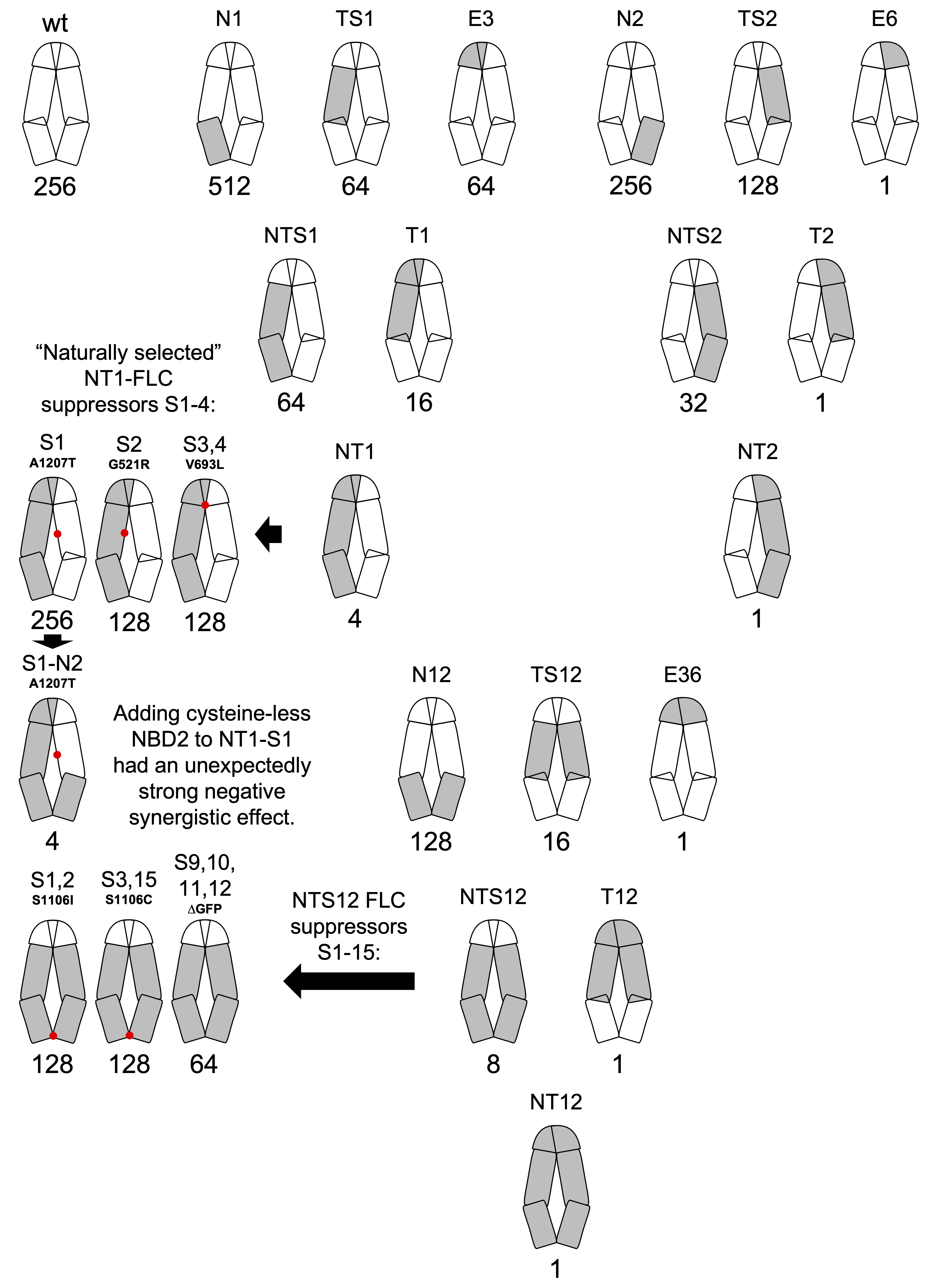

Supplement: FIG S1 [file msphere.01318-20-sf001.tiff]

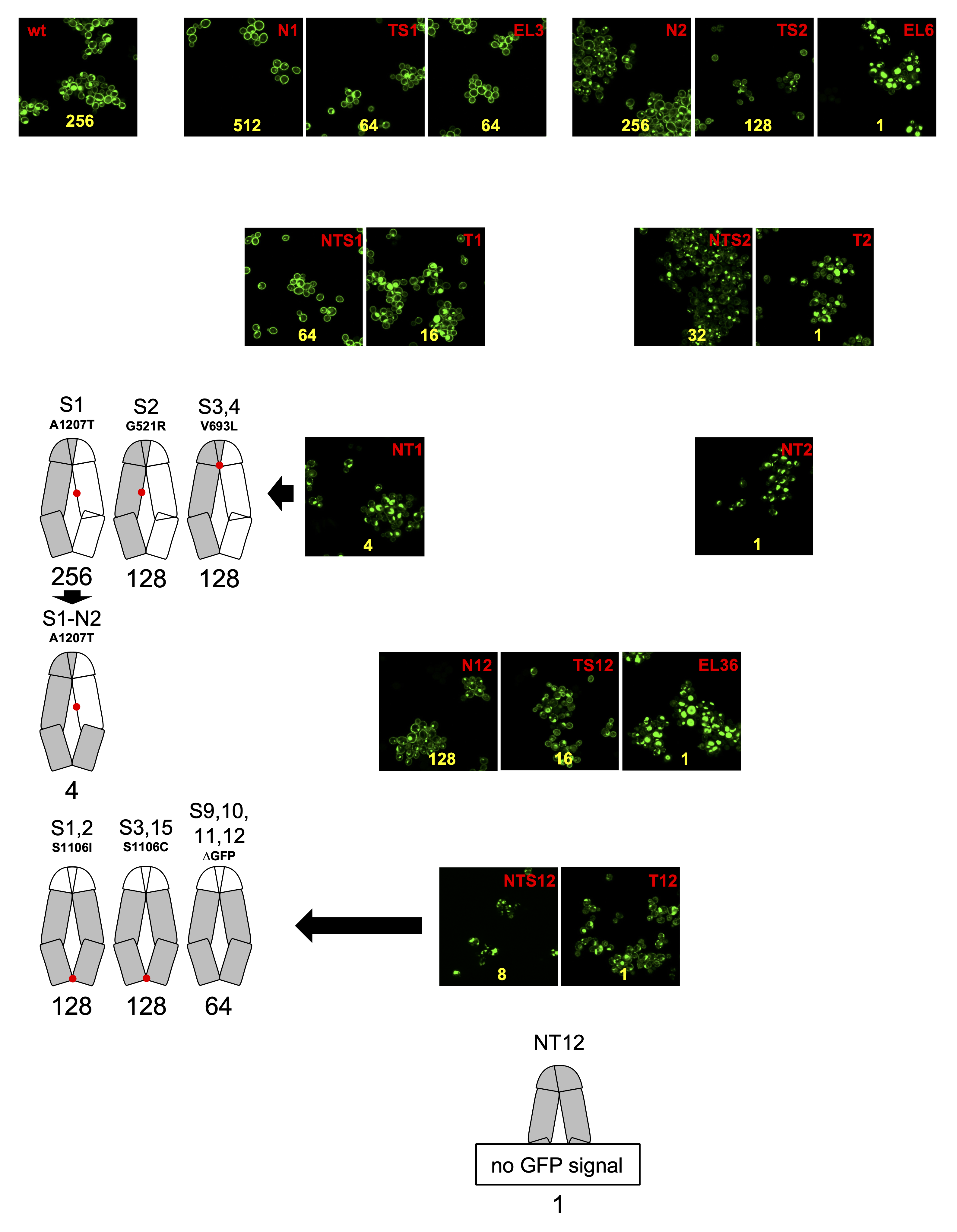

Supplement: FIG S2 [file msphere.01318-20-sf002.tiff]

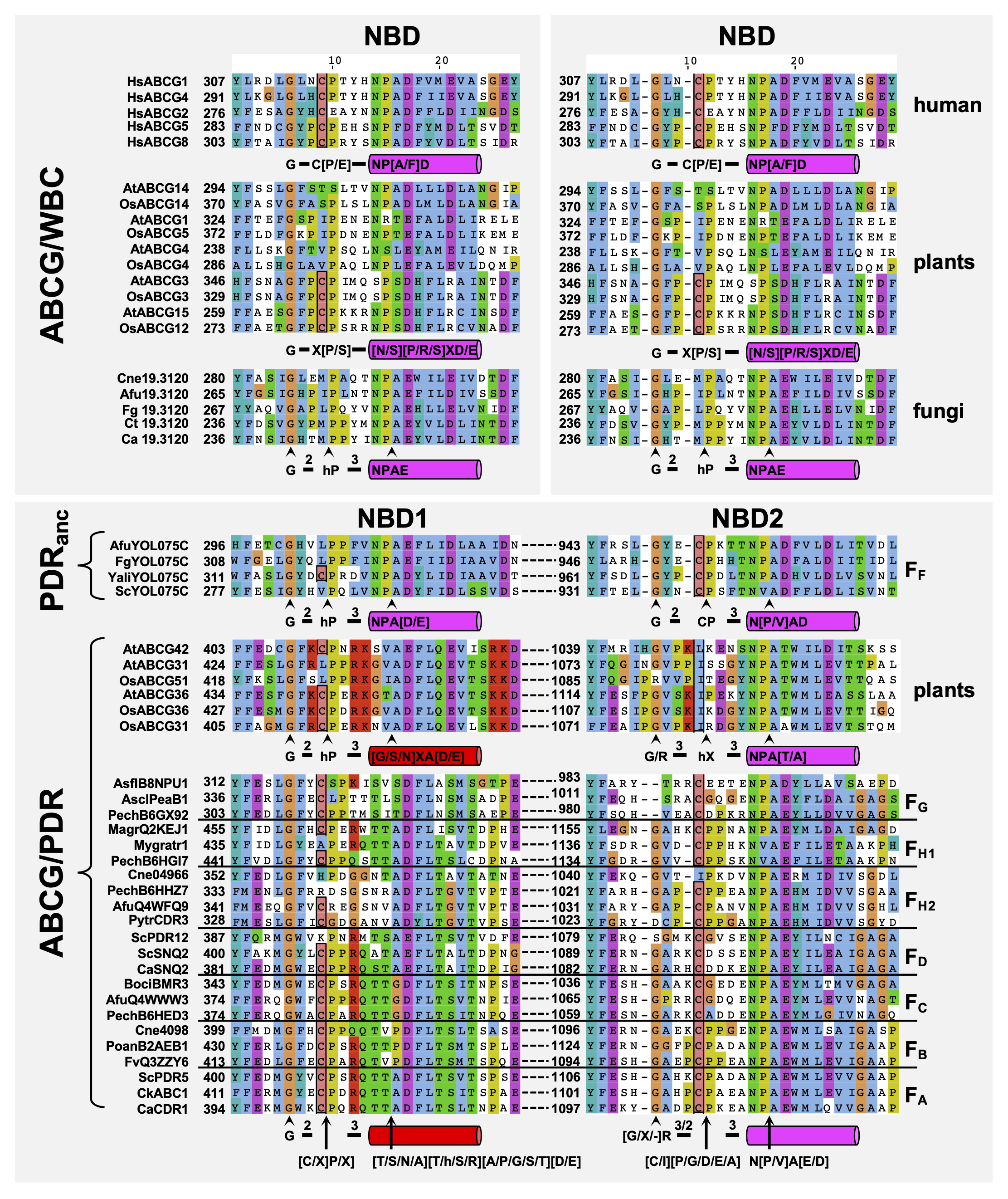

Supplement: FIG S3 [file msphere.01318-20-sf003.tiff]

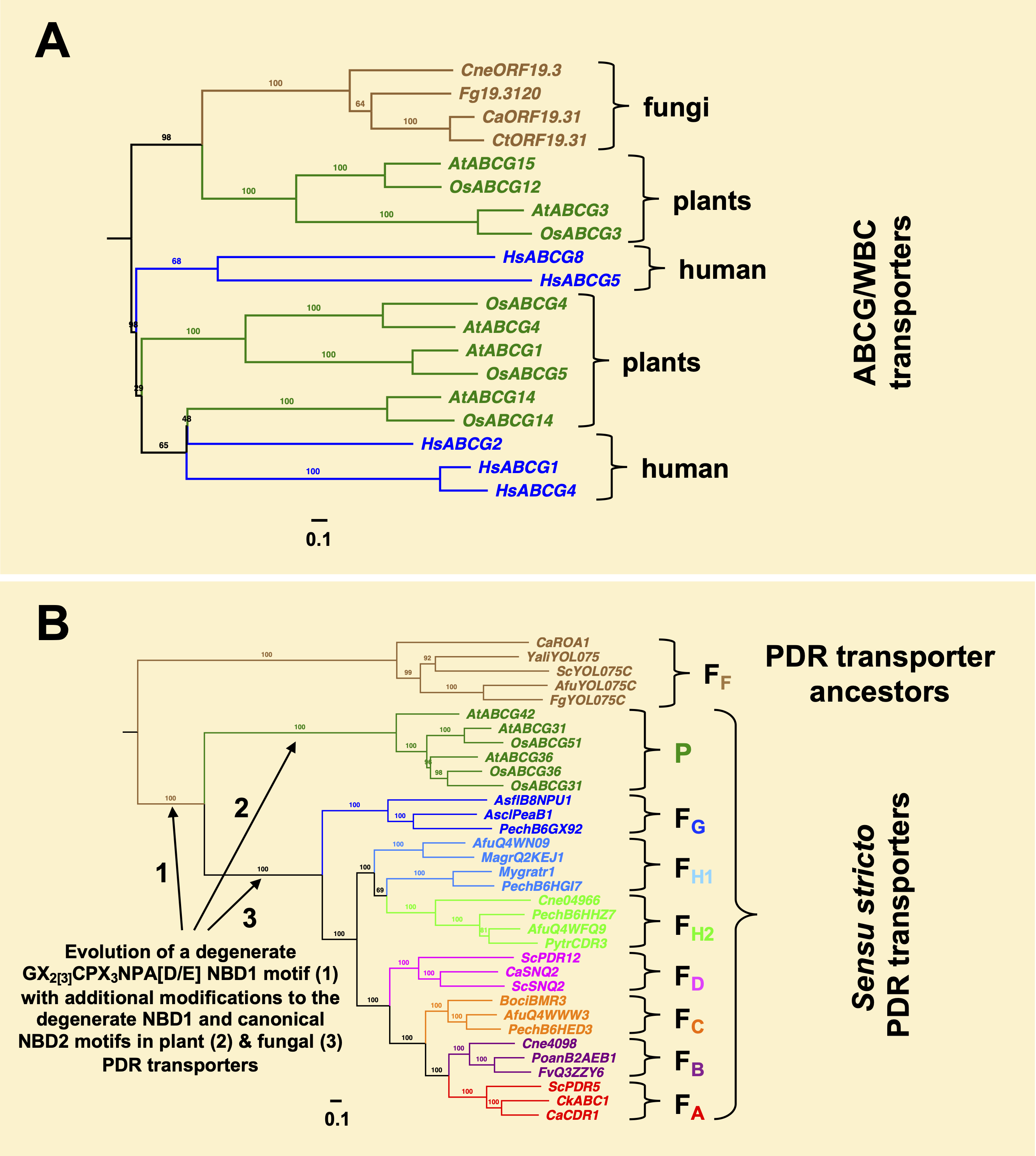

Supplement: FIG S4 [file msphere.01318-20-sf004.tiff]

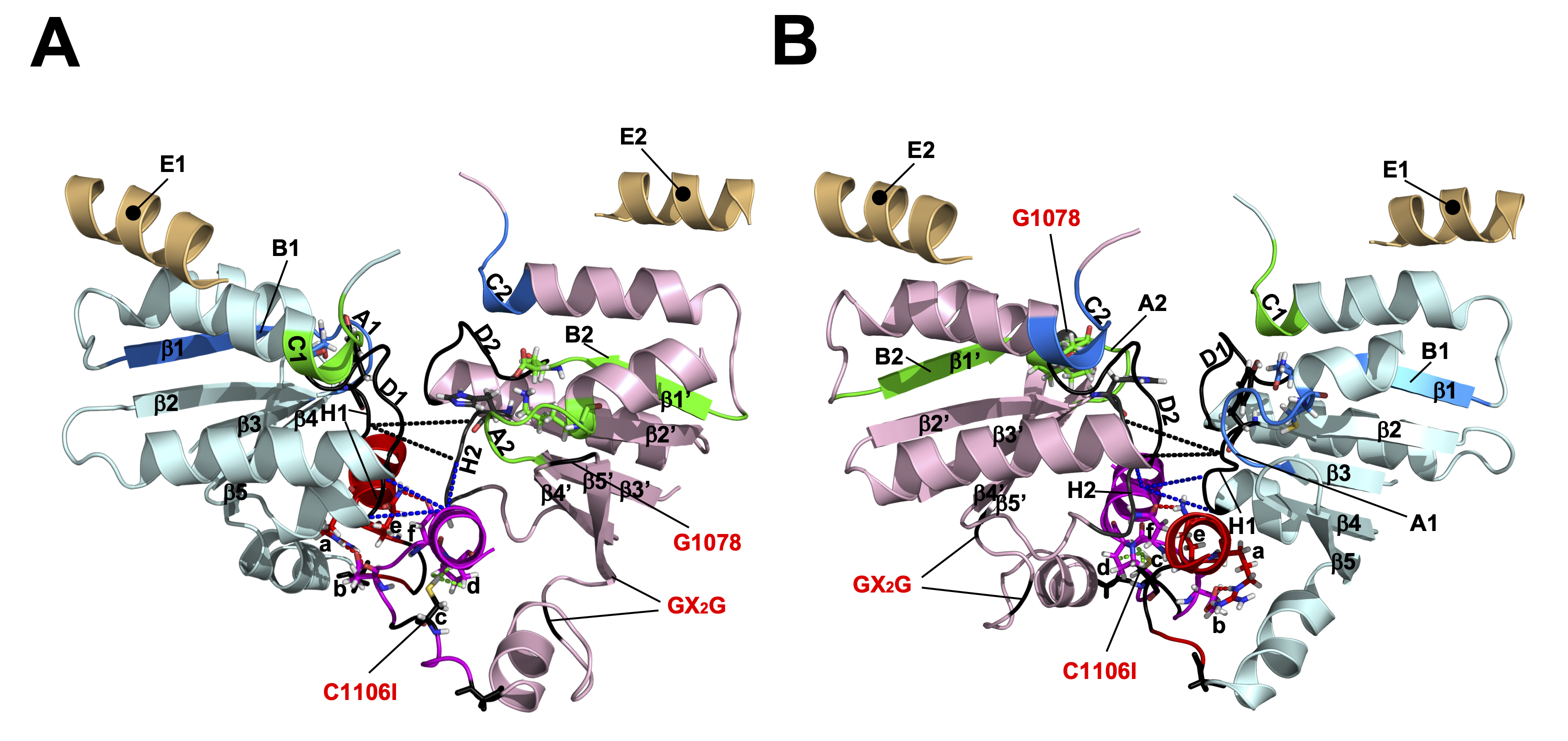

Supplement: FIG S5 [file msphere.01318-20-sf005.tiff]

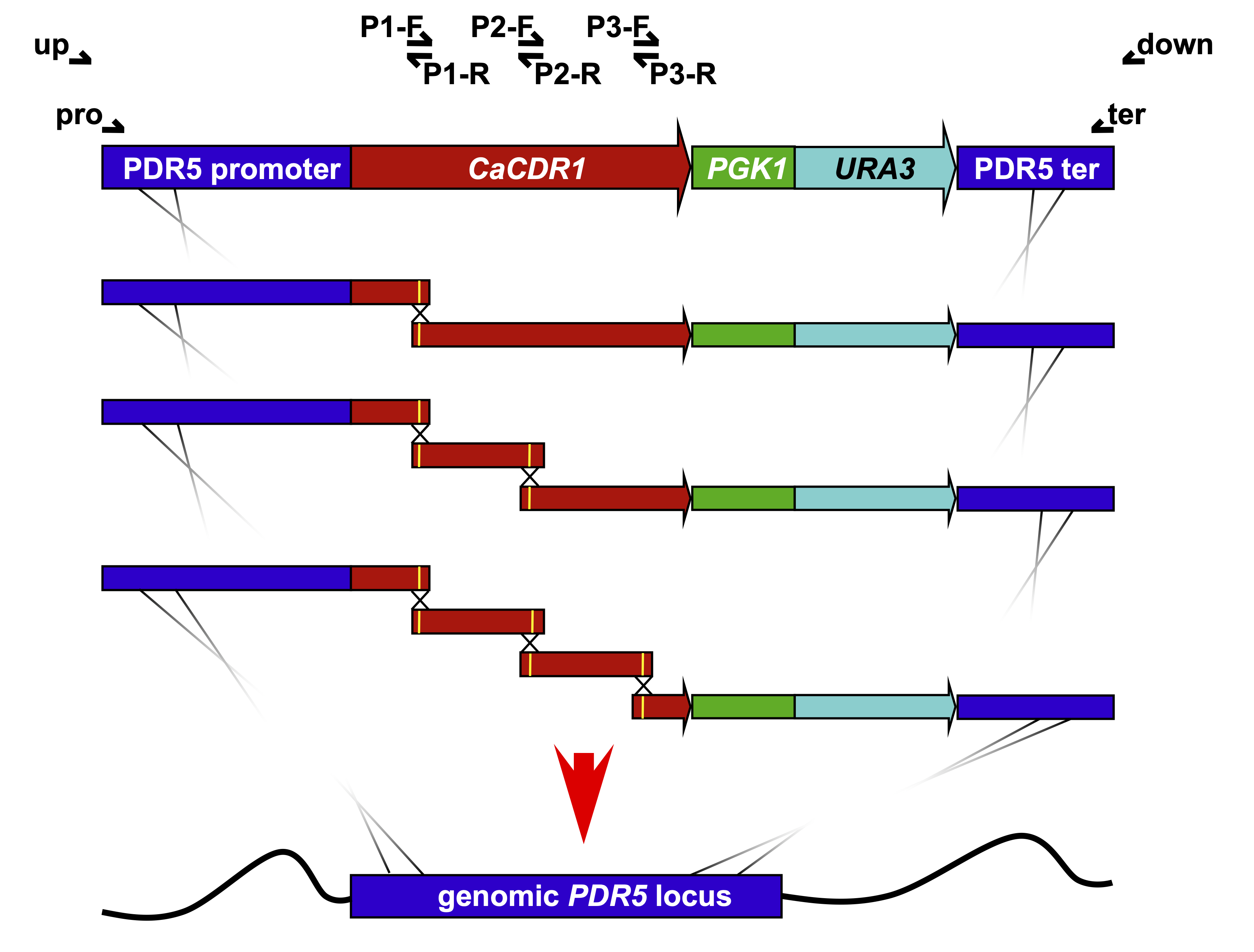

Supplement: FIG S6 [file msphere.01318-20-sf006.tiff]
