# Supplementary material for: Engineering a Cysteine-Deficient Functional Candida albicans Cdr1 Molecule Reveals a Conserved Region at the Cytosolic Apex of ABCG Transporters Important for Correct Folding and Trafficking of Cdr1
Source: mSphere. 2021 Feb 10;6(1):e01318-20. doi: 10.1128/mSphere.01318-20 (PMC8544900; doi:10.1128/mSphere.01318-20)
Supplement: TABLE S1 [file msphere.01318-20-st001.docx]

Supplementary Table S1. DNA oligonucleotide primers used in this study.

| **Purpose** | **Name** | **Sequence (5’ -> 3’)** |
| --- | --- | --- |
| Cloning | pCDR1-C1380A-for | TTCATGTACAGAGCTAATCCATTCAC |
|  | pCDR1-C1380A-rev | GTGAATGGATTAGCTCTGTACATGAA |
|  | pCDR1-1448-for | CAAGACCAGCATCTCCATATACTG |
|  | pCDR1-1448-rev | CAGTATATGGAGATGCTGGTCTTG |
|  | pCDR1-2014-for | GGTTCGTTATCCCAACTCCAAGT |
|  | pCDR1-2014-rev | ACTTGGAGTTGGGATAACGAACCC |
|  | pCDR1-4093-for | GCTGGTCCTGATGTATTACCAGGA |
|  | pCDR1-4093-rev | TCCTGGTAATACATCAGGACCAGC |
|  | pCDR1-2480-for | GAAGCTGGTCCTGTTGCTGGCAAAC |
|  | pCDR1-2480-rev | GTTTGCCAGCAACAGGACCAGCTTC |
|  | pCDR1-3500-for | TACCAAGAGATAATGATCCAGAAGC |
|  | pCDR1-3500-rev | GCTTCTGGATCATTATCTCTTGGTA |
|  | pCDR1-∆EL3 | GGGTTCGTTATCCCAACTCCAAGTAATTCTCATAAATGGAGAAACTTGGGT |
|  | pCDR1-∆EL6 | GCTGGTCCTGATGTATTACCAGGAAGTGAAAGATGGAGAAATTTCGGT |
| Confirmation of newly developed strains | pPDR5-up | GCATAAAACAGAGAGGCGATATAGG |
|  | pPDR5-down | AGAAGACGGTTCGCCATTCGGACAG |
|  | pPDR5-pro | ATCATACAAAGGAAGCGTTCTCTGGG |
|  | PRO-GOL | TCCTCAGCGCGAACGTTCGCATTCT |
|  | pABC3-for | ATAAATTGGCAACTAGGAACTTTCG |
|  | PGK1-rev | TTTCGGATAAGAAAGCAACACCTGG |
|  | pPDR5-ter | TTTAGGCACTCTTGCTAACCAGTAGA |
|  | TER-GOL | CGCTCGTCGTTTGGTATGGCTTCATTC |
| DNA sequencing | pCDR1-593 | AGACCATTGCTGTTAACACTTATGG |
|  | pCDR1-697 | ATTGAACGTCATTACCGTGGTGATG |
|  | pCDR1-1202 | AATGTCCTCAAAGACAAACCACTGC |
|  | pCDR1-1400 | TTGTTGAATGTGAACGATCCAACAC |
|  | pCDR1-1794 | ACCTGTCAAATTAGCCATGTCAATG |
|  | pCDR1-2125 | ATGGTTAATGAATTCCACGGTCGTG |
|  | pCDR1-2385 | TGTTTTGTTCCTCAAAGGGTCGTTG |
|  | pCDR1-2833 | ATTGGTTATGTTCAACAACAAGATG |
|  | pCDR1-2983 | GGTGTTGCTGGTGAAGGTTTGAATG |
|  | pCDR1-3606 | TTTGGTTGTTTCAGCAGCTTTATTC |
|  | pCDR1-4223 | TTTCAGTTAAACCACCAAATGGTGC |
