# Supplementary material for: Engineering a Cysteine-Deficient Functional Candida albicans Cdr1 Molecule Reveals a Conserved Region at the Cytosolic Apex of ABCG Transporters Important for Correct Folding and Trafficking of Cdr1
Source: mSphere. 2021 Feb 10;6(1):e01318-20. doi: 10.1128/mSphere.01318-20 (PMC8544900; doi:10.1128/mSphere.01318-20)
Supplement: TABLE S2 [file msphere.01318-20-st002.docx]

Supplementary Table S2. Mass-spectrometry analysis of the ~70 kDa protein band upregulated in N2 sub-domain containing AD∆∆-CaCDR1PC-GFP variants. The ~70 kDa band was identified as *S. cerevisiae* heat-shock protein Ssa2 (score: 2156; database: SwissProt). The identified peptides (bold) covered 43% of the protein.

| **Heat shock protein Ssa2** | | | | | |
| --- | --- | --- | --- | --- | --- |
| 1 | MSKAVGIDLG | TTYSCVAHFS | NDR**VDIIAND** | **QGNR**TTPSFV | GFTDTERLIG |
| 51 | DAAK**NQAAMN** | **PANTVFDAK**R | LIGR**NFNDPE** | **VQGDMK**HFPF | K**LIDVDGKPQ** |
| 101 | **IQVEFKGETK** | **NFTPEQISSM** | **VLGK**MKETAE | SYLGAK**VNDA** | **VVTVPAYFND** |
| 151 | **SQR**QATK**DAG** | **TIAGLNVLRI** | **INEPTAAAIA** | **YGLDKK**GKEE | HVLIFDLGGG |
| 201 | TFDVSLLSIE | DGIFEVK**ATA** | **GDTHLGGEDF** | **DNRLVNHFIQ** | **EFK**RKNKK**DL** |
| 251 | **STNQR**ALRRL | RTACERAKRT | LSSSAQTSVE | IDSLFEGIDF | YTSITR**ARFE** |
| 301 | **ELCADLFR**ST | LDPVEKVLRD | AK**LDKSQVDE** | **IVLVGGSTR**I | PKVQK**LVTDY** |
| 351 | **FNGKEPNRSI** | **NPDEAVAYGA** | **AVQAAILTGD** | **ESSK**TQDLLL | LDVAPLSLGI |
| 401 | ETAGGVMTKL | IPRNSTIPTK | **KSEVFSTYAD** | **NQPGVLIQVF** | **EGER**AKTKDN |
| 451 | NLLGK**FELSG** | **IPPAPR**GVPQ | IEVTFDVDSN | GILNVSAVEK | GTGKSNKITI |
| 501 | TNDKGRLSKE | DIEKMVAEAE | K**FKEEDEKES** | **QR**IASKNQLE | SIAYSLKNTI |
| 551 | SEAGDKLEQA | DKDAVTKKAE | ETIAWLDSNT | TATKEEFDDQ | LK**ELQEVANP** |
| 601 | **IMSK**LYQAGG | APEGAAPGGF | PGGAPPAPEA | EGPTVEEVD |  |
