# Supplementary material for: Engineering a Cysteine-Deficient Functional Candida albicans Cdr1 Molecule Reveals a Conserved Region at the Cytosolic Apex of ABCG Transporters Important for Correct Folding and Trafficking of Cdr1
Source: mSphere. 2021 Feb 10;6(1):e01318-20. doi: 10.1128/mSphere.01318-20 (PMC8544900; doi:10.1128/mSphere.01318-20)
Supplement: TEXT S1 [file msphere.01318-20-s0001.docx]

**FASTA file of select representative half and full-size ABCG transporters from plants and fungi including YOL075C orthologs of various fungi.**

The list includes representative half and full-size ABCG transporters of a broad range of eukaryotic species. It includes all five human half-size ABCG transporters; plant half- (ABCG/WBC) as well as full-size PDR transporters; and fungal half-size transporters (*Candida albicans* ORF 19.3120 orthologs), symmetric cluster F PDR transporter representatives (i.e. *Saccharomyces cerevisiae* ORF YOL075C orthologs which are the ancestor of all asymmetric PDR transporters) and typical full-size PDR transporters. These transporters were selected because they best represent the depth and breadth of variations seen in the large ABCG superfamily of eukaryotic ABC transporters.

Species abbreviations: Human (Hs); plants: *Arabidopsis thaliana* (At) and *Oryza sativa* (Os); and fungi: the Basidiomycota species *Cryptococcus neoformans* (Cne); Ascomycota of the following Pezizomycotina species of the Dothideomycetes lineage [*Mycosphaerella graminicola* (Mygr), *Pyrenophora tritici-repentis* (Pytr)], the Eurotiomycetes lineage [*Aspergillus fumigatus* (Afu), *Aspergillus flavus* (Asfl), *Aspergillus clavatus* (Ascl), *Penicillium chrysogenum* (Pech)], the Leotiomycetes lineage [*Botrytis cinerea* (Boci)], and the Sordariomycetes lineage [*Magnaporthe grisea* (Magr), *Podospora anserina* (Poan)], two *Fusarium* species [*Fusarium graminearum* (Fg), *Fusarium verticillioides* (Fv)], and five Ascomycota of the following Saccharomycotina species [*C. albicans* (Ca), *Candida krusei* (Ck), *Candida tropicalis* (Ct), *Yarrowia lipolytica* (Yali), *S. cerevisiae* (Sc)].

>HsABCG1 P45844 678 aa

MACLMAAFSVGTAMNASSYSAEMTEPKSVCVSVDEVVSSNMEATETDLLNGHLKKVDNNLTEAQRFSSLPRRAAVNIEFRDLSYSVPEGPWWRKKGYKTLLKGISGKFNSGELVAIMGPSGAGKSTLMNILAGYRETGMKGAVLINGLPRDLRCFRKVSCYIMQDDMLLPHLTVQEAMMVSAHLKLQEKDEGRREMVKEILTALGLLSCANTRTGSLSGGQRKRLAIALELVNNPPVMFFDEPTSGLDSASCFQVVSLMKGLAQGGRSIICTIHQPSAKLFELFDQLYVLSQGQCVYRGKVCNLVPYLRDLGLNCPTYHNPADFVMEVASGEYGDQNSRLVRAVREGMCDSDHKRDLGGDAEVNPFLWHRPSEEVKQTKRLKGLRKDSSSMEGCHSFSASCLTQFCILFKRTFLSIMRDSVLTHLRITSHIGIGLLIGLLYLGIGNEAKKVLSNSGFLFFSMLFLMFAALMPTVLTFPLEMGVFLREHLNYWYSLKAYYLAKTMADVPFQIMFPVAYCSIVYWMTSQPSDAVRFVLFAALGTMTSLVAQSLGLLIGAASTSLQVATFVGPVTAIPVLLFSGFFVSFDTIPTYLQWMSYISYVRYGFEGVILSIYGLDREDLHCDIDETCHFQKSEAILRELDVENAKLYLDFIVLGIFFISLRLIAYFVLRYKIRAER

>HsABCG2 Q9UNQ0 655 aa

MSSSNVEVFIPVSQGNTNGFPATASNDLKAFTEGAVLSFHNICYRVKLKSGFLPCRKPVEKEILSNINGIMKPGLNAILGPTGGGKSSLLDVLAARKDPSGLSGDVLINGAPRPANFKCNSGYVVQDDVVMGTLTVRENLQFSAALRLATTMTNHEKNERINRVIQELGLDKVADSKVGTQFIRGVSGGERKRTSIGMELITDPSILFLDEPTTGLDSSTANAVLLLLKRMSKQGRTIIFSIHQPRYSIFKLFDSLTLLASGRLMFHGPAQEALGYFESAGYHCEAYNNPADFFLDIINGDSTAVALNREEDFKATEIIEPSKQDKPLIEKLAEIYVNSSFYKETKAELHQLSGGEKKKKITVFKEISYTTSFCHQLRWVSKRSFKNLLGNPQASIAQIIVTVVLGLVIGAIYFGLKNDSTGIQNRAGVLFFLTTNQCFSSVSAVELFVVEKKLFIHEYISGYYRVSSYFLGKLLSDLLPMRMLPSIIFTCIVYFMLGLKPKADAFFVMMFTLMMVAYSASSMALAIAAGQSVVSVATLLMTICFVFMMIFSGLLVNLTTIASWLSWLQYFSIPRYGFTALQHNEFLGQNFCPGLNATGNNPCNYATCTGEEYLVKQGIDLSPWGLWKNHVALACMIVIFLTIAYLKLLFLKKYS

>HsABCG4 Q9H172 646 aa 2 EL3 cysteines

MAEKALEAVGCGLGPGAVAMAVTLEDGAEPPVLTTHLKKVENHITEAQRFSHLPKRSAVDIEFVELSYSVREGPCWRKRGYKTLLKCLSGKFCRRELIGIMGPSGAGKSTFMNILAGYRESGMKGQILVNGRPRELRTFRKMSCYIMQDDMLLPHLTVLEAMMVSANLKLSEKQEVKKELVTEILTALGLMSCSHTRTALLSGGQRKRLAIALELVNNPPVMFFDEPTSGLDSASCFQVVSLMKSLAQGGRTIICTIHQPSAKLFEMFDKLYILSQGQCIFKGVVTNLIPYLKGLGLHCPTYHNPADFIIEVASGEYGDLNPMLFRAVQNGLCAMAEKKSSPEKNEVPAPCPPCPPEVDPIESHTFATSTLTQFCILFKRTFLSILRDTVLTHLRFMSHVVIGVLIGLLYLHIGDDASKVFNNTGCLFFSMLFLMFAALMPTVLTFPLEMAVFMREHLNYWYSLKAYYLAKTMADVPFQVVCPVVYCSIVYWMTGQPAETSRFLLFSALATATALVAQSLGLLIGAASNSLQVATFVGPVTAIPVLLFSGFFVSFKTIPTYLQWSSYLSYVRYGFEGVILTIYGMERGDLTCLEERCPFREPQSILRALDVEDAKLYMDFLVLGIFFLALRLLAYLVLRYRVKSER

>HsABCG5 Q9H222 651 aa

MGDLSSLTPGGSMGLQVNRGSQSSLEGAPATAPEPHSLGILHASYSVSHRVRPWWDITSCRQQWTRQILKDVSLYVESGQIMCILGSSGSGKTTLLDAMSGRLGRAGTFLGEVYVNGRALRREQFQDCFSYVLQSDTLLSSLTVRETLHYTALLAIRRGNPGSFQKKVEAVMAELSLSHVADRLIGNYSLGGISTGERRRVSIAAQLLQDPKVMLFDEPTTGLDCMTANQIVVLLVELARRNRIVVLTIHQPRSELFQLFDKIAILSFGELIFCGTPAEMLDFFNDCGYPCPEHSNPFDFYMDLTSVDTQSKEREIETSKRVQMIESAYKKSAICHKTLKNIERMKHLKTLPMVPFKTKDSPGVFSKLGVLLRRVTRNLVRNKLAVITRLLQNLIMGLFLLFFVLRVRSNVLKGAIQDRVGLLYQFVGATPYTGMLNAVNLFPVLRAVSDQESQDGLYQKWQMMLAYALHVLPFSVVATMIFSSVCYWTLGLHPEVARFGYFSAALLAPHLIGEFLTLVLLGIVQNPNIVNSVVALLSIAGVLVGSGFLRNIQEMPIPFKIISYFTFQKYCSEILVVNEFYGLNFTCGSSNVSVTTNPMCAFTQGIQFIEKTCPGATSRFTMNFLILYSFIPALVILGIVVFKIRDHLISR

>HsABCG8 Q9H221 673 aa

MAGKAAEERGLPKGATPQDTSGLQDRLFSSESDNSLYFTYSGQPNTLEVRDLNYQVDLASQVPWFEQLAQFKMPWTSPSCQNSCELGIQNLSFKVRSGQMLAIIGSSGCGRASLLDVITGRGHGGKIKSGQIWINGQPSSPQLVRKCVAHVRQHNQLLPNLTVRETLAFIAQMRLPRTFSQAQRDKRVEDVIAELRLRQCADTRVGNMYVRGLSGGERRRVSIGVQLLWNPGILILDEPTSGLDSFTAHNLVKTLSRLAKGNRLVLISLHQPRSDIFRLFDLVLLMTSGTPIYLGAAQHMVQYFTAIGYPCPRYSNPADFYVDLTSIDRRSREQELATREKAQSLAALFLEKVRDLDDFLWKAETKDLDEDTCVESSVTPLDTNCLPSPTKMPGAVQQFTTLIRRQISNDFRDLPTLLIHGAEACLMSMTIGFLYFGHGSIQLSFMDTAALLFMIGALIPFNVILDVISKCYSERAMLYYELEDGLYTTGPYFFAKILGELPEHCAYIIIYGMPTYWLANLRPGLQPFLLHFLLVWLVVFCCRIMALAAAALLPTFHMASFFSNALYNSFYLAGGFMINLSSLWTVPAWISKVSFLRWCFEGLMKIQFSRRTYKMPLGNLTIAVSGDKILSVMELDSYPLYAIYLIVIGLSGGFMVLYYVSLRFIKQKPSQDW

>AtABCG1 O80946 WBC1 740 aa

MARIVAANDDDSMELNTISSIHDSTLGQLLKNVSDVRKMAIGDETPVHESLNQDYNDGYMRTVPFVLSFDNLTYNVSVRPKLDFRNLFPRRRTEDPEIAQTARPKTKTLLNNISGETRDGEIMAVLGASGSGKSTLIDALANRIAKGSLKGTVKLNGETLQSRMLKVISAYVMQDDLLFPMLTVEETLMFAAEFRLPRSLPKSKKKLRVQALIDQLGIRNAAKTIIGDEGHRGISGGERRRVSIGIDIIHDPILLFLDEPTSGLDSTSAFMVVKVLKRIAQSGSIVIMSIHQPSHRVLGLLDRLIFLSRGHTVYSGSPASLPRFFTEFGSPIPENENRTEFALDLIRELEGSAGGTRGLIEFNKKWQEMKKQSNRQPPLTPPSSPYPNLTLKEAIAASISRGKLVSGGESVAHGGATTNTTTLAVPAFANPMWIEIKTLSKRSMLNSRRQPELFGIRIASVVITGFILATVFWRLDNSPKGVQERLGFFAFAMSTMFYTCADALPVFLQERYIFMRETAYNAYRRSSYVLSHAIVSFPSLIFLSVAFAATTYWAVGLDGGLTGLLFYCLIILASFWSGSSFVTFLSGVVPSVMLGYTIVVAILAYFLLFSGFFINRNRIPDYWIWFHYMSLVKYPYEAVLQNEFSDATKCFVRGVQIFDNTPLGELPEVMKLKLLGTVSKSLGVTISSTTCLTTGSDILRQQGVVQLSKWNCLFITVAFGFFFRILFYFTLLLGSKNKRR

>AtABCG3 Q9ZUU9 WBC3 730 aa no EL3 cysteines

MEEIQSQSDLYRSSSSSASSPTSRVPSSHFFYVRKPGSLRQPISFEDSPEWEDTPDVDLRMEDEAGGGDSINDATTTPVSPSLSKMNSGSMASPPVPEGGAGTGVVRKIAGASIAWKDLTVTMKGKRKYSDKVVKSSNGYAFPGTMTVIMGPAKSGKSTLLRALAGRLPPSAKMYGEVFVNGSKSHMPYGSYGFVERETQLIGSLTVREFLYYSALLQLPGFLFQKRSVVEDAIQAMSLSDYANKLIGGHCYMKGLRSGERRRVSIARELVMRPHILFIDEPLYHLDSVSALLMMVTLKKLASMGCTLVFTIYQSSTEVFGLFDRICLLSNGNTLFFGETLACLQHFSNAGFPCPIMQSPSDHFLRAINTDFDRIIAMCKNWQDDNGDFSAVNMDTAVAIRTLEATYKSSADADSVEAMIIKLTEREGTQLKSKGKAGAATRVAVLTWRSLLVMSREWKYYWLRLILYMILTLSIGTLYSGLGHSLSSVATRVAAVFVFVSFASLLGIAGIPSLLKEIKIYRSEASNQHSGAFVFLLGQFLGSIPFLFLMSISSSLVFYFMVGLRDDFSLLMYFVLNFFMCLLVNEGLMLFIACIWRDVYWSTLTLISVHVIMMLAAGHFRIRTALPKPVWTYPFAYISFHTYSIEGLLENEYLGEVFAVGEVRSISGYQAIQGNYQISPDTNAKWRNMLVLLAMAFGYRLLVYVLLRFGLNKNVSGRLLLSHKKNNSSR

>AtABCG4 Q9SW08 WBC4 577 aa

MESYTLSTSSISYAKPLSPLLLTAEQPSFILRNITLTSHPSQILAIIGPSGAGKSTLLDILAARTSPTSGSILLNSVLINPSSYRKISSYVPQHDTFFPLLTVSETFTFSASLLLPKNLSKVSSVVASLLKELNLTHLAHTRLGQGLSGGERRRVSIGLSLLHDPEVLLLDEPTSGLDSKSAFDVVQILKSIATSRERIVILSIHQPSFKILSLIDRVLLLSKGTIVYHGRLDLLEAFLLSKGFTVPSQLNSLEYAMEILQNIRDPYENANIALPDHCPESKKQNQKQSIVRYKSSRITEISLLSSRFWKIIYRTRQLLLTNILESLVVGLVLGTIYLNIGTGKEGIRKRFGLFAFTLTFLLSSTTQTLPIFIDERPILLRETSSGLYRLSSHILANTLVFLPYLLLIAIIYSVSLYFLVGLCFSWQALAYFVLVIWIIVLMANSFVLFLSSLAPNYIAGTSSVTILLAAFFLFSGYFISKESLPKYWLFMYFFSMYKYALDALLINEYSCLHNKCLVWFEEASVNSCLVTGGDVLDKNGLHERQRWFNVYMLLGFFVLYRVLCFLVLLKRVSGSKR

>AtABCG14 Q9C6W5 WBC14 648 aa very short EL3 but 2 EL3 cysteines

MPQNCIAPRPEEDGGVMVQGLPDMSDTQSKSVLAFPTITSQPGLQMSMYPITLKFEEVVYKVKIEQTSQCMGSWKSKEKTILNGITGMVCPGEFLAMLGPSGSGKTTLLSALGGRLSKTFSGKVMYNGQPFSGCIKRRTGFVAQDDVLYPHLTVWETLFFTALLRLPSSLTRDEKAEHVDRVIAELGLNRCTNSMIGGPLFRGISGGEKKRVSIGQEMLINPSLLLLDEPTSGLDSTTAHRIVTTIKRLASGGRTVVTTIHQPSSRIYHMFDKVVLLSEGSPIYYGAASSAVEYFSSLGFSTSLTVNPADLLLDLANGIPPDTQKETSEQEQKTVKETLVSAYEKNISTKLKAELCNAESHSYEYTKAAAKNLKSEQWCTTWWYQFTVLLQRGVRERRFESFNKLRIFQVISVAFLGGLLWWHTPKSHIQDRTALLFFFSVFWGFYPLYNAVFTFPQEKRMLIKERSSGMYRLSSYFMARNVGDLPLELALPTAFVFIIYWMGGLKPDPTTFILSLLVVLYSVLVAQGLGLAFGALLMNIKQATTLASVTTLVFLIAGGYYVQQIPPFIVWLKYLSYSYYCYKLLLGIQYTDDDYYECSKGVWCRVGDFPAIKSMGLNNLWIDVFVMGVMLVGYRLMAYMALHRVKLR

>AtABCG15 Q8RWI9 WBC15 or WBC22 691 aa short EL3 and no EL3 cysteines

MELEGSSSGRRQLPSKLEMSRGAYLAWEDLTVVIPNFSDGPTRRLLQRLNGYAEPGRIMAIMGPSGSGKSTLLDSLAGRLARNVVMTGNLLLNGKKARLDYGLVAYVTQEDVLLGTLTVRETITYSAHLRLPSDMSKEEVSDIVEGTIMELGLQDCSDRVIGNWHARGVSGGERKRVSIALEILTRPQILFLDEPTSGLDSASAFFVIQALRNIARDGRTVISSVHQPSSEVFALFDDLFLLSSGESVYFGEAKSAVEFFAESGFPCPKKRNPSDHFLRCINSDFDTVTATLKGSQRIQETPATSDPLMNLATSVIKARLVENYKRSKYAKSAKSRIRELSNIEGLEMEIRKGSEATWWKQLRTLTARSFINMCRDVGYYWTRIISYIVVSISVGTIFYDVGYSYTSILARVSCGGFITGFMTFMSIGGFPSFLEEMKVFYKERLSGYYGVSVYILSNYISSFPFLVAISVITGTITYNLVKFRPGFSHYAFFCLNIFFSVSVIESLMMVVASVVPNFLMGLITGAGLIGIIMMTSGFFRLLPDLPKIFWRYPVSYISYGSWAIQGGYKNDFLGLEFEPLFPGEPKMTGEEVIEKVFGVKVTYSKWWDLAAVVAILVCYRLLFFVVLKLRERAGPALKAIQAKRTMRNLDRRPSFKRMPSLSLSLSSMSSRRHQPLRSLSSQEGLNSPIHY

>OsABCG3 XP_015627863 719 aa

MDPYRSSSSSASSPAAALAMGRRHYYLPARPARPISFEDSPDWADDDVDSIHLATASASASLPTTAYPSPSPTPSSSSAACRGGERKVAGATLVWKELSVSLTRSRSGSGSADRRVVKSSTGYALPGTLTVIMGPARSGKSTLLRAIAGRLRPAERMYGQVLLNATNTRLPYGSYGFVDRHDVLIDSLTVREMLYYSAHLQLPGLFSSKTSIVEDAIAAMSLADYADNLIGGHCFINSLPAGERRRLSIARELVMRPHVLFIDEPLYHLDSVSALLLMVTLKKLASTGCTVIFTMYQSSTEVFGLFDRICLLSNGNTLFFGETLSCLQHFSNAGFPCPIMQSPSDHFLRAINTDFDRIIAMCKNLQDDQGDFSSVSMDTAVAIRTLEATYKSSADSVAVESLVAKLMEKEGPHLKSKGRASNTTRIGVLTWRSLVIMSRNRKYFWSRFALYMLLALSVGTIFNNAGHSLSSVMVRVSAIFVYVSFVILLSVSGVPAHIDEIKIYSHEEANQHSSTMVFLLGHFLSSIPFLFLVTISSSLVFYFLIGLRNEFNLFMYFVVTMFMCLLANEALMMIVAYIWLDTYKCTLTLICLYVIMMLVGGYFRIRGGLPCTVWKYPLSYVSFHVYAVEGLLENEYVGTSFAVGAIRTIPGVQAVGGSYDISSSANAKWVNLLVLFVMAVGYRVVLYVLLRLNVRKHMRLLGSWCCWSWTPQSDYYSSN

>OsABCG4 XP_015641867 624 aa

MSTTTTTTVTEPDNAEASPSPSPSPSTPPKKVIMYELAARNIYYAKPAAAAVATTTVASLARLLRPCGAAQPPSPEYILRDVSLTARPGEILAVVGPSGAGKSTLLDILAARTAPTHGRLLLNAAPLRPSSFRRLSAHVPQMDVALPLLTVAETFAFAASLLYPAAAEASAAVAALLADLRLGHAAHTRVSATRLSGGERRRVSIGLALLRDPGVLLLDEPTSGLDSSSAHVVVGCLRAVAAARGTTVVLSIHQPSSRLLSAVDSLLLLSRGAVVHHGSVDSLDAALLSHGLAVPAQLNPLEFALEVLDQMPHPSASSPEPKTTEELAAVTSSKSSSSSTSPCSRIHEVVVLYKRAWKVVYRSKQLLLTNFLESVVVGTLLGSIYINAGDGEGGAHKRLGLFAFTLTFLLTSTTETLPTFVSERPIVLAETASGLYRLSSHAAAATLVFLPYLLAVALLYSACVYFLVGLCASAAAFAAFVMVVWAVVLTANSFVLFVSSFAPDYIAGMSLVSVSLAGFFLFSGYFLSRGSMPPYWVFMHYVSPYKYALDALLANEYTCAATRCFGVAGPAAGDCSETGADVLAEKGLTAKERWTGVQVLFGFFLLYRVLYWVVLSRRAARAKR

>OsABCG5 XP_015628264 787 aa

MSRFVDKLPLFDRRPSPMEEAEGLPRSGYLGQLHHHQYYQPHSNMLPLEQSPPTSTKHTSVTLAQLLKRVNDARSGSSTPISSPRYTIELGGSKPESVSSESDDHHSDDGGSEGQPRALVLKFTDLTYSVKQRRKGSCLPFRRAAADEPELPAMRTLLDGISGEARDGEIMAVLGASGSGKSTLIDALANRIAKESLHGSVTINGESIDSNLLKVISAYVRQEDLLYPMLTVEETLMFAAEFRLPRSLPTREKKKRVKELIDQLGLKRAANTIIGDEGHRGVSGGERRRVSIGVDIIHNPIMLFLDEPTSGLDSTSAFMVVTVLKAIAQSGSVVVMSIHQPSYRILGLLDRLLFLSRGKTVYYGPPSELPPFFLDFGKPIPDNENPTEFALDLIKEMETETEGTKRLAEHNAAWQLKHHGEGRGYGGKPGMSLKEAISASISRGKLVSGATDGTVSVAASDHSAPPPSSSSVSKFVNPFWIEMGVLTRRAFINTKRTPEVFIIRLAAVLVTGFILATIFWRLDESPKGVQERLGFFAIAMSTMYYTCSDALPVFLSERYIFLRETAYNAYRRSSYVLSHTIVGFPSLVVLSFAFALTTFFSVGLAGGVNGFFYFVAIVLASFWAGSGFATFLSGVVTHVMLGFPVVLSTLAYFLLFSGFFINRDRIPRYWLWFHYISLVKYPYEAVMQNEFGDPTRCFVRGVQMFDNTPLAALPAAVKVRVLQSMSASLGVNIGTGTCITTGPDFLKQQAITDFGKWECLWITVAWGFLFRILFYISLLLGSRNKRR

>OsABCG12 XP_015637534 700 aa

MEYGGGREGGGGMNGNGTAYAGALSPAARYAESGGASLTWENLTAVLPGGGGRATKKLVQGLYGYAVPGRVVAIMGPSGSGKSTLLDALSGRLARNVLLTGKVLLNGKKRRLDYGVLAYVTQENVLLGTLTVRETVTYSALLRLPSTMSKAEVRRVVDDTLDEMGLRECADRNIGNWHLRGISGGEKKRLSIALEILTRPRLLFLDEPTSGLDSASAFSVIETLRQLAVDGGRTIVSSVHQPSSEVFALFDDLCLLSSGECVYFGDAKLAPQFFAETGFPCPSRRNPSDHFLRCVNADFDDVAAAMKGSMKLRAEADFDPLLKYSTSEIRERLVDKYRISEYAMMVRNTIHEISKIEGVIEEVVMGSQASWCKQLTTLTRRSFTNMSRDFGYYWLRIVIYVLMAVCLGTIYYDVGTSYAAIQARASCGGFVSGFMTFMSIGGFPSFIEEMKMFTLERQNGHYGVAAYIISNFLSSMPFLLAVSWASASITYWMVKFRPGFSYFAFFALNLYGGVSVIESLMMIISALVPNFLMGLILGAGVIGIMMLTSGFFRLLPELPKIFWRYPVSYIVYGSWGLKGAYKNDLIGLEFEPMMPGQPKLTGEYIITKMMGLSLNHSKWLDLSMIFVLLFAYRLIFFLVLKAKEAAAPYIRVAYTRFTIKRLERRASFRKTLAMSSMSKRHNQPPHPMAVQEGLNSPMPY

>OsABCG14 XP_015650927 757 aa

MPPQELHGDDHGRHHHHQTPAGSGGGGAGDGHHRDFSSPPSTTSSSSSSSSSSSTNSPTATAASSSSTNTSGAAIVHPTTSSHPSVASGHHSAANSYPLVLKFEEVVYKVKIGKPAAGWCARMSSAIGGGGEGRRKKGAAAVAKEKTIISGMSGVVRPGEMLAMLGPSGSGKTTLLTALGGRHGGGGGGGRGMLSGKITYNGQPFSGAVKRRTGFVTQHDVLYPHLTVAETLWYTALLRLPRALGAGEKRAQAEEVMLELGLGKVAHSMIGGVRGVRGLSGGERKRVSIGLEMLVDPSLLLLDEPTSGLDSTTAARIVGTLRRMAAGGGRTVVVTIHQPSSRLYHMFDKVLLLSSDGCPIYYGLAADALSYFASVGFASPLSLNPADLMLDLANGIAPQISGGGGDGDVGGGAAAAAANGGGSEAELKEVRGKLAAAYERHIAPAVKLDICAREGGGGGGQGAAAAAAVAGRRRRGGGKAAAEQWTNGWWAQFTVLLRRGVKERRYESFNKLRIFQVLSVASLAGLLWWRTPAAHLQDRTALIFFFSVFWGFFPLYNAVFTFPLERPMLVKERSSGMYRLSSYFAARTAADLPMELALPTAFVVILYWMGGLDPRPGPFLLSLLVVLYSVLVAQSLGLAIGAVLMDVKQGTTLASVITMVFLIAGGYYVQHIPPFVGWLRWLNYSFYCYRLLIGIQFGDGAAHYDCGGGGARCLVADFPAIKAVGLNNHWVDVCVMALLLVGYRVIAYLALDRLKPR

>Cne19.3120 626 aa very short EL3 but 2 EL3 cysteines

MSAVAIDPSYVEAGKCLDITGLYNDSVSSFHWRGITATMPASGSKAEKTLLAAVSGEAHAGERIPDDTMLTAGELVAIMGPSGSGKTTLLNRLAHRAMPPKAKLAGDIFINDVHATISDIRRTSCYVEQQDHHIGSITTAETLAFAAKFGLDEPIGKAELRQRVDMLLSSFGLKDQKNMIIGTPIQKGLSGGQKRRVSVASQLITSPKILFLDEPTSGLDSVASFEIVSYLKTVARKYKLLVIASIHQPSTKTFNVFDQIFLLAKGRLCYGGARSELSTYFASIGLEMPAQTNPAEWILEIVDTDFAKDQVEGLQRLERITNAWASDQKLSDVIPAKGLAHSTRSRRTSFMLPFHLFHRNFIKSYRDLIAYWIRVGMYTCLAILMGTSWLRLGYSQDDINARITAIFFSGAFLSFMAVAYIPAYIEDQETFFKERANGLYGPLSFLVANFLIGIPYLFIIVISFSVISYWMVGLWPTATGFWTFVGFLFLDLLAAESLVVFVASLVPNFIVALALVAFANGLWMVTNGFLIPETILNVFWRSWVTKIDYQNWAFRAMMWNEFHQQTFNCGRLSCSFPSLDGRHISGTLVLEYYGYTGGQLGAYAGYMIAIVMGYRLLAWLTLTLRK

>Afu19.3120 XP_755735 628 aa very short EL3 but 4 EL3 cysteines

MGTDIMAPDLERNDRDQFLVNHTVHNFSWNDLTVTVKDRRTKKPLNLIEGISGSIQQGELVALMGPSGCGKTTLLNVLARRAATSGAKTTGECYVNGGALDNATFGRITSYVEQEDALIGSLTVQETLKFAADLSLPSSVSKAQRRDRIQTLLESFGILNQAATLVGTPIRKGISGGQKRRVSVASQLITCPKICFLDEPTSGLDSTASYEVISYVKELAVANNLIVIASIHQPSTTTFQLFDKLLLLSKGKSCYFGPVPQISTYFGSIGHPIPLNTNPAEFILDIVSSDFSDAKEGNAAERVRHIQESWLQSAERRAVDNQISQLIEHPEQDRKKITMGELSRPNTASITWSLLHRSFIKSYRDVVAYGIRIVMYLGLAIMMGTVWLRLHESQEYIQPFINAIFFGSAFMSFMAVAYVPAFLEDRATFIKERANGLYGALPFIISNFIIGLPFLFLISLLFSLVAYWLSNFCSDAVAFFTWVMWLFLDLLAAESLVVFVTSIFPNFVIALALVAFANGLWMSVGGFLVSPTILNPFWKYVFHYIDYQAYVFQGMMVNEFSRRTYSCGNGCHCMYQTDLASQCRIRGTGVLESYGYATGRTGKWVGILIGIIAVYRLFGWIALVLRRT

>Fg19.3120 XP_011324145 625 aa very short EL3 but 4 EL3 cysteines

MALKGSDHSMDSEQRPVAEKHLVNTTIKNFTWSNVTVTVKDRETKQPKAIVDDVQGIVEAGEICALMGPSGCGKTTLLNVLARRPTNASNAEAQVLANGKHLSLAEFREVSCFVEQEDALIGSLTVRETLEFSSRLASSSSLSKKERNVRIDNLLESFGLVEQANTLIGTPIRKGISGGQKRRVGVASQLITSPKLLFLDEPTSGLDSAASLEVVKYLRAVAKRNNLIVICSIHQPSTSTFNLFDKLLLLSGGKTHYFGPVSSVTSYYAQVGAPLPQYVNPAEHLLELVNIDFAQNRGEASRNLGSLQDSWATSQQASQVSDAIKTAESSGGDWSVETIEKRPSMPSLTLTLLHRSFIKSYRDVVAYGIRLAMYLGLAIMMGTVWLRLKPEQESIQPFINAIFFGSAFMSFMAVAYVPAFIEDRLQYVKEHHNGLYGATELILSNFLIGIPYLFIISALFSVISYWLSNFQPTATAFFTWVMWLFLDLLAAESLVVFMTSLFPSFVISLALVAFANGLWMSVGGFMVPPTILNVFYKYVFHYWDYQKYVFEGMMVNEFSKRVYSCGNGCRCMYQSALADQCKIDGQAVLDQYGYSSGHLGRNVGIMISIIAGYRIAAWLVLVLRR

>CaORF19.3120 579 aa very short EL3 but 4 EL3 cysteines

MSVETYSWSNISLTLQNGKTILDDIYGSVSAGEMLAIMGPSGCGKSTLLNVLAYRTSPRSSTLEGGIFINNERATLNKIKQLSSYVEQEDSLIGSLTVSETVDYSAQFAGIDKAHKKELVSKTIKSLGLEGQAMSKIGTPIQKGISGGQKRRVSIASQIITSPSILFLDEPTSGLDSVASREVISTIKKIAKRENMVIICSIHQPSTYTFELFDKVMFLSKGRTVYNGAVSNVVKYFNSIGHTMPPYINPAEYVLDLINTDFQGDSSVLDDLVSKWNSGDVHKVGTESVQLTEATTINEMQNILILIARSLTKARRDILTYYVRLVMYLGLAILMGTVWLRLQNGQKNIQPFINAIFFSGAFMSFMSVAYIPSYLEDIQSYKKERMNGLYGPLAFSLANFLVGLPFLFLIAAVFSVITFFMVNFHQTASGFWYYLMWLFLDLVAAESMTTFIASVFPNFVVSLAITAFANGLWMAVGGFLVPSNILNVFWYYTFYWIDYQRYVFQGMIFNEFTNREFRCGDGCHCMYDSPLASQCKISGKAVLESLGYGNYDKGLWIGVLIALVFVYRFATYIVLKLRK

>CtORF19.3120 584 aa

MTVQTFSWSNLTLTLDNGKVLLDDVSGSISESGLYALMGPSGLGKTTLLNSLAFRSKPGSSHLSGDIYINDEEATLNKIKQLSSYVEQEDSLIGSLRVLETVDYSAKFAGINSAHRKDLVEKTIKSLGLSNQLNVKIGTPILKGISGGQKRRVSIASQMITSPSILFLDEPTSGLDSVASREVISTIKKVAKEENMIVICSIHQPSTYTFELFDKVMFLSRGKTVYNGKVEDVVKYFDSVGYPMPPYMNPAEYVLDLINTDFDSNGEILDDLVSKWKENEHDHSEILKLESDPIELTTFSTTSEIRNIGTLISRSLVKASRDILTYYVRLVMYLGLAILMGTVWLRLGKDQKNIQPFINAIFFSGAFMSFMSVAYIPSYLEDYSSYKKERLNGLYGPFAFCLSNFLVGLPFLFLISAVFSIVTFFMVNFHQTATGFWYYLFWLFLDLVAAESMTTLIASVFPNFVISLALTAFANGLWMSVGGFLVSTNILNVFWYYTFYWVNYQRYVFQGMMFNEFIPRVFNCGEGCHCMYDSPLANQCQITGRAVLQNLGYGHEDRGLWIGVMIAIIAFYRIATYIVLKFRK

>AtABCG31 Q7PC88 PDR3 1426 aa

MAAASNGSEYFEFDVETGRESFARPSNAETVEQDEEDLRWAAIGRLPSQRQGTHNAILRRSQTQTQTSGYADGNVVQTIDVKKLDRADREMLVRQALATSDQDNFKLLSAIKERLDRVGMEVPKIEVRFENLNIEADVQAGTRALPTLVNVSRDFFERCLSSLRIIKPRKHKLNILKDISGIIKPGRMTLLLGPPGSGKSTLLLALAGKLDKSLKKTGNITYNGENLNKFHVKRTSAYISQTDNHIAELTVRETLDFAARCQGASEGFAGYMKDLTRLEKERGIRPSSEIDAFMKAASVKGEKHSVSTDYVLKVLGLDVCSDTMVGNDMMRGVSGGQRKRVTTGEMTVGPRKTLFMDEISTGLDSSTTFQIVKCIRNFVHLMDATVLMALLQPAPETFDLFDDLILLSEGYMVYQGPREDVIAFFESLGFRLPPRKGVADFLQEVTSKKDQAQYWADPSKPYQFIPVSDIAAAFRNSKYGHAADSKLAAPFDKKSADPSALCRTKFAISGWENLKVCFVRELLLIKRHKFLYTFRTCQVGFVGLVTATVFLKTRLHPTSEQFGNEYLSCLFFGLVHMMFNGFSELPLMISRLPVFYKQRDNSFHPAWSWSIASWLLRVPYSVLEAVVWSGVVYFTVGLAPSAGRFFRYMLLLFSVHQMALGLFRMMASLARDMVIANTFGSAAILIVFLLGGFVIPKADIKPWWVWGFWVSPLSYGQRAIAVNEFTATRWMTPSAISDTTIGLNLLKLRSFPTNDYWYWIGIAVLIGYAILFNNVVTLALAYLNPLRKARAVVLDDPNEETALVADANQVISEKKGMILPFKPLTMTFHNVNYYVDMPKEMRSQGVPETRLQLLSNVSGVFSPGVLTALVGSSGAGKTTLMDVLAGRKTGGYTEGDIRISGHPKEQQTFARISGYVEQNDIHSPQVTVEESLWFSASLRLPKEITKEQKKEFVEQVMRLVELDTLRYALVGLPGTTGLSTEQRKRLTIAVELVANPSIIFMDEPTSGLDARAAAIVMRTVRNTVDTGRTVVCTIHQPSIDIFEAFDELLLMKRGGQVIYGGKLGTHSQVLVDYFQGINGVPPISSGYNPATWMLEVTTPALEEKYNMEFADLYKKSDQFREVEANIKQLSVPPEGSEPISFTSRYSQNQLSQFLLCLWKQNLVYWRSPEYNLVRLVFTTIAAFILGTVFWDIGSKRTSSQDLITVMGALYSACLFLGVSNASSVQPIVSIERTVFYREKAAGMYAPIPYAAAQGLVEIPYILTQTILYGVITYFTIGFERTFSKFVLYLVFMFLTFTYFTFYGMMAVGLTPNQHLAAVISSAFYSLWNLLSGFLVQKPLIPVWWIWFYYICPVAWTLQGVILSQLGDVESMINEPLFHGTVKEFIEYYFGYKPNMIGVSAAVLVGFCALFFSAFALSVKYLNFQRR

>AtABCG36 Q9XIE2 PDR8 Cd resistance 1469 aa

MDYNPNLPPLGGGGVSMRRSISRSVSRASRNIEDIFSSGSRRTQSVNDDEEALKWAAIEKLPTYSRLRTTLMNAVVEDDVYGNQLMSKEVDVTKLDGEDRQKFIDMVFKVAEQDNERILTKLRNRIDRVGIKLPTVEVRYEHLTIKADCYTGNRSLPTLLNVVRNMGESALGMIGIQFAKKAQLTILKDISGVIKPGRMTLLLGPPSSGKTTLLLALAGKLDKSLQVSGDITYNGYQLDEFVPRKTSAYISQNDLHVGIMTVKETLDFSARCQGVGTRYDLLNELARREKDAGIFPEADVDLFMKASAAQGVKNSLVTDYTLKILGLDICKDTIVGDDMMRGISGGQKKRVTTGEMIVGPTKTLFMDEISTGLDSSTTFQIVKCLQQIVHLNEATVLMSLLQPAPETFDLFDDIILVSEGQIVYQGPRDNILEFFESFGFKCPERKGTADFLQEVTSKKDQEQYWVNPNRPYHYIPVSEFASRYKSFHVGTKMSNELAVPFDKSRGHKAALVFDKYSVSKRELLKSCWDKEWLLMQRNAFFYVFKTVQIVIIAAITSTLFLRTEMNTRNEGDANLYIGALLFGMIINMFNGFAEMAMMVSRLPVFYKQRDLLFYPSWTFSLPTFLLGIPSSILESTAWMVVTYYSIGFAPDASRFFKQFLLVFLIQQMAASLFRLIASVCRTMMIANTGGALTLLLVFLLGGFLLPKGKIPDWWGWAYWVSPLTYAFNGLVVNEMFAPRWMNKMASSNSTIKLGTMVLNTWDVYHQKNWYWISVGALLCFTALFNILFTLALTYLNPLGKKAGLLPEEENEDADQGKDPMRRSLSTADGNRRGEVAMGRMSRDSAAEASGGAGNKKGMVLPFTPLAMSFDDVKYFVDMPGEMRDQGVTETRLQLLKGVTGAFRPGVLTALMGVSGAGKTTLMDVLAGRKTGGYIEGDVRISGFPKVQETFARISGYCEQTDIHSPQVTVRESLIFSAFLRLPKEVGKDEKMMFVDQVMELVELDSLRDSIVGLPGVTGLSTEQRKRLTIAVELVANPSIIFMDEPTSGLDARAAAIVMRAVRNTVDTGRTVVCTIHQPSIDIFEAFDELMLMKRGGQVIYAGPLGQNSHKVVEYFESFPGVSKIPEKYNPATWMLEASSLAAELKLSVDFAELYNQSALHQRNKALVKELSVPPAGASDLYFATQFSQNTWGQFKSCLWKQWWTYWRSPDYNLVRFIFTLATSLLIGTVFWQIGGNRSNAGDLTMVIGALYAAIIFVGINNCSTVQPMVAVERTVFYRERAAGMYSAMPYAISQVTCELPYVLIQTVYYSLIVYAMVGFEWKAEKFFWFVFVSYFSFLYWTYYGMMTVSLTPNQQVASIFASAFYGIFNLFSGFFIPRPKIPKWWIWYYWICPVAWTVYGLIVSQYGDVETRIQVLGGAPDLTVKQYIEDHYGFQSDFMGPVAAVLIAFTVFFAFIFAFCIRTLNFQTR

>AtABCG42 Q7PC82 PDR14 general defense protein 1392 aa

MTMSQTDGVEFASRNTNENGHDDDDQLRSQWVAIERSPTFERITTALFCKRDEKGKKSQRRVMDVSKLDDLDRRLFIDDLIRHVENDNHVLLQKIRKRIDEVGIDLPKIEARFSDLFVEAECEVVYGKPIPTLWNAISSKLSRFMCSNQAKKISILKGVSGIIRPKRMTLLLGPPSCGKTTLLLALSGRLDPSLKTRGDISYNGHLFSEFVPEKTSSYVSQNDLHIPELSVRETLDFSGCFQGTGSRLEMTKEISRREKLKGIVPDPDIDAYMKAASIEGSKTNLQTDYILKILGLTICADTRVGDASRPGISGGQKRRLTTGEMIVGPIKTLFMDEISNGLDSSTTFQILSCLQQFARLSEGTILVSLLQPAPETFELFDDLILMGEGKIIYHGPRDFVCSFFEDCGFKCPNRKSVAEFLQEVISRKDQEQYWCHIEKTYCYVSIESFIEKFKKSDLGLELQDRLSKTYDKSQTQKDGLCFRKYSLSNWDMLKACSRREFLLMKRNSFVYVFKSGLLIFIGFIAMTVYLRTGSTRDSLHANYLMGSLFFSLFKLLADGLPELTLTISRIAVFCKQKELYFYPAWAYAIPSAILKIPISFLESFLWTMLTYYVIGYSPEMGRFIRQFLILFALHLSCISMFRAIAAVFRDFVVATTVGSISIVLLSVFGGFIVRKPSMPSWLEWGFWLSPLSYAEIGLTANEFFAPRWGKITSENRTLGEQVLDARGLNFGNQSYWNAFGALIGFTLFFNTVFALALTFLKTSQRSRVIVSHEKNTQSSENDSKIASRFKNALPFEPLTFTFQDVQYIIETPQGKKLQLLSGVTGAFKPGVLTALMGVSGAGKTTLLDVLSGRKTFGDIKGQIEVGGYVKVQDTFSRVSGYCEQFDIHSPNLTVQESLKYSAWLRLTSNISSETKCAIVNEVLETIELEEIKDSIVGIPGISGLTTEQRKRLTIAVELVSNPSIIFMDEPTTGLDARAAAIVMRAVKNIAETGRTVVCTIHQPSIDIFEAFDELILMKNGGKIIYYGPLGQHSSKVIEYFMRIHGVPKLKENSNPATWILDITSKSSEDKLGVDLAQMYEESTLFKENKMVIEQTRCTSLGSERLILSSRYAQTSWEQFKACLWKQHLSYWRNPSYNLTRIIFMSFTCMLCGILFWQKAKEINNQQDLFNVFGSMFTVVLFSGINNCSTVLFSVATERNVFYRERFSRMYNSWAYSLAQVLVEIPYSLFQSIVYVIIVYPMVGYHWSVFKVFWSFYSIFCTLLIFNYFGMLLVVVTPNVHIAFTLRSSFYAIVNLFAGYVMPKPNIPRWWIWMYYLSPTSWVLNGLLTSQYGDMEKEILAFGEKKKVSDFLEDYFGYRYDSLALVAVVLIAFPILLASLFAFFIGKLNFQKK

>OsABCG31 PDR6 Q8GU87 1426 aa

MWAAEAAFARSGSWREEEDEQEALRWAALQRLPTVARARRGLLRSPAPGEDRVQGDDALCEVDVAGLSPGDRTALVDRLLADSGDVEDFFRRIRSRFDAVQIEFPKIEVRYEDLTVDAYVHVGSRALPTIPNFICNMTEAFLRHLRIYRGGRVKLPILDNVSGIIRPSRMTLLLGPPSSGKTTLLLALAGRLGPGLKVSGNITYNGHHLNEFVPQRTSAYVSQQDWHASEMTVRETLEFAGRCQGVGIKYDMLVELLRREKNEGIKPDEDLDVFMKALALEGKQTSLVAEYIMKVYGLDICADTIVGDEMIKGISGGQKKRLTTGELLVGSARVLFMDEISTGLDSATTYQIIKYLRHSTHALDGTTIISLLQPAPETYELFDDVILISEGQIVYQGPREYAVDFFAGMGFRCPERKNVADFLQEVLSKKDQQQYWCHYDYPYQYVSVSKFAEAFKTFVIGKRLHDELAVPYNRHRNHPAALSTSNYGVRRLELLKSNFQWQHLLMKRNSFIYVFKFIQLLLVALITMTVFFRSTMHRDSVDDGIIYLGALYFAIVMILFNGFTEVSLLVTKLPILYKHRDLHFYPPWAYTLPSWLLSIPTSLIESGMWVLVTYYVVGYDPQFTRCLGQFLLLFFLHQTSLALFRVMASLGRNMIVANTFGSFALLVVMILGGFIITKESIPAWWIWGYWISPMMYAQNAISVNEFLGHSWSQQFANQNITLGEAILTGYGLFKEKYWFWIGVGALFGYAIVLNFLFTLFLTLLNPIGNIQAVVSKDDIQHRAPRRKNGKLALELRSYLHSASLNGHNLKDQKGMVLPFQPLSMCFKNINYYVDVPAELKSQGIVEDRLQLLIDVTGAFRPGILTALVGVSGAGKTTLMDVLAGRKTGGLIEGSITISGYPKNQETFTRISGYCEQNDVHSPCLTVIESLLYSACLRLPSHVDVNTRRVFVEEVMELVELNALSGALVGLPGVNGLSTEQRKRLTIAVELVANPSIVFMDEPTSGLDARSAAIVMRTVRNIVNTGRTIVCTIHQPSIDIFESFDELLFMKRGGQLIYAGPLGSKSRNLVEFFEAIPGVPKIRDGYNPAAWMLEVTSTQMEQILGVDFAEYYRQSKLFQQTQEMVDILSRPRRESKELTFATKYSQPFFAQYAACLWKQNLSYWRNPQYTAVRFFYTVIISLMFGTICWKFGSRRETQHDIFNAMGAMYAAVLFIGITNATSVQPVISIERFVSYRERAAGMYSALPFAFSLVTVEFPYILVQSLIYGTIFYSLGSFEWTAVKFLWYLFFMYFTLLYFTFYGMMTTAITPNHTVAPIIAAPFYTLWNLFCGFMIPRKRIPAWWRWYYWANPVSWTLYGLLTSQFGDLDQPLLLADGITTTTAVDFLRDHFGFRHDFLGVVAGMVAGFCVLFAVVFALAIKYLNFQRR

>OsABCG36 PDR9 Q0JLC5 1457 aa

MDAAGEIQKVASMRLGGSMRGDSGSMWRRGDDVFSRSSREEDDEEALRWAALEKLPTYDRVRRAILPLGGDDGAGDGGGKGVVDVHGLGPRERRALLERLVRVADEDNEKFLLKLKDRVDRVGIDMPTIEVRFEHLEAEAEVRVGNSGLPTVLNSITNTLEEAGNALGILPNRKQTMPVLHDVSGIIKPRRMTLLLGPPGSGKTTLLLALAGRLGKDLKASGKVTYNGHGMEEFVPERTAAYISQHDLHIGEMTVRETLAFSARCQGVGSRFDMLTELSRREKAANIKPDADIDAFMKAAAMGGQEANVNTDYILKILGLEICADTMVGDEMLRGISGGQRKRVTTGEMLVGPARALFMDEISTGLDSSTTFQIVNSLRQTVHILGGTAVISLLQPAPETYNLFDDIILLSDGQIVYQGPREDVLEFFESMGFKCPDRKGVADFLQEVTSKKDQRQYWARHDKPYRFVTVKEFVSAFQSFHTGRAIANELAVPFDKSKSHPAALATTRYGAPGKELLKANIDREILLMKRNSFVYMFRTFQLMVVSLIAMTLFFRTKMKRDSVTSGGIYMGALFFGVLMIMFNGFSELALTVFKLPVFFKQRDLLFYPAWSYTIPSWILKIPITFIEVGGYVFLTYYVIGFDSNVGSFFKQYLLMLAINQMAGSLFRFIGGAARNMIVANVFASFMLLIFMVLGGFILAREQVKKWWIWGYWISPMMYAQNAISVNELMGHSWNKIVNSSASNETLGVQVLKSRGVFPEARWYWIGFGAMIGFTILFNALFTLALTYLRPYGNSRQSVSEEELKEKRANLNGEIVGDVHLSSGSTRRPMGNGTENDSTIVDDDTEVTQRGMVLPFTPLSLSFDNVRYSVDMPQEMKAQGVADDRLELLKGVSGSFRPGVLTALMGVSGAGKTTLMDVLAGRKTGGYIEGSINISGYPKKQETFARVSGYCEQNDIHSPQVTVYESLLFSAWLRLPEDVDSNTRKMFIEEVMELVELKSLRDALVGLPGVNGLSTEQRKRLTIAVELVANPSIIFMDEPTSGLDARAAAIVMRTVRNTVNTGRTVVCTIHQPSIDIFEAFDELFLMKRGGEEIYAGPLGHHSSELIKYFESIPGVSKIKDGYNPATWMLEVTTIGQEQALGVDFSDIYKKSELYQRNKALIKDLSQPAPDSSDLYFPTQYSQSSLTQCMACLWKQNLSYWRNPPYNAVRFFFTTVIALLFGTIFWDLGGKVTKSQDLFNAMGSMYAAVLFIGVMNCTSVQPVVAVERTVFYRERAAGMYSAFPYAFGQVVIEIPYTLVQATVYGIIVYAMIGFEWTAAKFFWYLFFMVFTLLYFTFYGMMAVGLTPNYHIASIVSSAFYAIWNLFSGFVIPRPRVPIWWRWYCWACPVAWTLYGLVVSQFGDIETPMEDGTPVKVFVENYFGFKHSWLGWVATVVAAFAFLFASLFGFAIMKFNFQKR

>OsABCG51 PDR13 Q8S628 1441 aa

MAFAAGGIDHHVAVDVEGEEESRRRAVAEEADLLWAAFERLPSAKRRSHAVVLPDPDGLGGGDGGGRGEGQLVDVRKLDRPGLQRVLRHALATSELDNANLLHGIKARFDAVGLEVPRVEVRFQNLTVSTDVHVGRRALPTLVNYVHDIAERILISSHLLRPDKHKLVILDDVSGVIKPGRMTLLLGPPASGKSTLLLALADKLDSQLKKSGEVAYNGMALDQFCVQRTSAYISQTDNHIGELTVRETLDFAAKCQGASENWQECLKELVNLEKERGIRPSPEIDAFMKTASFRREKHNLVSDYVLRVLGLDICADTPVGSDMERGVSGGQKKRVTTGEMIIGPRKTLLMDEISTGLDSSTTFQIVNCMRNFVHEMEATVLMSLLQPAPETFELFDDLILLSEGKIIYQGPIKHVVDYFKSLGFSLPPRKGIADFLQEVTSKKDQAQYWSDQSKQHIFVSASEMAAVFKESQYGTYLEANLSSSCGNKDSALVLPRSKFAVPKFSLVRACFARELILISRNRFLYTFRTCQVAFVGIITSTLFLRTRLHPVDEQNGNLYLACLFFGLVHMMFNGFTEMTMTISRLPVFYKQRDNFFHPAWAFSLPNWILRIPYSFIEAVVWSCVVYYTVGFAPTVDRFFRFMLLLFSIHQMALGLFRMMGAIARDMTIASTFGSAVLLAIFLLGGFVVPKGFIKPWWDWAYWISPLMYAQRAVSVNEFSASRWSKVSVSGNMTVGTNILISHSLPTDDHWFWIGVGVLLAYSIFFNIMFTLALAFLNPLRKPQSMVPSDAGDGRDVHINTDSNKNTIGEIFENNDGFEGQTECKSKKGMILPFQPLTMTFHNVNYYVNMPKEMQAKGVPEKRLQLLSEVSGIFRPRVLTALVGASGSGKTTLMDVLAGRKTGGYIEGDIRISGHKKEQRTFARIAGYVEQNDIHSPQVTVEESLWFSSTLRLPNDISRETRHAFVEEVMALVELDQIRYALVGKQGLTGLSTEQRKRLTIAVELVANPSIIFMDEPTSGLDARAAAIVMRTVRNTVDTGRTVVCTIHQPSIDIFEAFDELLLMKRGGRVIYGGSLGVNSVDMINYFQGIPRVVPITEGYNPATWMLEVTTQASEERLGIDFATVYKNSYQFRNVENLIVELSIPASGTEPLKFSSEFSQNRLTQFMVCLRKQSLVYWRSPEYNVVRLFFTSVAAIIFGSIFWNVGMKRESTEDILLLMGALYAACLFLGVNNASSVQPVVSVERTVYYRERAANMYSSFPYAAAQVYHGLVEIPYIAVQTLIFGLITYFMVNYERNIRKLVLYLIYMFLTFTYFTFYGMVAVGLTPTQHMASVVSSAFYSLWNLLSGFLIPQSRIPGWWIWFYYICPVAWTLRGVITSQLGDVDTRIVGPGFDGTVHEFLQQNLGFEQGMTGATVAVLVAFSVFFFSIYAISIKMINFQRR

>ScPDR5 [NP_014796.3](https://www.ncbi.nlm.nih.gov/protein/NP_014796.3?report=genbank&log$=protalign&blast_rank=1&RID=DDS6M3R4016) 1511 aa

MPEAKLNNNVNDVTSYSSASSSTENAADLHNYNGFDEHTEARIQKLARTLTAQSMQNSTQSAPNKSDAQSIFSSGVEGVNPIFSDPEAPGYDPKLDPNSENFSSAAWVKNMAHLSAADPDFYKPYSLGCAWKNLSASGASADVAYQSTVVNIPYKILKSGLRKFQRSKETNTFQILKPMDGCLNPGELLVVLGRPGSGCTTLLKSISSNTHGFDLGADTKISYSGYSGDDIKKHFRGEVVYNAEADVHLPHLTVFETLVTVARLKTPQNRIKGVDRESYANHLAEVAMATYGLSHTRNTKVGNDIVRGVSGGERKRVSIAEVSICGSKFQCWDNATRGLDSATALEFIRALKTQADISNTSATVAIYQCSQDAYDLFNKVCVLDDGYQIYYGPADKAKKYFEDMGYVCPSRQTTADFLTSVTSPSERTLNKDMLKKGIHIPQTPKEMNDYWVKSPNYKELMKEVDQRLLNDDEASREAIKEAHIAKQSKRARPSSPYTVSYMMQVKYLLIRNMWRLRNNIGFTLFMILGNCSMALILGSMFFKIMKKGDTSTFYFRGSAMFFAILFNAFSSLLEIFSLYEARPITEKHRTYSLYHPSADAFASVLSEIPSKLIIAVCFNIIFYFLVDFRRNGGVFFFYLLINIVAVFSMSHLFRCVGSLTKTLSEAMVPASMLLLALSMYTGFAIPKKKILRWSKWIWYINPLAYLFESLLINEFHGIKFPCAEYVPRGPAYANISSTESVCTVVGAVPGQDYVLGDDFIRGTYQYYHKDKWRGFGIGMAYVVFFFFVYLFLCEYNEGAKQKGEILVFPRSIVKRMKKRGVLTEKNANDPENVGERSDLSSDRKMLQESSEEESDTYGEIGLSKSEAIFHWRNLCYEVQIKAETRRILNNVDGWVKPGTLTALMGASGAGKTTLLDCLAERVTMGVITGDILVNGIPRDKSFPRSIGYCQQQDLHLKTATVRESLRFSAYLRQPAEVSIEEKNRYVEEVIKILEMEKYADAVVGVAGEGLNVEQRKRLTIGVELTAKPKLLVFLDEPTSGLDSQTAWSICQLMKKLANHGQAILCTIHQPSAILMQEFDRLLFMQRGGKTVYFGDLGEGCKTMIDYFESHGAHKCPADANPAEWMLEVVGAAPGSHANQDYYEVWRNSEEYRAVQSELDWMERELPKKGSITAAEDKHEFSQSIIYQTKLVSIRLFQQYWRSPDYLWSKFILTIFNQLFIGFTFFKAGTSLQGLQNQMLAVFMFTVIFNPILQQYLPSFVQQRDLYEARERPSRTFSWISFIFAQIFVEVPWNILAGTIAYFIYYYPIGFYSNASAAGQLHERGALFWLFSCAFYVYVGSMGLLVISFNQVAESAANLASLLFTMSLSFCGVMTTPSAMPRFWIFMYRVSPLTYFIQALLAVGVANVDVKCADYELLEFTPPSGMTCGQYMEPYLQLAKTGYLTDENATDTCSFCQISTTNDYLANVNSFYSERWRNYGIFICYIAFNYIAGVFFYWLARVPKKNGKLSKK

>CkABC1 [ABY55548.1](https://www.ncbi.nlm.nih.gov/protein/ABY55548.1?report=genbank&log$=protalign&blast_rank=2&RID=DDRPY4WZ014) 1521 aa

METISVGKESDVSSDGNVAYGGFNEGTNKQIRDLAREFTRQTSVGDGVQDSDDATDSNEVEKFGVDHASPNSPPKSNQYDLLRTLTSMSQVPGVNPVDQTIDPRLDPNSDEFESKFWVKNMRKLLDSDPDYYRPTSLGFAAKNLIAKGISSDADYQANFLNYPFKVVRDTYMDLFRGNDESRYFEILKSMDVLIKPGTLTVVLGRPGAGCSTFLKTVAAQTYGFKVDDSSIISYDGLTPKEINKNYRGEVIFSAEMDNHFPHLSVGQTLEFAAKMRTPQNRFPGVSRNEYAKHMSEVYMATYGLSHTVNTKVGDNFIRGVSGGERKRVSIAEASLCGANLQCWDNATRGLDAATALEFVRALKTSAHILDTTPLIAIYQCSQDAYDLFDNVVLLYEGYQIYFGPGDRAKDFFERMGYECPDRQTTADFLTSITSPAERVAKKGWENKVPQTPKEFSDYWRASAEYKELVADIDEYLSHCHNNNTREEFAEAHAIKQANHARPSSSFRVSYWMQIKLIAQRNIWRTKGDPSIMMFSVIANIIMGLIISSLFYNLSATTGTFYYRSAAMFFAVLFNAFSSLLEVMSLFESRPIVEKHKMFALYHPSADAFASIFTELPAKIATSLGFNLMFYFMVNFRRNPGRFFFYLLMNFMATLVMSHIFRSIGSCFKTLSESMPPATVFLTAMVIYTGFALPTPSMHGWSRWINYLDPVAYVFEALMANEFDGRRFECSQFIPSYPNADLANQVCSVVASVPGFSYVNGTDYIYESYRYKITHKWRNFGIVVGFIIFFLFVYVTLVELNKGAMQKGEIILFQQSKLREMRKEKKSKQISDIEGGSEKPAGVYDHGNEDSEDGVNNLTVGSDIFHWRDVCYEVQIKDETRRILNHVDGWVKPGTLTALMGASGAGKTTLLDVLANRVTMGVVSGSMFVNGRLRDQSFQRSTGYVQQQDLHLQTSTVREALRFSAYLRQSRTISKKEKDEYVESIIDILEMRSYADAVVGVAGEGLNVEQRKRLTIGVELAAKPKLLLFLDEPTSGLDSQTAWSVCQLMRKLADHGQAILCTIHQPSALLLKEFDRLLFLAKGGRTVYFGDLGENCQTLINYFESHGAHPCPAEANPAEWMLEVIGAAPGSHANQDYHEVWMSSDERRAVQEELHRMETELLQIPVDDSAEAKRSFASSYLIQYICVTKRVLQQYYRTPQYIWSKLFLAGANSIFNGFSFYRAGTSLQGLQNQMLSIFMLSVMLNTLVQQMLPLYITQRSIYEVRERPSKTFSWWVFLAAQVTAEFPWNLICGTISYFCWYYPIGLQNNASVTHTTAERGALTWLLIVGFFNYASSLGLMCIAGVEQEQNGANISNLLFTMCLNFCGILKYPTGFWKFMYRANPFTFWIASVLGAGVGDTPLVCSSKEIVYFAPPKGETCTTYIQPYIDEAGGYLVDSEREGYCGFCTASNTNAYLKSVHVEYSKRWQNWGIFICFIAINNIFMFLFYWLARVPKKDNRVKDASALKSDEKATLGPQTTSEA

>CaCDR1 [P43071.1](https://www.ncbi.nlm.nih.gov/protein/P43071.1?report=genbank&log$=protalign&blast_rank=1&RID=DDRWEYSV016) 1501 aa

MSDSKMSSQDESKLEKAISQDSSSENHSINEYHGFDAHTSENIQNLARTFTHDSFKDDSSAGLLKYLTHMSEVPGVNPYEHEEINNDQLNPDSENFNAKFWVKNLRKLFESDPEYYKPSKLGIGYRNLRAYGVANDSDYQPTVTNALWKLATEGFRHFQKDDDSRYFDILKSMDAIMRPGELTVVLGRPGAGCSTLLKTIAVNTYGFHIGKESQITYDGLSPHDIERHYRGDVIYSAETDVHFPHLSVGDTLEFAARLRTPQNRGEGIDRETYAKHMASVYMATYGLSHTRNTNVGNDFVRGVSGGERKRVSIAEASLSGANIQCWDNATRGLDSATALEFIRALKTSAVILDTTPLIAIYQCSQDAYDLFDKVVVLYEGYQIFFGKATKAKEYFEKMGWKCPQRQTTADFLTSLTNPAEREPLPGYEDKVPRTAQEFETYWKNSPEYAELTKEIDEYFVECERSNTRETYRESHVAKQSNNTRPASPYTVSFFMQVRYGVARNFLRMKGDPSIPIFSVFGQLVMGLILSSVFYNLSQTTGSFYYRGAAMFFAVLFNAFSSLLEIMSLFEARPIVEKHKKYALYRPSADALASIISELPVKLAMSMSFNFVFYFMVNFRRNPGRFFFYWLMCIWCTFVMSHLFRSIGAVSTSISGAMTPATVLLLAMVIYTGFVIPTPSMLGWSRWINYINPVGYVFESLMVNEFHGREFQCAQYVPSGPGYENISRSNQVCTAVGSVPGNEMVSGTNYLAGAYQYYNSHKWRNLGITIGFAVFFLAIYIALTEFNKGAMQKGEIVLFLKGSLKKHKRKTAASNKGDIEAGPVAGKLDYQDEAEAVNNEKFTEKGSTGSVDFPENREIFFWRDLTYQVKIKKEDRVILDHVDGWVKPGQITALMGASGAGKTTLLNCLSERVTTGIITDGERLVNGHALDSSFQRSIGYVQQQDVHLPTSTVREALQFSAYLRQSNKISKKEKDDYVDYVIDLLEMTDYADALVGVAGEGLNVEQRKRLTIGVELVAKPKLLLFLDEPTSGLDSQTAWSICKLMRKLADHGQAILCTIHQPSALIMAEFDRLLFLQKGGRTAYFGELGENCQTMINYFEKYGADPCPKEANPAEWMLQVVGAAPGSHAKQDYFEVWRNSSEYQAVREEINRMEAELSKLPRDNDPEALLKYAAPLWKQYLLVSWRTIVQDWRSPGYIYSKIFLVVSAALFNGFSFFKAKNNMQGLQNQMFSVFMFFIPFNTLVQQMLPYFVKQRDVYEVREAPSRTFSWFAFIAGQITSEIPYQVAVGTIAFFCWYYPLGLYNNATPTDSVNPRGVLMWMLVTAFYVYTATMGQLCMSFSELADNAANLATLLFTMCLNFCGVLAGPDVLPGFWIFMYRCNPFTYLVQAMLSTGLANTFVKCAEREYVSVKPPNGESCSTYLDPYIKFAGGYFETRNDGSCAFCQMSSTNTFLKSVNSLYSERWRNFGIFIAFIAINIILTVIFYWLARVPKGNREKKNKK

>Cne4098 [XP_012047327.1](https://www.ncbi.nlm.nih.gov/protein/XP_012047327.1?report=genbank&log$=protalign&blast_rank=1&RID=DDSCFBC1016) 1536 aa

MAFAGVGPMGPYDRTEHSAGAALNRTTSRSHYYDSHPNDNLPSPTDEYRNRELGQLARSWSRRSQTGAVGRGLSQAPQDESISEELSGDVFAYEQGSDLDPFSNNFDAKKWTKLMFHAHEATTPSRKAGLSFKNLGVFGYGSDADYQKTVGNLFLAGLGALRDLIGNRKRKVQILNGIDGVLEAGEMLVVLGPPGSGCSTMLKTIAGEMNGIYIDESSELNYRGITPKQMYGQFRGEAIYTAEVDVHFPNLTVGQTLSFAAEARAPRHTPNGISKKDYANHLRDVVMSIFGISHTLNTIVGNDFVRGVSGGERKRVTIAEAALAGAPLQCWDNSTRGLDSANAIEFCKNLRINADYMDISSVVAIYQAPQSAYDLFDKVSVLYEGEQIFFGKCNDAKQFFMDMGFHCPPQQTVPDFLTSLTSASERTPREGFEGKVPTTPQEFAAAWKKSDKYAELQEQIAQFEQKYPVNGENYNKFLESRRAQQSKHLRPKSPYTLSYGGQIKLCLRRGFQRLKADPSLTLTQLFGNFIMALIVGSVFYNMPENTSSFYSRGALLFFAILMSAFGSALEILILYAQRGIVEKHSRYAFYHPSAEAVASALTDIPYKVLNCICFNLALYFMANLRREPGPFFFFMLISFTLTMVMSMFFRSIASLSRSLTQALAPAAIMILALVIYTGFAINVQNMRGWARWINYLDPIAYGFESLMINEFHGREYACSAFVPTGPGYEGATGEERVCSTVGSVAGSPVVNGDAYINGSYEYYHAHKWRNFGILIGFFIFLTAVYLLATELITAKKSKGEILVFPRGKIPRALLAQSTVSHNSDDPEAGKYAGGGNVQKKVTGADRADAGIIQRQTAIFSWKDVVYDIKIKKEQRRILDHVDGWVKPGTLTALMGVSGAGKTTLLDVLATRVTMGVVTGEMLVDGQQRDISFQRKTGYVQQQDLHLETSTVREALRFSVLLRQPNYVSKKEKYEYVEEVLKLLEMDAYADAVVGVPGTGLNVEQRKRLTIGVELVAKPALLLFLDEPTSGLDSQTSWNILLLLRKLTEHGQAILCTIHQPSAMLFEQFDRLLFLAKGGKTVYFGEVGKESRTLINYFERNGAEKCPPGENPAEWMLSAIGASPGSHSTVDWHQTWLNSPDREEVRREIARIKETNGGKGKAAEQDKSREKSKAEIKAEYAEFAAPLWKQFIIVVWRVWQQHWRTPSYIWAKAALCIGSGLFIGFSFFKSGTSQQGLQNQLFSVFMLFTIFGQLVQQILPNFVTQRSLYEVRERPSKTYSWKVFIMSNVIAEIPWSILMGVIIYFTWYYPIGYYRNAIPTDSVHLRGALMFLYIEMFMLFTSTFAIMIVAGIDTAETAGNIANLLFLMCLIFCGVLATKDSFPHFWIFMYRVSPFTYLVEGMLGVAIANTNVVCADNELLSFNPPSGQTCGQYMSNYIAAAGGYLINEDATIGCSFCSVSKTNVFLAQFDIYYSHKWRDFGLLWIFVIFNAAAAVAIYYVARVPKNTGKEQASEPEEPEKNAAPGNTEKRSRRSESS

>PoanB2AEB1 [XP_001904000.1](https://www.ncbi.nlm.nih.gov/protein/XP_001904000.1?report=genbank&log$=protalign&blast_rank=1&RID=DDTHCGB9014) 1531 aa

MSFTGAGSFGNYDLTAQSTGAPLGRTTTNDQQREEVVTAGGSSPGRNENDVSRTRATSLTEVADSEAVIRNEKGNITDAADEDEEAIEEERRHSAVLALARKYTSQSHYGVEPGTNVFEAALQDENSPINPNGPNFNSKAWAKAVVSMMDGRGASFRTSGVAFQHLNVFGFGAPTDYQKDVANVWLELVGLARKLTGNKGRRIDILRDFDGVVEKGEMLVVLGPPGSGCSTFLKTIAGDYNGIYMDENSYFNYQGMTAKEMHTHHRGEAIYTAEVDTHFPQLSVGDTLTFAARARAPRQLPPGVSKNMFAQHLRDVVMAMFGISHTVNTRVGNEYIRGVSGGERKRVTIAEAALSGAPLQCWDNSTRGLDSANAIEFCKNLKMSSDLFQSTCCVSIYQAPQSAYDLFDKALVLYEGRQIFFGKASEARQYFERLGFDCPSRQTTPDFLTSMTSPLERVVRPGWEDKAPRTPDEFAAAWKKSPEYQALQAQIEAYKASHPINGPDAEAFRASKQAQQAKSQRVKSPFTLSYMQQIQLCLWRGWKRLTGDPSLSIGALVGNTIMALIISSIFYNLQPTTDSFYQRGALLFFACLMNAFSSALEILTLYSQRPIVEKHKAYALYHPSAEAIASMLCDLPYKIANTLVFNLTLYFMTNLRREAGAFFFFLLFSFFTVLVMSMIFRTIASSTRTLSQAMVPAAAIILALVIFTGFVIPIDYMPGWCRWINYIDPLAYSFESLMVNEFHGRNFTCTQFVPNLLIPGYGDISPANRACSAIGSIAGSSVVNGDDYINSAFKYYVSHKWRNFGILLAFIAFFTTTYMLAAETVSAAKSKGEVLLFRRGHKPASFKENKGDAESGGVAVAGPVAKAAAGYQSDKESGNIQGSTSVFHWNNVCYEVKVKKETRQILNNVDGWVKPGTLTALMGVSGAGKTTLLDCLADRTSMGVITGEMLVDGLPRDASFQRKTGYVQQQDLHLQTTTVREALNFSALLRQPAHVPREEKLAYVDEVIKLLEMEEYADAIIGVPGEGLNVEQRKRLTIGVELAAKPPLLLFVDEPTSGLDSQTSWAILDLLEKLTKSGQAILCTIHQPSAMLFQRFDRLLFLAKGGRTVYFGDIGENSKTMTSYFERNGGFPCPADANPAEWMLEVIGAAPGSVTNVDWHQAWRESPEYAAVQEELQRLKAQAKPSDALATDDGSYREFAAPFGEQLRSVTHRVFQQYWRTPTYIYSKAILCLIVSLFIGFVFFKAPNTIQGLQNQMFAIFNILTVFGQLVQQTMPHFVVQRSLYEVRERPSKVYSWKVFMLSQIIVELPWNTLMAALMFVTWYYPVGLDANAAAAGQTAERGALMFLLLVAFMLFTSTFTDFIIAGFETAEAGGNIANLLFSLCLIFCGVLATPETMPRFWIFMYRVSPFTYLVSAMLSTAVANSEVVCAANELQKFAPPSGQTCFEYLESYMEIAGGYLTNPNSTDMCSFCTIKDTNVFLAQVGANYDDRWRNFGILWAFIIFNIFAALGVYWLVRVPKKKLGAKAKKE

>FvQ3ZZY6 [CAI60778.2](https://www.ncbi.nlm.nih.gov/protein/CAI60778.2?report=genbank&log$=protalign&blast_rank=1&RID=DDTBYB03014) 1500 aa

MASTYMGGTSVQPAKLPGLGPNLTANNQTTTSTHDPQTPVNPSSDTTPTNYSQDHMASKDETPAKSTTEDEEDSELERRHSIVRDLARQYTNQSQMSAISGNPFTADENSPLNPRSDKFRALAWAKAISKLHSGTGFINRKAGVCYQNLNVFGYGQPTDYQKNVANIWLDVASLPRQLMGYGKTRIDIIRDFDGVVKNGEMLVVLGPPGSGCSTYLKTISGETSGIYINDDAYFNYRGITAHEMHTRHRGEAIYTAEVDVHFPHLTVGDTLDFAAHARAPRSIPGGIDRETFISHSRDVVMAMFGISHTVNTKVGNEYVRGVSGGERKRVTIAEASLSGAPLQCWDNSTRGLDSANAVEFCKTLRLQTQMSDTAAMVSIYQAPQAAYDIFDKVVVLYEGRQIFFGGTKAAKRYFEDLGFECPARQTVPDFLTSMTSPQERIIRKGFEDRAPRTPDEFAAAWKASQANILLQQEIEEYKRDFPINGPEAEEFRANRRAAQAKNQRKKSPYTLSYWQQTKLCVWRGFKRLVTDPTITLTQLFGNFIMALIVGSVFYNINQTTSSFFQRGALLFLACLSNAFSSALEILTLYAQRPIVEKHDRYALYHPSSEAVASMLCDMPYKLNAFTYNLTLYFMTNLRREPGPFFFFLFMTFLVTLCMSMIFRTIASASRTLSQAMVPAALLILALVTFTGFVIQIDYMLGWCRWINYVNPLAYAFESLMVNEFHNRKFECNLFIPTYPDAAPENRVCSTVGSVQGEPMVSGDRYINLSFSYYHAHKWRNVGIVIAFTLLFLVTYMFFAEAVSAKKSKGEVLVFRRGHRLAKNKADAESSPAGRVAITEKEGYGEGQPSNFKSTSVFHWNNVCYDVKIKSENRRILDNVAGWVKPGTMTALMGVSGAGKTTLLDCLADRTSMGVIHGDILVDDKLRDASFQRKTGYVQQQDLHLSTTTVREALNFSAIMRQPKHIPVKQKIAYVDEVIKMLDMQDYSEAVVGILGEGLNVEQRKRLTIGVELAAKPPVLLFVDEPTSGLDSQTSWAILDLLEKLARSGQAILCTIHQPSAMLFQRFDRLLFLASGGKTVYFGDVGDHSRTMTRYFESHGAEPCPPEANPAEWMLEVIGAAPGSETNLDWPQIWKDSSEFAEVQKHLKELSHHQVEKSQDEDPHLYDEFAATFTTQLKYVTARVFEQYWRTPSYIYSKAALCTLVALFIGFSFYQAPNTALGLQNQMFAIFQVLTVFGQLTQQQMPHFVIQRDLYEVRERPSKTYSWQVFILSQIIVEIPWNTLMAAIMYFCWYYPIGLYRNAEPTDAVAERGALMFLLFLVFMLFTCTFTDFIIAGCSSAETGGNIANLLFMMCLIFCGVLATPDSFPKFWIWMYRVSPFTYLISAILSTAVANTEVVCAQNEYVVFPPPKGQICGEYMQAYIKRAGGYLVDESSNSTCTYCTIGDSNVFLAGVRSHYDERWRNFGLMWVYIIFNVFAALALYWLVRVPKNKKGEKEKKE

>BociBMR3 [BAC67160.1](https://www.ncbi.nlm.nih.gov/protein/BAC67160.1?report=genbank&log$=protalign&blast_rank=1&RID=DDTY49NX014) 1448 aa

MERLEHMSWRNKTPCMGLSWGTQHWTPTPQILTYINGLKCKFWHVQVFIWTQTNCTCRMMRLVDENGVIQRRAGIVFKNLKVCGSGSAINVQKNVGSLLMAPLRFKEFIGKGPEKTILNDFNGVLKSGEMLIVLGRPGSGCSTFLKSLMGELYGLDMKAQSEIHYNGITQKQMLKQFRGEIVYNQEVDKHFPHLTVGETLEFAASVRTPQQRLVEGTTRSAWAKHMTKVVMAIYGLSHTYNTKVGNDFVRGVSGGERKRVSIAEMALAGSPIASWDNATRGLDAATALEFTKSLRMTANLSGSCHLVAIYQASQQIYDQFDKAIVLYEGRQIYYGPCDQAKQYFEDMGWECPSRQTTGDFLTSITNPSERKARPGYENKVPRTPEEFEKYFKDSKIFQRMMSEMKSHEEEFPMGRKTLEQFKASRKGMQADHLRPESPYTVSIVMQTKLCARRAVQRLWNDKTSTITTIVGQIAMALIIGSIFYNTPSNTASFFQKGGVLFFAVLLNALIAISEINTLYSQRPIVEKQASYAFYHPFTEALAGVVVDIPVKFAIATCFNIILYFLSGLKREAGAFFVFFLFNFVAILTMSQIYRSIAAATKTISQALAIAGVATLAIVIYTGFVIPRPLMHPWFKWISWINPVAYAFEALFVNELHGKEFVCSTLVPTGPGYVQAGNNFVCAVAGSVVGATTVSGDDYLQAQFQYSYSHIWRNLGFLFAFMIFFLAFYLLATEFNASTDSKAEVLVFRRGHVPTNLLAAEKAAKNDEEAHAGNGSAVKEGNSDKQGDEVQALAPQTDIFTWKDVCYDIKIKNEPRRLLDNVSGWVKPGTLTALMGVSGAGKTTLLDVLAQRVSMGVITGDMLVSGKPLDASFQRKTGYVQQQDLHLETTTVREALRFSAMLRQPKTVSKKEKYDFVEEVIKMLNMEEFSEAVVGVPGEGLNVEQRKLLTIGVELAAKPALLLFLDEPTSGLDSQSSWAIVSFLRKLADNGQAVLATIHQPSAILFQEFDRLLFLAKGGRTVYFGDIGHNSETLLNYFESHGAAKCGEDENPAEYMLTMVGAGAQGKSTQDWHEVWKASDEAKGIQTEISRIQQEMGHQPSQDDSNSHGEFAMPFTVQLLEVMKRVFQQYWRTPGYVYSKLVLGVASALFIGFSFFHADASQQGLQDVIFSIFMITTIFTTLVQQIMPRFILQRDLYEVRERPSKAYSWKAFIIANIAVEIPYQIILGIMVFASYFYPIYTKNGIPPSGRQGLILLLLIQFFVFASTFAHMLISALPDAETAGNIATLMFSLTLTFNGVFQPPQALPGFWIFMYRVSPLTYLVSAIASTGLSGRQVICSDNELAVMQPPAGDTCGSYLQSYATAAGGSIYNPEAMADCQYCSSSNADQFLSSVAISYTTRWRDYGIVFVYIFFNIFMAVLLYYLIRVRKSSGKSLKEKFGALGALFKKN

>AfuQ4WWW3 [XP_754878.1](https://www.ncbi.nlm.nih.gov/protein/XP_754878.1?report=genbank&log$=protalign&blast_rank=1&RID=DDTNUTD9014) 1472 aa

MRRSNVVPVHSLTSSTNTGRDSRGEKYDELTPVATRRASISPDEARYLTQLASRDNAVSRVSTVADISLDDPALNPENKDFDLYKWLRKVVHVLNEEGVPRKEASIFFQHLRVSGTGAALQLQKTVADIITAPFRRETWNFRNKTSKTILHDFNGMLHSGELLIVLGRPGSGCSTFLKTLSGELHGLNVDEKTVLHYSGIPQSTMIKEFKGEVVYNQEVDKHFPHLTVGQTLEFAAAVRTPSKRLGGMSRNEYAQMMTKVVMAVFGLSHTYNTKVGNDTVRGVPGGERKRVSIAEMALAGAPLAAWDNSTRGLDSATALKFVESLRLAADLNSSAHAVAIYQASQAIYDLFDKAVVLYEGRQIYFGPASKAKAFFERQGWFCPPRQTTGDFLTSVTNPIERQARPGMESQVPRTAAEFEAYWLESEEYKELQREMAAFQGETSSQGNEKLLEFQQRKRLAQASHTRPKSPYLLSIPMQIKLNTKRAYQRVWNERTSTMTTFIGNTILALIVGSVFYGTPTATAGFYAKGATLFYAVLLNALTAMTEINSLYSQRPIVEKHASFAFYHPATEAIAGVVSDIPVKFLMAIAFNIILYFLSGLRREPSQFFIYFLITFIIMFVMSAVFRTMAAITRTVSQAMTLAGVLILMLVIYTGFVVPVNYMHPWFKWIHYLNPIFYAFEILIANEFHGREFTCSQFIPVYPNLPGDSFVCSSRGAVAGRRTVSGDAYIEASYSYSYSHVWRNFGILIAFLIGFMVIYFVATELNSATTSSAEVLVFRRGHEPAHLKNGHEPGADEEAGAGKTVVSSSAEENKQDQGITSIPPQQDIFTWRDVVYDIEIKGEPRRLLDHVSGWVKPGTLTALMGVSGAGKTTLLDVLAHRTTMGVITGDMFVNGKPLDSSFQRKTGYVQQQDLHLETATVRESLRFSAMLRQPASVSKEEKYAYVEEVIKMLNMEDFAEAVVGVPGEGLNVEQRKLLTIGVELAAKPKLLLFLDEPTSGLDSQSSWAICNFLRKLADAGQAILCTIHQPSAILFEQFDQLLFLARGGKTVYFGPIGENSQTLLKYFESHGPRRCGDQENPAEYMLEVVNAGTNPRGENWFDLWKASKEAAGVQAEIDRIHESKRGEAESKDSTNPKDREHEEFAMPFFKQLPIVTVRVFQQYWRLPMYIAAKMMLGICAGLFIGFSFFKADTSLQGMQNVIFSVFMLCAIFSSLVQQIIPLFITQRALYEVRERPSKTYSWKAFMIANIIVEIPYQILMGILVFGCYYYAVNGVQSSDRQGLVLLFCIQFFIYASTFADFVIAALPDAETAGAIVTLQFSMALTFNGVMQTPEALPGFWIFMYRVSPFTYWVGGMAATQLHGRAVKCSAAETAIFNPPSGLTCQEYMADYMAVAPGHLSNPNATSSCEFCSLSVADQYLASVNIYWSERWRNFGIFWAYVVFDIAVAVMLYYCFRVKKWNFSFGKRKKSKAA

>PechB6HED3 [XP_002563049.1](https://www.ncbi.nlm.nih.gov/protein/XP_002563049.1?report=genbank&log$=protalign&blast_rank=1&RID=DDTTCNEC014) 1472 aa

MTSETSSFELREKDEQSPPHTGDASTLGDSPMELIDQVDVETLTKIATARSRRQSTLGTTDNLAVLAQQDPALDPQSGKFDLRKWLKAAFNDINREGHSGHTSDVVFKQLNVYGSGAALQFQDTVTSTLTAPFRLPQIIRESKSPQRRILKDFNGLLKSGELLLVLGRPGAGCSTLLKSMTGELHGLNLDKDSVIHYNGIPQSRMIKEFKGELVYNQEVDRHFPHLTVGQTLEFAAATRTPSHRFQGMSRAEFAKYVAQITMAVFGLSHTYNTRVGDDFIRGVSGGERKRVSIAEMAVAHAPIAAWDNSTRGLDSATALKFVEALRLSSDITGSCHAVAAYQASQSIYDIFDKVIVLYEGHQIFFGPAAAAKSYFERQGWACPARQTTGDFLTSITNPQERQTKPGMENRVPRTPEDFETAWLKSPEYKQLLNETAEYEGKNPIGKDVQALADFQQWKRGVQAKHTRPKSPYIISVPMQIKLNTIRAYQRLWNDAASTISVVVTNIIMALIIGSVFYGTPDATAGFTSKGATLFFAVLLNALTAMSEINSLYSQRPIVEKHASFAFYHPATEAIAGVISDIPVKFALSVVFNIILYFLAGLKREASNFFLYFLITFIITFVMSAIFRTLAAVTKTISQAMGLAGVMILVLVVYTGFVLPVPSMHPWFEWIHYLNPIYYAFEILIANEFHGREFPCSSYVPSYADLSGHAFSCTAAGSEAGSRTVSGDRYIQLNYDYSYSHVWRNFGILIAFLIGFMIIYFVASELNSATTSTAEALVFRRGHEPASFRQDHKSGSDVESTKLSQAQPAAGTEDKGMGAIQPQTDTFTWRDVSYDIEIKGEPRRLLDNVSGWVKPGTLTALMGVSGAGKTTLLDVLAHRTSMGVITGDMFVNGHGLDQSFQRKTGYVQQQDLHLDTATVRESLRFSAMLRQPASVSVKEKYDYVEDVIKMLKMEEFAEAIVGVPGEGLNVEQRKLLTIGVELAAKPKLLLFLDEPTRQSSWAICSFLRKLAEHGQAVLCTIHQPSAMLFQQFDQLLFLARGGKTVYFGPVGENSSTMLEYFESNGARKCADDENPAEYMLGIVNAGQNNKGQDWYDVWKQSDESKQVQTEIDRIHKEKEHQPPSADDSAQSHSEFAMPFMFQLSQVTYRVFQQYWRMPSYILAKWGLGIVSGLFIGFSFYSAKTSLQGMQTVIYSLFMICTIFSSLAQQIMPVFVSQRSLYEGRERPSKSYSWKAFLIANIIVEIPFMVVMGVLTYASYFYAVVGVPSSLTQGTVLLFCIIFFIYASTFTHMVIAGLPDEQTASAVVVLLFAMSLTFCGVMQPPSALPGFWIFMYRVSPFTYWVGGMASTQLHNRQVVCSAAELAVFDPPSGQTCGQYLMQYAAAAGGKLLNPEATSDCSYCSLEVADQYLSTANIFYSERWRNFGIMWAFIGFNIFVATIMYYLVRVKRWSSADLKESVMKLIPGKKSKGGN

>ScPDR12 1511 aa

MSSTDEHIEKDISSRSNHDDDYANSVQSYAASEGQVDNEDLAATSQLSRHLSNILSNEEGIERLESMARVISHKTKKEMDSFEINDLDFDLRSLLHYLRSRQLEQGIEPGDSGIAFKNLTAVGVDASAAYGPSVEEMFRNIASIPAHLISKFTKKSDVPLRNIIQNCTGVVESGEMLFVVGRPGAGCSTFLKCLSGETSELVDVQGEFSYDGLDQSEMMSKYKGYVIYCPELDFHFPKITVKETIDFALKCKTPRVRIDKMTRKQYVDNIRDMWCTVFGLRHTYATKVGNDFVRGVSGGERKRVSLVEAQAMNASIYSWDNATRGLDASTALEFAQAIRTATNMVNNSAIVAIYQAGENIYELFDKTTVLYNGRQIYFGPADKAVGYFQRMGWVKPNRMTSAEFLTSVTVDFENRTLDIKPGYEDKVPKSSSEFEEYWLNSEDYQELLRTYDDYQSRHPVNETRDRLDVAKKQRLQQGQRENSQYVVNYWTQVYYCMIRGFQRVKGDSTYTKVYLSSFLIKALIIGSMFHKIDDKSQSTTAGAYSRGGMLFYVLLFASVTSLAEIGNSFSSRPVIVKHKSYSMYHLSAESLQEIITEFPTKFVAIVILCLITYWIPFMKYEAGAFFQYILYLLTVQQCTSFIFKFVATMSKSGVDAHAVGGLWVLMLCVYAGFVLPIGEMHHWIRWLHFINPLTYAFESLVSTEFHHREMLCSALVPSGPGYEGISIANQVCDAAGAVKGNLYVSGDSYILHQYHFAYKHAWRNWGVNIVWTFGYIVFNVILSEYLKPVEGGGDLLLYKRGHMPELGTENADARTASREEMMEALNGPNVDLEKVIAEKDVFTWNHLDYTIPYDGATRKLLSDVFGYVKPGKMTALMGESGAGKTTLLNVLAQRINMGVITGDMLVNAKPLPASFNRSCGYVAQADNHMAELSVRESLRFAAELRQQSSVPLEEKYEYVEKIITLLGMQNYAEALVGKTGRGLNVEQRKKLSIGVELVAKPSLLLFLDEPTSGLDSQSAWSIVQFMRALADSGQSILCTIHQPSATLFEQFDRLLLLKKGGKMVYFGDIGPNSETLLKYFERQSGMKCGVSENPAEYILNCIGAGATASVNSDWHDLWLASPECAAARAEVEELHRTLPGRAVNDDPELATRFAASYMTQIKCVLRRTALQFWRSPVYIRAKFFECVACALFVGLSYVGVNHSVGGAIEAFSSIFMLLLIALAMINQLHVFAYDSRELYEVREAASNTFHWSVLLLCHAAVENFWSTLCQFMCFICYYWPAQFSGRASHAGFFFFFYVLIFPLYFVTYGLWILYMSPDVPSASMINSNLFAAMLLFCGILQPREKMPAFWRRLMYNVSPFTYVVQALVTPLVHNKKVVCNPHEYNIMDPPSGKTCGEFLSTYMDNNTGYLVNPTATENCQYCPYTVQDQVVAKYNVKWDHRWRNFGFMWAYICFNIAAMLICYYVVRVKVWSLKSVLNFKKWFNGPRKERHEKDTNIFQTVPGDENKITKK

>ScSNQ2 [XP_002491174.1](https://www.ncbi.nlm.nih.gov/protein/XP_002491174.1?report=genbank&log$=protalign&blast_rank=1&RID=DDU8FCC3016) 1501 aa

MSNIKSTQDSSHNAVARSSSASFAASEESFTGITHDKDEQSDTPADKLTKMLTGPARDTASQISATVSEMAPDVVSKVESFADALSRHTTRSGAFNMDSDSDDGFDAHAIFESFVRDADEQGIHIRKAGVTIEDVSAKGVDASALEGATFGNILCLPLTIFKGIKAKRHQKMRQIISNVNALAEAGEMILVLGRPGAGCSSFLKVTAGEIDQFAGGVSGEVAYDGIPQEEMMKRYKADVIYNGELDVHFPYLTVKQTLDFAIACKTPALRVNNVSKKEYIASRRDLYATIFGLRHTYNTKVGNDFVRGVSGGERKRVSIAEALAAKGSIYCWDNATRGLDASTALEYAKAIRIMTNLLKSTAFVTIYQASENIYETFDKVTVLYSGKQIYFGLIHEAKPYFAKMGYLCPPRQATAEFLTALTDPNGFHLIKPGYENKVPRTAEEFETYWLNSPEFAQMKKDIAAYKEKVNTEKTKEVYDESMAQEKSKYTRKKSYYTVSYWEQVKLCTQRGFQRIYGNKSYTVINVCSAIIQSFITGSLFYNTPSSTSGAFSRGGVLYFALLYYSLMGLANISFEHRPILQKHKGYSLYHPSAEAIGSTLASFPFRMIGLTCFFIILFFLSGLHRTAGSFFTIYLFLTMCSEAINGLFEMVSSVCDTLSQANSISGILMMSISMYSTYMIQLPSMHPWFKWISYVLPIRYAFESMLNAEFHGRHMDCANTLVPSGGDYDNLSDDYKVCAFVGSKPGQSYVLGDDYLKNQFQYVYKHTWRNFGILWCFLLGYVVLKVIFTEYKRPVKGGGDALIFKKGSKRFIAHADEESPDNVNDIDAKEQFSSESSGANDEVFDDLEAKGVFIWKDVCFTIPYEGGKRMLLDNVSGYCIPGTMTALMGESGAGKTTLLNTLAQRNVGIITGDMLVNGRPIDASFERRTGYVQQQDIHIAELTVRESLQFSARMRRPQHLPDSEKMDYVEKIIRVLGMEEYAEALVGEVGCGLNVEQRKKLSIGVELVAKPDLLLFLDEPTSGLDSQSSWAIIQLLRKLSKAGQSILCTIHQPSATLFEEFDRLLLLRKGGQTVYFGDIGKNSATILNYFERNGARKCDSSENPAEYILEAIGAGATASVKEDWHEKWLNSVEFEQTKEKVQDLINDLSKQETKSEVGDKPSKYATSYAYQFRYVLIRTSTSFWRSLNYIMSKMMLMLVGGLYIGFTFFNVGKSYVGLQNAMFAAFISIILSAPAMNQIQGRAIASRELFEVRESQSNMFHWSLVLITQYLSELPYHLFFSTIFFVSSYFPLRIFFEASRSAVYFLNYCIMFQLYYVGLGLMILYMSPNLPSANVILGLCLSFMLSFCGVTQPVSLMPGFWTFMWKASPYTYFVQNLVGIMLHKKPVVCKKKELNYFNPPNGSTCGEYMKPFLEKATGYIENPDATSDCAYCIYEVGDNYLTHISSKYSYLWRNFGIFWIYIFFNIIAMVCVYYLFHVRQSSFLSPVSILNKIKNIRKKKQ

>CaSNQ2 [KGQ86254.1](https://www.ncbi.nlm.nih.gov/protein/KGQ86254.1?report=genbank&log$=protalign&blast_rank=1&RID=DDUDN6FH014) 1495 aa

MSSEDIGSSSSSLQEYVGQQQHNKIQPSTDDDYNEDDYESRRLHLVRTVSSINHHNFDEKFDTISREISRQVTNKEGEFQLRLDEFNLAKILANFVYFAKKQGIVLRKSGITFQDLCVYGVDESFAIAPTVTDLLKGPVGAVQAILSQMKTPPRKILKNLNGFAKPGESVLVLGRPGAGCTTFLKALSGTDFDLYKGVTGDIRYDGLPQKEMLKLFKNDLVYNPELDVHFPHLTVDQTLTFAIACKTPEMRINGVTRDEFINAKKEILATVFGLRHTYHTKVGNDFVRGVSGGERKRVSIAEALACNGSIYCWDNATRGLDASTALEFAQAIRTSTKLLKTTAFVTIYQAGEGIYETFDRVTVLYDGHQVYYGPANKAKKYFEDMGWECPPRQSTAEFLTAITDPIGRFPRAGWENKVPRTAQDFEHYWLNSPQYQELMQEIKDYNDEIDEDETRSKYYQSIQQEKMKGSRTKSPFTISYLEQLKLCFIRSYQRILGDSAYTITLMFASVAQAFVAGSLYYNTPDDVSGAFSRGGVIFFAVLFMSLMGLAEISASFSSRPILMKQKNYTMYHPSADSLSNFVMSIPISIFINTFFVIILYFLSNLARDAGKFFICYLFVIMLHLTMKSMFQAIAAINKSIAGANAMGGILMLASLMYSSYMIQRPSMHPWFKWISYINPVLYAFEAVIASEFHGRKMQCTSQYLTPSGPGYENLGAGEQVCTFIGSVPGQSWVLGDDYLRIAYTYRFSHVWRNLGILFGFLAFFLAIATLGTEYVKPITGGGDKLLFLKGKVPEHITLPSEKKEEDIESGGNSDTTATSNGTLSQGKSEEKAAIADDGLKAKGVFVWKDVDYVIPYEGKKRQLLQNVSGYCVPGTLTALMGESGAGKTTLLNVLAQRVDFGVITGDMLVNGRPLDTSFSRRTGYVQQQDIHFSEVTVRESLQFAARLRRSNDVSDAEKLEYVEKIIDVLDMRGYADAVVGRLGNGLNVEQRKKLSIGVELVAKPSLLLFLDEPTSGLDSQSAWAIVKLLRDLANAGQSILCTIHQPSATLFEEFDRLLLLKKGGIVTYFGDIGPRSRTILDYFERNGARHCDDKENPAEYILEAIGAGATASTDFDWGEIWAQSPEKVQTDAKRDELINESAKNATDTSATDSPSEKNLTSKYATPYWYQFRHVTHRTSLIFYRDPDYIAAKVFLMTIAGLFIGFTFFGLKHTKTGAQNGMFCAFLSCVIAAPLINQMLEKAGSRDIYEVREKLSNTYHWSLLILPQIIFEVIYMIIGGTIMFVCLYFPTQVSTVASHSGMFYFSQAIFLQTFAVSFGLMVSYVSPDIESASVIVSFLYTFIVSFSGVVQPVNLMPGFWTFMNKVSPYTYFIQNLVSSFLHDRTIRCNAKELSYFNPPSGQTCKEFASAFISRNGGYLVDEGATSNCGYCNFSNADQYLLTIGAKFSYRWRNIGFFCVYIIFNISVCLVLYYFLRYRKVSFNVTGLVNKFKKSKK

>AfuYOL075C 1299 aa

MEKDDDLIAELKAPEDLVFADGLQDQTATFNLSLRAVDPVDVCVENLSLQVDTTRPIWKTSPSQLWNRLCGKTMDTHTHKTVLDSVNAFMPSGSLTAIIGSSGSGKTSLLNIMAGRMSLTKAKVSGATTFNGVAGIEGIRSAYVMQEDVLIPTLTVRETLRYAADLRLPSPATQEERHQVVEQVVLELGLKECADTRIGTNTHKGCSGGEKRRTSIGVQMLANPSVLFCDEPTTGLDATSAFQIIRTLKRLAEDGRTVIVSIHAPRSEIWSLFDNVILLARGSVLYSGSRQDSLSHFETCGHVLPPFVNPAEFLIDLAAIDNRTESLEAASMARVELLKAAWKSRSSERKQIEQSRHKGKMSTPSGAFTTSPNKTASFRQQFRVLTSRTFTTTIRDPLGMAGSLLEAVGMAVINGWIFLQLDESQAGIRSRQGSLYTASSLNGYLILLYETYRLTIDIRLFDRERNEGVVGVPAFLLSRRAARLPLEDLPVPIIFAIIYYFMVGYRLSVAQFFVFLLLTILTHYIAVTFAAVSIGVARSFPGASLVGNLSFTLQSFACGYFVQSNQIPVYVRWLKWVAYTFYIFGALCANEFIGPDGPPEGQFYDCPYSTDPSNPACTQYTGRYIMENLGFPSNWIWRPIVILVAFVIGHYLLAGLLLQYNHFAIDIAQARKTDVDLSAGKEKFAERRSEEARPVAISLDKYALEIRKRQVSRRGSRTLFILKPITAEFQPGKLNVIMGPSGSGKTSLLNSIARRLRGSLGTQYRLQGNMLYNGAVPSESVIRSVTSFVTQDDDALMPSLTVRESLRFAAGLRLPQWMSREEKNQRAEEILLKMGLKECADNLIGSELIKGISGGEKRRVTIAIQILTDPKVLLLDEPTSGLDAFTATSIIEVLEALAAEGRTLIMTIHQSRSDLFQHFSRVLLLARGGYTVYAGEGEKMLPYFRSLGYECPKTTNPADFVLDLITVDLQQEDREALTRERVQKLITSWDGQQQDEGRRPSQIATPAELGSLKRRMLPFRITYPLVLHRAAINFWRQPPLVMARSLQVVGIAIIMALFFAPLKNDYAAVQSRMGFIQEFAALYFVGMLQNIAIYPNERDVFYREEADHCYSAETFILQYTTLEVPFEAISSIIFGVLAAYADNLERSPKMFLISAFNCFCIISCGESVGIMFCTLFSHVGFAVNVTSILLSISTILGGVMSLNVNDVLQAINHLSPIKYSIANLAPYAMRDQHFTCTASQLLANGTCPIQTGQQVLQLYNLDKNAPMNVMALGICTIIYRLVAYAMLKVMREGFWRRLR

>FgYOL075C XP_011325440 1351 aa 2 EL3 and 2 EL6 cysteines

MSQHGNPSDNIDIEKMDRPSSLEISIDPRVQQSEMSRLSVSGGGNLSLADVQAVHVHIHDLAVSVDTAPSWLAPSTYGDLVSSKFNTASKMKPLLHSVSANLPPGTLTAIIGGSGSGKTTLLNTVAERVLSSRLSQQGIATFNGRVGVHSVRHAYVMQQDILLPTLTVRETLRYSADLRLPPSTTSEERQRVVEEVILELGLKECADTRIGNSQHHGCSGGEKRRTSIGVQLLANPSVLFLDEPTTGLDATSAYQLVRTLKTLAQKGRTIITTIHQPRSEIWDLFDNLIVLTKGSPVYSGTIKESVPWFGELGYQLPPFINPAEFIIDIAAVDNRTPELEQETTAKVERLKSAWNQETLKRYPPPDKTVDIRDGKKKKDKKTEEHAGFLRQVTVLTDRTLKVTYRDPLGMAASITEAVFMGLVTGYMFYNLGRDQAGIRSRQGGLYTAAGLQGYLILIFEVYRMTFDIPTFDRENSEGCVDALPFVLSRRIARMITEDVAAPFLFSVLFFFMAGFERDVARFFTFFAITLLNQYIAVTCAMVCVATVRHFAGASVIANLVFTLQSMACGMFINVNSLPVYVRWLKWLTYTFYVFSAYCGNEFEGSFYDCPASNDRSDPRCKQYTGAYIMESLGFPKDWVAKPILVCLAFVVFFFVLSVIGLRIIKVEMTIARARVSDTDLSAGKEKMTARSVADVRTIDLELNEFSLALDKRTQLGKKLPTKTILNPVNATFSAGVLNVIMGPSGSGKTSLLNAMALRLRDSVGTKYRPAGKLTFNGALPSDSVIRSVCSYVCQDDDALLPSLTVRETLRFAAGLRLPSFMSKDEKNRRAEEVLLKMGLKDCADNLVGGELVKGISGGEKRRVSIAIQVLTDPRILLLDEPTSGLDAFTANSIMEVLQGLANEGRTLILTIHQARSDLFREFGNVLLLARGGSQVYSGPGRDMLGYLARHGYECPHHTNPADFALDIITIDLQHEGKELESRKRVQNMIDNWKAESASIKGEKLSDIQEKDEVQNTDSADQINTTQEGTTLPPAPPQKRRSFNKANLSTPAELGALIRKRASITTALPLLLHRALINTYRQPELIVARLMQVIGLALILALFFAPFDNDYYSVQSRMGFVQELGAFYFVGMLQNTAIYPGERDVFYREDDDGVYSVNAFLASYTILEVPFELISCLIFGVLAVIAVDLPRTATLYFTSVFACFGIVSCGESLGIMFNTLFGHTGFAVNIMGVFLALANTMAGVLSIGMPDLFKAFNYLSPIRYGTRAVAPYSLRGIEFTCNNEQRLENGKCPIETGQDVLELYSFDVDPVVNIACLAACVVVYRLLAWGLLKIARTHWKGKKKDKERVNKA

>YaliYOL075C [XP_503291.1](https://www.ncbi.nlm.nih.gov/protein/XP_503291.1?report=genbank&log$=protalign&blast_rank=1&RID=DDVD6KUN014) 1328 aa

MEKTVTAQVSRTSHNELSFQSVNPVSISVRGLSVTVKAEEQKGGFFSRRKKKTSATEKQTTAESISIDDKSAKKEKNKKSQKKKEKESDDVEPQARDILSNISLDIPAGSIMAILGGSGSGKTSLLNMMASRMSGGNLTVEGETLFDGKSIEHVTHAYVIQQDILSPHLTCRETLNFAAGLRLDKSINKVQRSELVEEVIKELNLKECADTMVGNSIHRGLSGGEKRRLSIGIQMLSNPSVLFLDEPTTGLDANSAFDLVKTMKNLSLSGRTLIMSIHQPRSDIFFLFDHVTILSRGLQVYSGSTKESINWFASLGYDCPRDVNPADYLIDIAAVDTRSEEDEEQSFKRINAFVDKYNELKIETGISDVSGTASKSSSTANLQTRLFRNSFKTALFSSAPLGREIDVQVRRTWLIMYRDKLGIVGLTVEAILMGLICGLVFLRMKPDLAGIRSMEGAAYIAISLQGYLMLLYETYRLCATDLAVFDREHNEGCASVFGFLIARRMAKLFTEDLIVPIIFSVLTYFLFGFRTDGAKYFFIYFAQILLTHHISMNFSMVCAALSRDYTIASLVANLMFTLQSMASGFFANSEHMKVYIRWTKWITYVFYGLSALLNNQFMNFFGDCPYGNKTADDPLCKDFIGENFLESVGFPRNFLVIPTIALLCWLIVFFLLSWLLLTIIRVDVGVGKNLKGDSNKAEKPEAAEKVTTIASVNRNPPLTVAVSDLKLSVTKLRAKEKPILDGINAIFRPGSISAILGPSGSGKSSLLNLMANRLNSTLTQKYTASGDIFLNSTSIGIGNLGALCSFVTQEDDGLLSTLTVRETLYFSAYLRLPDNLTREMKRRRADELILKMGLKDCQDTLIGDDNVKGISGGEKRRVSICVQLLSNPDILLLDEPTSGLDSFTAGSILQVLQTLAQGGKTVICTIHQPRSDLFGQFGSVLLLSKGGHVAYDGQAKNMVQYFSDLGYPCPDLTNPADHVLDLVSVNLQMQWREDEDRERVDKLLSEWHKVEKQLFNAGLLRENSASFDPKTLMPRKPAPFQIAYFILLQRGIIALSRSPQVYIARLTQPVGIGVVLVLFFTPLRSSYIGIFNRFGLVQQLLSLYFVGVLNNMASYPFERDVFYREQDDGLYGVLPFFAVYTTLEIPFEVVSAMVFCIIVVLPPGFPRTADFFFAAFYLSFVVINTGESIGIVFNTLFRHTGFALNVVSVILSVGVFMAGLLSLQMPDFFKGLNYISPLKYACQSLLVMAFKDVPFHCTPDTGGYDAMGNCIFTNGTQVLEAYGFKNHVRVYLGVAAVCLVLYRLVSLVILKLVRLRVGLRSLKSRDNV

>CaROA1 1274 aa 6 CTGs

MKEGASVLSISSENQVGVKVRNLTVSVKSQQQKTTKHTEDQEAQYEQGTSKILNNLSFDIECGQLVAIMGGSGSGKTTLLNTLSQRTNINNKNLGFSGSVTYETSSSNKHIKHAYLLQTDIFLPGLTVWETLSTQADLRLPSHVTKQEKIELIEYILDVLELSHLKNTYVASFSSNASTLSGGEQRRVSLAIQMLSKPAILFLDEPTTGLDTSSSLKLVHVLKKLASPEYGITIILSIHQPRPEIGQLFDKICLLTRGGRLVYFGNLANAEMYFNNLNFLGRDSDDHSKHILEYIMDLSVKDTTSVEKEQQTVERINKLVQTWSNNHQFQQQQEENRSSKESHQFKKNLTLFSKPKTDKISFWQEVNVLTKRTFKLTFRDYKRLLVFNVGIVIIGVTVGWMFYRPKHDLAGIRSLTSTLYVAMEIMGFVPMYFEIERLWETDGVFFYREYSENQVSIPGFLISRRLGKLFLEDLPMSLLFSIITFFMWGLRLGDGSHFGIYFVVVFLIELCCMGTAMFSFAIAPSYPISALLINLIYQIQNSACGYFVNAATMPVYVKWTKYLAYFWYAFGALTNNQFSGWHGKCPYNDINDPRCQEFSGDYQIKILGFPVGWVGAPIGYLVLWTVGFFVLSGILFYFKQHDVSMAKTKKNTIGEGEEDHEALHQKKTEREQDYITDKHDLEININNIHLDVTTSNLFGQTKSTKTLLDNVTASFQANKVNVIMGPSGSGKTTLLNYLSNRLSRNSKFIASGSIRLNGIQKISRDQLSKISAYVTQHDSSLIEQLTVRETLYYQAKLRLPLDQHKFIPTIINKLIRQTGLVDCADTLIGSEYVKGISGGEKRRVSIAIQLLSRPKVLFLDEPTSGLDSSTAETILTLLGELAKENNTTIILTIHQPSEQLFYKFGSLLLLGRGGKVIYDGTSVGIVEYLESLGYNNPEGHNIADYILDLISRGMNEDKMQSERRVAELISYWQANSIKKLCSTATFSQEIIDLPQYYYQRLPIFITFPTIFRRQLLTSYRAKDVVINRAGQTIFLAIVHTLYFTPLRNTQEGISNRLGLVQEVLNLYFAGLINNITLYPFERNLFYQEYRDGIYGVTEFGLSYLINELPTEVIPCFFFAALIVFACGLPRTPQMFFAMFGTGFVSINCGESLGIFVNSIFTHMGVATNVLSTFVSLAIFMGGTMSLHMPGFFKGINFISPMKYAVAICANLGFKNQSFKCNSEAADCLLTTGEDVLSYYNMKQNLGPMVGGLIGCLVIYRVVAILSIYVRVKWF

>ScYOL075C [NP_014567.2](https://www.ncbi.nlm.nih.gov/protein/NP_014567.2?report=genbank&log$=protalign&blast_rank=1&RID=DDV8B4C1014) 1294 aa

MSQQENGDVATELIENRLSFSRIPRISLHVRDLSIVASKTNTTLVNTFSMDLPSGSVMAVMGGSGSGKTTLLNVLASKISGGLTHNGSIRYVLEDTGSEPNETEPKRAHLDGQDHPIQKHVIMAYLPQQDVLSPRLTCRETLKFAADLKLNSSERTKKLMVEQLIEELGLKDCADTLVGDNSHRGLSGGEKRRLSIGTQMISNPSIMFLDEPTTGLDAYSAFLVIKTLKKLAKEDGRTFIMSIHQPRSDILFLLDQVCILSKGNVVYCDKMDNTIPYFESIGYHVPQLVNPADYFIDLSSVDSRSDKEEAATQSRLNSLIDHWHDYERTHLQLQAESYISNATEIQIQNMTTRLPFWKQVTVLTRRNFKLNFSDYVTLISTFAEPLIIGTVCGWIYYKPDKSSIGGLRTTTACLYASTILQCYLYLLFDTYRLCEQDIALYDRERAEGSVTPLAFIVARKISLFLSDDFAMTMIFVSITYFMFGLEADARKFFYQFAVVFLCQLSCSGLSMLSVAVSRDFSKASLVGNMTFTVLSMGCGFFVNAKVMPVYVRWIKYIAFTWYSFGTLMSSTFTNSYCTTDNLDECLGNQILEVYGFPRNWITVPAVVLLCWSVGYFVVGAIILYLHKIDITLQNEVKSKQKKIKKKSPTGMKPEIQLLDDVYHQKDLEAEKGKNIHITIKLEDIDLRVIFSAPFSNWKEGNFHHETKEILQSVNAIFKPGMINAIMGPSGSGKSSLLNLISGRLKSSVFAKFDTSGSIMFNDIQVSELMFKNVCSYVSQDDDHLLAALTVKETLKYAAALRLHHLTEAERMERTDNLIRSLGLKHCENNIIGNEFVKGISGGEKRRVTMGVQLLNDPPILLLDEPTSGLDSFTSATILEILEKLCREQGKTIIITIHQPRSELFKRFGNVLLLAKSGRTAFNGSPDEMIAYFTELGYNCPSFTNVADFFLDLISVNTQNEQNEISSRARVEKILSAWKANMDNESLSPTPISEKQQYSQESFFTEYSEFVRKPANLVLAYIVNVKRQFTTTRRSFDSLMARIAQIPGLGVIFALFFAPVKHNYTSISNRLGLAQESTALYFVGMLGNLACYPTERDYFYEEYNDNVYGIAPFFLAYMTLELPLSALASVLYAVFTVLACGLPRTAGNFFATVYCSFIVTCCGEALGIMTNTFFERPGFVVNCISIILSIGTQMSGLMSLGMSRVLKGFNYLNPVGYTSMIIINFAFPGNLKLTCEDGGKNSDGTCEFANGHDVLVSYGLVRNTQKYLGIIVCVAIIYRLIAFFILKAKLEWIKW

>AsflB8NPU1 [XP_002382356.1](https://www.ncbi.nlm.nih.gov/protein/XP_002382356.1?report=genbank&log$=protalign&blast_rank=1&RID=DDVKUC1S014) 1385 aa

MESSGEIKEYDASEDSHQDAEIDRLVDDFLQNQTTSVSERLGDIIFKDLSVIGAGAGHQRMHDVPKTLQRLSKLANLATWSSRKPPPCRAILQRLTGTIRQGEMLMVVGRPGSGCTTVLKALANIREEYLAMEGDVWYGSMDAGTAKQARANQVAFVGEDDIHFPTLSVSTTLKFALNTRRSTSDPDRAQHLQQDLQTVLELMGLAQAAHVRIGSDHIRGVSGGQRRRVSLAEALCTRASLFCFDNPTRGLDSSTAIRFLTTMRKYTTRSQCMTAMSLYQASDLAVAMFDKVLVLNDGHVAYYGPATSAKAYFESLGFYCSPKISVSDFLASMSGTPEGRTPREALDRPVPIHPADFETRFRESSLYQQTVSDAATPPQSKTVGKPKASGYALPLYRQVYECTVRHYQIFLTDRAAWIAEAAGTIVQALLLGTLFRNQRDVTQGLYTRGSALFFCVLIMGLQASAEFGNTFVQRPILLKQKSLRFYRPGAYALGQILADIPWKFIFIMYSLPIYWMINFQRTAGHFFTWLVCLYMGLMALSVMFRAIAVFTNSITRAILPVGLLLNVFIIYTGFYITPPGMKVWLFWIRYLDPMYYIFESVALNEIGTSSYQCSSGDIVPRGSAYNETSYQACAVSGSVAGELSLSGRLYLMAEYGFKNTHLWRNVGINAGFFVFFSVVVMIGMERFRNAAEHMSTIFYRRLPSWVSASASRSADIEEPPIVAETKDSKPSSNHDVKTIGRLETTQSVFAWQELSLQLGDDKRLLHEVSGWLQPGKMTALMGMSGAGKTTLLDTLAQRIQIGRLSGGLYLNGQTLPASMGRRTGFVHQNDIHLASSTVREALQLSACLRRPATVSWDEKMDHVEMLIQLLEMEDIAEAIIGVPGAGLNLEQRKRVSIGVELAAKPDIVLFLDEPTSGLDGNSALSIVQLMRRLSDAGQTILCTIHQPSAQMIEQFDNLLLLVPGGKTVYFGPLGSRCQKILDYFARYTRRCEETENPADYLLAVSAEPDKDWFQVCCSHPFLLPLTWRQSPEYGSTQEQLQKMLQVQEIKDSSSSESDRTYAASYLNQLRVVTQRAFTNYWRDSDYVLGKVQLNIWMGLMNGLTFLQLSNDLTGARGRMFSIFVGVITGPVLSLQIEPRFILLRDQFLARENESRVYHWSIFTISALLVEIPFTLLGGLIYWLLWYYMVGYLTISTRAGYAFLMYELYSLFVASLAQLTASLFPTVLAAQVATGFIWLVVNTFNGPLSPPPLTPRGWRWFYNISPLFYFIEGIGTNAMHALQITCRDSELTTFQTPAGETCASYTAEFFGLANSTGYLVDPNATGLCEYCAYADGDEYVKQYDMSYSQRGNNVGIFIGFILFNYTMAVLATYLIFIFKWRKRRSN

>AsclPeaB1 [XP_001273095.1](https://www.ncbi.nlm.nih.gov/protein/XP_001273095.1?report=genbank&log$=protalign&blast_rank=1&RID=DDVGFACC014) 1397 aa

MDMLRNRPVKGSLDHDPQLSADGTNTPAASDGSDQRSDVIEKTAQLSEPIADSIRRFFEIRKLDGPDGTGVVFENISVEGSGTGAQAAPTISSAARSAFGVLSPLQHRLAGQFSRPILSGFSGTIDAGEMLLVIGKPGSGCTTFLKTLSYMWDEYKDVHGDLTIGGHPIQESMVKRPQDIVFCAESDDHFPTLTVAETLRFAIRARCGPEASATEVDMMVAQLAKLVGLSQVMNTKVGDAYIRGVSGGERRRVSLAEALATCARLICLDNPTHGLDSSTALEFIETMREWTSQSRCVTAMSVYQASDAIMPYFDKVLVINSGRQVFYGRIGDAKAYFERLGFECLPTTTLSDFLNSMSADPEVRRVQDGKQHLVPRTSEEFEAVFHASTFYQDLQRSLETAKVEARTNPRPLVKARAFSLPLHHQIWYCAYRQFRIVTSDYSLWAVEPATIIVQSLVLGTLFRDQKRATQSLFIFASALFYSVLVPALQSMAEFGNGFAQRPLILKQKRYRICRPIAYALGLVTTDVVWKIAAICYNIPLYFLTGFQRTAGNFFTWFCIVYLEHLALSMFFRSVAIFSPNMHRAVLPVGIFFNMYVLYTGLYIPAPQMQVWLGWLRYLNPLYYAFESVMVNEFRDLSYQCSPSDLVPSGLGYTDMANQVCAVLGSRSGEESLSGMSYLEAQYGFGRSHLWRNVGINAAFFVFFALCSGIGMERLKTPAGRLATVFYKGRPSIRNSQADSESGAVHDDVPPDVSRQLSGDQHHLNANSERDKNHTLAWTGLCLDIETKDGTRRLLDNLNGWVKSGQLKALMGVSGAGKTTLLNTLAGRSSIGTLTGTLALNGQLLPKFFRSRMGYVQQQDIHLPTQSVREALQMTARLRRDESIPLEEKNAYVEKVIEWLDMEDIAEALVGVPGAGLNLEQRKRVSIGVEMASKPEILFLDEPTSGLDGQSAFSIVRLLRRLADSGQAIVCTIHQPAAELVEQFDELYLLSRGGKLVYDGPLGTHCDKAIEYFEQHSRACGQGENPAEYFLDAIGAGSRKEVQADWVGLWQQSQQSKDRERAEKALVPAEGQAPLAPARRSLYAVPFHVQLWVVVQRTWLYYWREPDYAMSKLWMSVGNALLNSLTYLQSPNTQRGAYNRVFSAFMSLIVGPPLGLQVQPRFVTLRDIFVHREREGFTYHWLAFVFAGIIVELPYTFLTSLVYWLLWYFPVGYFRTAPRAGYSFLMYELFAVFATSLAQMCASLMPNIEAAFAANGFFFMFCNTFAGTLSPKPVTPSGWRWYYKVSPLFYLGEGVTVDVLQDLPLRCAESEVSIFQPPNGTTCGQYAANFLQQATGFLLNTDSLSDCQYCRYRDGQSYYQQYGYDFANRYPNIGIFIGFIAFNFTMVLVMTYLTKIRR

>PechB6GX92 [XP_002556924.1](https://www.ncbi.nlm.nih.gov/protein/XP_002556924.1?report=genbank&log$=protalign&blast_rank=1&RID=DDVRSHTD016) 1369 aa

MTATKEQSIATATRTLAGDLVADFVTACQSSVRSKDNSIVWDHLEMEGGGQGHALAPSVGSMVERYARRATSVFGHRSEPSRKLLHYFRGHIKAGEMLMVIGRPGSGCTTFLKSLCHMHAEYKSTTGTLLYGGIQANFEEPAAPVETTFCAEEDIHFPSLTVEETLRFAVNSRFSNVISSAEAHKTVVNLARLFGIDHVLATKVGNEQIRGVSGGERRRVSLAEALVTCPDLICYDNPTAGLDSSTALEFVQMLREYANQSHCTIAMSLYQGSDDMVPLFDKVAVINPGHCIYYGNVVAAKSYFEDLGFYCPPTMSITDFLNSMSAEPEARQSRQAADSWSIPQTPEEFVTAFWKSEKGVRLGTQIEEAKNSASAAEKALGRRKESRQTYSIPILAQILLCAYRQYRIFITDYNAWIVEAACMVVQSIILGTVFRNLPHETSSLYQLGSVVFYAILVPGLQSMSEFGNTFAQRPLLLKHKRYRLYHPMSYGYGQILSDVVWKVVVIAYNIPMYFLAGLHRTAGHFFIFFLVAYISHLSLSMFFRFIAVLSPTVERAGLPVGIFLTTLVIYTGWYIPPPQMQVWLKWFRFLNPMYYAFEALMINEVGTTSYECTSSDLVPRGQTYTDVAYQACAIAGSEPGRTVVEGASYLRVYYDFDNSHLWRNVGINAGFFIFFAVLNVAHIVPSSTSSTENDLEVQGFARPTRKLSSPEEVRSRGGHSFAWKDLHLTLRKDGQERTLLQHIDGVSMTYLQILFSSPLYSDMRLNLGCIESGTLTALMGVSGAGKTTLLNVLAERMDIGKLTGTLYLDGSPLPKSFRWRMGYVQQQDIHLPSQTVREALQMTAHLRRPPSLSTDEKNAYVEEVLDMLGMQDISDALIGVPGAGLNLEQRKRVSMGIELAAKPDILLLDEPTSGLDGQSAISLVQLLKKLSRSGQTILCTIHQPAAAVIEAFDNLILLAKGGRVTYQGPLGEHSSTALKYFSQHVEACDPKRNPAEYLLDVVGAGSRSNVTADWAQIWSESSECRVQDAKLHDLKNEPTAEQSRPQLYATPLSHQFAIVLRRTWLWYWREPEYFSAKLWMNVANGLLNGLTFLNIPNSQQGAFDRVYTIFLSFLMGPPLGLSMEPRFTTFRDIFVYRERASRSYHWIVFVMSSIVIELPFTLITALIYWLLWYFPAGLQTDPTHAGYALLCYWLFSIFTVSLGYLIAAWMPNLNASLMANGFFFMFVNTFAGTLTAREKTPSGWSWYFNVSPLYYLAEGLTTNALYGHELGCTPSEATVFHAPANDTCISYAGSFLQSATGYLVNPDATGACDYCRYSIGQEYYQQFGYDNNRKYRDIGIFIGFIAFNFTAVIVGTYVTKIHKWKRKTE

>AfuQ4WN09

MDAERAVEHRKELDASPQGRSSHDIANSAASPEASGPDTEESSSIDDEPSTAFEDVSSKSTRDGQYGDEHINRILSRRHTSRSEEGVEDMAQIAKLMSHMFGKERKSVSDEEKTRHAGVIWRDLTVKGVGLGAALQPTNSDIFLAVPRRIKDLLTRGRKGIGAGHHPLRTILDDFTGCVKPGEMLLVLGRPGSGCSTFLKVIGNQRAGYKSIKGDVRYGGADADLMADKYRSEGTVAIIHTASILSLIESAVSYNPEDDLHYATLTVRDTLLFALKTRTPGKDSRIPGESRKDYQHTFLSAIAKLFWIEHALGTKVGNELIRGISGGEKKRVSIAEAMITKASTQCWDNSTKGLDASTALEYVQSLRTLTDMANVSTLVALYQASENLYNLFDKVMLIEEGKCAYYGSAKEAKAYFERLGFECPPRWTTPDFLTSVSDPHARRIQRGWDDRVPRSGEDFRRVYRNSDTYRAALQEISQFEKELETQEHERAQARQEMPKKNYTIPFYDQVIVLTRRQFLIMYGDKQTLVGKWCILVFQALIIGSLFYNLPPTSGGVFTRGGVMFFILLFNALLAMAELTASFESRPIMLKHKSFSFYRPSAYALAQVVVDVPLVFVQVTLFELIVYFMSNLSRTPSQFFIQFLFIFILTMTMYSFFRALGAVSASLDVATRLTGVAIQALVVYTGRMKFFPNGTLSHGALGYLIPPWKMHPWFKWLIWINPVQYAFEAIMANEFYNLDIQCVRPNIVPDGPNAQPGHQSCAVQGSTPNQLVVQGSSYIKTAFTYSRSHLWRNFGIIIAWFIFFVALTMLGTELQQPNKGGSSVTTFKRNEAPKNVEEAVKNKELPEDVESGQKENAVNADSEKTQPGETGDEVKDIAQSTSIFTWQDVNYTIPYEGGQRKLLQDVHGYVKPRRLTALMGASGAGKTTLLNTLAQRINFGVVTGTFLVDGKPLPKSFQRATGFAEQMDIHEPTATVRESLRFSALLRQPKEVPIQEKYDYCEKIIDLLEMRPIAGATVGSGGVGLNPEQRKRLTIAVELASKPELLLFLDEPTSGLDSLAAFNIVRFLRRLADAGQAILCTIHQPSAVLFEEFDDLLLLQSGGRVVYNGELGHDSNALIEYFESNGAKKCPPHANPAEYMLEVIGAGNPDYKGKDWGDVWAQSPQCKQLAEEIDKIISSRRNREIRKNKDEHREYAMPIWTQIVTVTKRAFVAYWRSPQYTLGKFLLHIFTGLFNTFTFWHLGNSYIDMQSRLFSIFMTLTISPPLIQQLQPRFLHFRNLYESREANSKIYSWTAMVTSAILPELPYSVVAGSIYFNCWYWGVWFPRDSFSSGYTWMLLMVFELYYVSFGQFIAAFSPNELFASLLVPCFFTFVVAFCGVVVPYVALPHFWQSWMYWLTPFHYLLEGFLGVLTHNIPVRCVSREVTQVSPPSGQTCQTYAGAFARQAGGYVEDAAGGLCSYCPYSIGDAFAASFNVFYSHKWRAYGIFWAFTVFNFAAVYFFSWLYLHGVGDLKRWISARKTRKIVK

>MagrQ2KEJ1 [XP_016846008.1](https://www.ncbi.nlm.nih.gov/protein/XP_016846008.1?report=genbank&log$=protalign&blast_rank=1&RID=DDWH61HP014) 1559 aa

MDDRPTPKDTDIPGGFPESPPGPTPLTERNFPLVTSQLRASRSHVDAPTEQIRRPGPAIGGDIGSTSSSLTEKEGLNNETASQDDGSEKRFAPLRTDSQAVRATGSDARPGFERKATSYTEDDIFRALSRRRTGGTHASAPAESDEDEGQEIERLVSRMFGHERQRQSAEEKTRRSGVIFRDLTVKGVGLGATLQPTVGDIFLALPRKVGHLFTKGPRAAFAKPPVRELISHFDGCVRPGELLLVLGRPGSGCSTFLKTFCNQRAGFESVLGDVSYGGVDAKTMARDYRGDIIYNPEEDLNYATLSVKRTLHFALETRAPGKESRLEGETRQDYIREFMRVITKLFWIEHTLDTKVGNEYVRGVSGGERKRVSIAEAMIARASVQGWDNSSKGLDASTAVEYLRSIRAMTNMANTSTAVSLYQAGESLYELADKVLLIDAGKCLYFGPSEQAKQYFIDLGFHCPERWTTADFLISVTDPHERHVRQGWEDRFPRTPEQFAEAYRRSNIYRANLEDMSRFEAEQQQQVEARAAIEAGKPKRERTKNYEIPFHKQVIACTKRQFLVMIGDKASLLGKWGGLVFQGLIIGSLFFNLPETASGAFPRGGVLFLLLLFNALLALAEQTAAFESKPILLKHKSFSFYRPSAYAIAQTVVDVPLVFIQVVLFTVIIYFMSHLARTASQYFIANLILWLVTMTTYSFFRAISAWCGTLDVATRFTGLAVQILVVYTGYLIPPTSMPVWFGWLRWINWLQYGFECLMSNEFYRQELTCNGPFLVPQGPQAEPQYQGCTLAGSTPGDSTVSGANYIAESFSYTRAHLWRNFGFLWAFFIFFVLLTALGMERMKPNKGGGAITVFKRGQVPKQLESTIETGGKGKGGNEKDEEVGTTGSDSQAPVSPREGSTEEDDKRSNQVAENETIFTFRDVNYEISSKGGKRKLLSDVQGYVRPGKLTALMGASGAGKTTLLNTLAQRIQTGTVTGEFLVDGRPLPKSFQRATGFAEQMDIHEPTATVREALQFSALLRQPREVPKQEKLDYCETIIDLLEMRSIAGATIGNVGEGLNTEQRKRLTIGVELASKPELLMFLDEPTSGLDSGAAFNIVRFLRKLADAGQAVLCTIHQPSAILFENFDELLLLKAGGRVVYHGPLGHDSQDLLGYLEGNGAHKCPPNANPAEYMLDAIGAGDPDYKGQDWGDVWQNSKEREARTREIDDMISQRQQAEQTQSLRDEREYAMPLSAQMSAVVRRSFVSYWRNPGYLVGKFMLHILTGLFNCFTFFRIGFASIDYQNRLFSVFMTLTICPPLIQQLQPVFIDSRQIFQWRENKAKIYSWSAWVTGAVLAEIPVAVLAGAVYFNCWWWGIFGWRDIMPASSSAFAFLMVVLFELYYVSFGQAVAAFSPNKLLASLLVPLFFTFIISFCGVVVPPAQIPTFWREWMYWLSPFHYLLEALLGVAVHAQPLECSADELARFEPPPGSSCEEYAGPYVAQAGGRLRTGQGGMCELCQYADGDQFAASFSVYYYNRWRDVGIFCGFIVFNYAVVYLATWLKFKGRNPLKMFGKRKSG

>Mygratr1 [XP_003857636.1](https://www.ncbi.nlm.nih.gov/protein/XP_003857636.1?report=genbank&log$=protalign&blast_rank=1&RID=DDW4059B016) pleiotropic and azole drug resistance 1562 aa

MWGYSVDERKLQREDTNGPPTNQWHNAQRTGGTHPTEEVEGEGEGTWGENDVGGFTTRQAMEDYEALRKDLTQLSKTRSRDTQHSLKRTTTGQTAKSGRKSLTSRQATHTSEAAEQDVEAGPQEEVEESKKDDDDEDDFELDRFMREGHFEKRSDGTSDKRVGVVYKDLTVKGIGSTTSFVRTLPDAIIGTFGPDLFKIICRFVPALAKRTGETRTLLNGFTGCVRDGEMMLVLGRPGSGCSTFLKAISNNRETYAEVTGDVSYGGIPADKQKKMYRGEVVYNQEDDVHFATLNVWQTFIFALMNKTKKKETGNIPVIAEALMKMFGIPHTKYTLVGDDFVRGVSGGERKRVSIAETLASKSTVVCWDNSTRGLDASTALDYARSLRVMTDVSNRTTLVTLYQAGEGIYEVMDKVLVIDEGREIYSGPAKEARQYFIDLGYEAPERQTTADFLTAVTDPVERKFRKGYEHKAPKGPEALEKAFRESPNYQKVLEDITDYENYLKETDYNDAREFEDAVQDGKSKRVSNKSSYTVSFQRQVLACVKREAWLLWGDKTTLWTKLFIIISNGLIVGSLFYGESFDTSGAFTRGGALFFSILFLGWLQLTELMKAVSGRAVVKRHEDYAFYRPSAVTIARVVMDLPVILVQVLIFGIIMFFMTNMTISASQFFIYMLFVYITTILLTALYRMFASLSPEIDTAVRFSGIALNLLVIYTGYVIPRPQLLTKYIWFGWIYWINPLSYSFEAVITNEFAGRTMACAPSQLVPQGPGIDPAYQGCALAGADVNAQSVDGSAYLATQFNYSRSNLWRNFGVVIAFIVLYILVTVIATETVSFAGGGGGALIFKKSKKAKKQVKHAKHADEEKGGIAEDSSSSSKKNASLGDAPNEDKEDEALDKLTKSESIFTWKDVEYTVPYMGGERKLLNKVNGYAKPGVMVALMGASGAGKTTLLNTLAQRQSMGVVSGEMFVDGRPLGREFQRNTGFCLQGDLHDGTATIREALEFSAILRQDASVSREEKIAYVDTVIDLLELNDMQDAIISSLGVEQRKRLTIGVELAAKPSLLLFLDEPTSGLDSQSAYSIVRFLKKLASAGQAIVCTIHQPSSVLIQQFDMILALNPGGNTFYFGPVGENGKDVTKYFSDRGVDCPPHKNVAEFILETAAKPHKRKDGKKIDWNQEWVESQQAKDVLEEIDGLKQTRSHVSTSQKNKDDEKEFAASTMLQCTELLRRTFRQYWRDPSYLYGKFFVSVIVGIFNGFTFWQLGNTQQDMQNRMFTAFLIITIPPTIVNAVVPKFYTNMALWQAREYPSRIYGYFAFVTAQVVAEIPPAIIGAVLYWVLWYWPTGLPTDSSTSGYVFFMTLLFFLFQASWGQWITAFSPSFTVISNVLPFFFVMFSLFNGVVRPYASLPVFWRYWMYYVNPSTWWIGGVLAATLDGIPVQCAETETAHFDAPPGQTCASYAGAFAQSAGGYLLNPQDNTNCMYCPLSTGNQYLAQLNINASDKWRDLGIFVVFVFSNWFLVYFFIYTVRVKGWTFGFGPLFGALGKGVELIKKPFKKGEKKEQSEE

>PechB6HGI7 [XP_002563711.1](https://www.ncbi.nlm.nih.gov/protein/XP_002563711.1?report=genbank&log$=protalign&blast_rank=1&RID=DDW1CRMC016) 1556 aa

MEGHSEQSHTVSDSQAHAQSSRQQPSSTGETYHHDASSGPTTEQIDQTTEAPIDATLTDSSTASSSVSDEDGRWGEQKAGKAVSRSGAMEDMEEMRRELTRLSLSRTRSATKSIRRRKSQASRRDEEKAQDEEETEDEAADSFDLGEFLTGGHLERRTTAGEPAKKVGVVFKNLTVQGVETGASFVRTLPQAVVGTFGPDLYNIVCRFVPQLRFGKHPPVRDLIHDFNGAVREGEMMLVLGRPGAGCSTFLKAIANDRGAFAGVNGEVSYGGLSAEDQNKHFRGEVNYNPEDDQHFPSLTVWQTLKFSLINKTRKHDRESIPIIIDALLKMFGITHTRNTLVGNEYVRGVSGGERKRVSIAETLATKSSVVCWDNSTRGLDASTALDYAKSLRIMTDVSKRTTFVTLYQAGESIYELMDKVMVIDEGRMLYQGPANEARQYFVDLGFYCPPQSTTADFLTSLCDPNAREFQPGREASTPKTAEELENAFKNSLAHKRILEDVSSYEKRLQDTQQEDTRRFQSTVAQSKSKSVSKKSPYTVSFVRQVMACVQREFWLLWGDRTSLYTKYFIVISNALIVSSLFYGESLDTSGAFSRGGALFFSILFLGWMQLTELMPAVTGRGIVARHKDYAFYRPSAVSIARVIVDFPAILAMVIPFTIVVYFMSGLDVTASKFFIYFLFVYTTTFCITSLYRMFAALSPTIDDAVRFSGIALNLLILFVGYVIPKQNLISDSIWFGWLFYVNPIAYSYEAVLTNEFSDRVMKCNPSQLVPQGPGVDPRYQGCALTGSTLGESSITGSQYLTANFQFTRSHLWRNFGVVIAFTVLYLLVTVIAAEVLSFVGGGGGALVFKKSKRTKKVAAPATNDEEKVANSNDNAALARGQASSDNGASFNRLSSSERCFTWQNVEYTVPYGNGTRKLLNGVNGYAKPGVMIALMGASGAGKTTLLNTLAQRQKMGVVTGDMLVDGHKLGPDFQRGTGFCEQMDLHDNTATIREAFEFSAILRQPRDVSRQEKLDYVDRIIDLLELEDIQDAIIGCLTVEQKKRVTIGVELAAKPSLLLFLDEPTSGLDSQAAFSIVRFLRKLSQAGQAIVCTIHQPSSMIIQQFDMILALNPGGNTFYFGPVGKDGSAVIKYFGDRGVVCPPSKNVAEFILETAAKPNHRNGKLLDWNEEWRNSDQNREMLAEIENIRTERSKVPIEETGSAQYEFAASTLTQTTQLTKRLFTNYWRDPSYYYGKLFVSVIIGIFNGFTFYKLGNDVASMQNRMFSVFLIILIPPIVLNSIVPKFYINRALWEAREYPSRIYGWVAFCTANVVCEIPAAIISGLIYWLLWYYPVGFPTDSSNAGYVFLMSMLFFFFQASWGQWICAFAPSFTVISNVLPFFFVMVNLFNGIIRPYADYPVFWKYWMYYVNPVTWWLRGVLSAVLPDVQIECAPLEATHFNPPPGQTCDAYAGGFVETAKVGYLVNPQATADCQYCPYTDGVQYMANLNVHLEDKWRCFGIFLAFVIINWALVYFFIYTVRVRGWSFGIGSLFGVAGLMVDRVKGLFKGKKSDEA

>Cne04966

MAEVPSINDSEWNLASQLRADQELLKSRGLEPYKSLPLAWEHLSVRGVGGLDNIEYGSSMVTILAPWLRRKYRKKAALLAAARSDLPGAEKGDGDVMVWRPGMPTPKKGEPGLRKGERYLLKDFSGVVKPGEMMLVVGRPGSGCSTFLKILAGHREGYAGVEGMVKYGALQPGKDFSPYKSEVIFNSEEDLHDPNLLVGHTMDFALQMCTPSRDSRLPEEPAGIGMSRKKYQDRTKWELLKTLGLTHTHDTKVGDQYVRGVSGGEKKRVSIAEVLATKASVQMWDNATRGLDADTALRYAKTLRTLADIQRNTTVVSLYQAGNGIYDLFDKVTVIAEGRVIYYGPRAEARSYFEDLGFVHPDGGNTADFLTAVTATNERKIREGFASPIPTTPAEFSTLYEKSDIARRMREELDAHLADPALDEQTEKFRGSVAKQKGRWASEDRPEKVDFMTQVHGAIIRDYRQRWGDKWTFWMRPATLLFQALIAGSMFYDMPVSTAGLFLRGGTLFLSLFFPSMISLGETTAVFSGRSVLSKHKGFSMYRPSAVLLAQTIGDMPLYFVMIVMFTLIIYFMTGLKVDAGLYFMYLLFVYFTTLCTTALFRSIGYAFSTFNNASKASGFALLVLSMYAGYIIYTPQMHPWFSWIRWLNPFYYSLEALTASEIYGLELACVSPQLAPYGGDYAQYNQGCAITGAEPNSVTVDGTLWAESALRFYKSHVWRNFGILMGFWVFFLGVCALMIEMIPAAGSTKSILLYKPGGGGKYIRNAQMNGVSPRDEEDGPNDSQLNEKSQGTSDNTAAEVHAVNSVLTWKNLCYTVNVNGKPRQLLNNIFGYCKAGTLTALMGSSGAGKTTLMDVLAARKTDGDIRGEVLMNGKQLPISFQRTTGYCEQVDVHLPQATVREALEFSALLRQPRTLSDKEKLAYVDVIIDLLELHDIEDALIGTPEAGLGVEQRKRLTIGVELVSKPTLLFLDEPTSGLDGQNSYLIVSFLRKLAATGQAVLCTIHQPSAALFAQFDQLLLLKGGGNTVYFGAVSELTSYFEKQGVTIPKDVNPAERMIDIVSGDLSKGRDWAQVWLESDECKERARELEELKEAGANNITIVEGGEYEFASTNMTQLKLVTKRASIQLWRDTEYVMNKVALHVMAALFNGFSFWKIGEAYADIQNRIFTIFLFVFVAPGVIAQTQPKFLHNRDIFEAREKKAKLYSWHAFCFAEIVAEIPYLLVCALLYFASWYPTIGFSFKPGVAGPIYLQMTLYEFLYTGIGQFVAAYAPHEVFASLVNPLLIGVLVIFCGVLVPYDQITAFWRYWMYYLDPFQYLLGGLISPALWDVEVKCKSDEYAIFDPPEGMTCENYMSAFLSEAPGYLNNPNATSDCEYCVISKGSDYLNALNLGRKVDGWRDIALTFLFVLTSYGMVFLLLKLRSKRSKKAQ

>PechB6HHZ7 [XP_002567612.1](https://www.ncbi.nlm.nih.gov/protein/XP_002567612.1?report=genbank&log$=protalign&blast_rank=1&RID=DDXCH1A5016) 1415 aa

MALSTSSTDSNIEDISNLEKLPDETREIEAQYPTGDVFPGTQATWHMADELQALKERDEQNGEKARKLGVTWQNLTVKGVSSDATFNENVLSQFNLFGNHGSKSPMKTILHNSHGCVKPGEMLLVLGRPGSGCTTLLNMLSNNRRGYAEVSGDIAFGNMSAEEAKQYRGQIIMNSEEEIFFPTLTVGETIDFAARMKVPSQLPPGIKSAEEYAELNKKFLLRSVGISHTESTKVGDAFTRGVSGGERKRVSILECLTTRASVFCWDNPTRGLDASTALEWTKAMRTMTDVFGLTTIVTLYQAGNGIYENFDKVLVLDEGKQIFYGPQRNAVPFMENLGFRRDSGSNRADFLTGVTVPTERIIAPGYESTFPRTSDAIRSAYESSSSKSEIQAECSYAQSKEAAENTAIFKEMVAREKHHGVREKSPVTTDFLSQVKASVTRQYQIMWGDKATLAMKQGATVIQALLGGSLFYNAPDNSIGLFLKGGALFFSILYNALIALSEVTDSFTGRPILAKHRSFALYHPAAICISQIVADFPILLFQVSHFGLVLYFMVGLNRTAEAFFTYWITNFMTAMSMTALFRLIGAAFPTFDAATKVSGLTIVSCFVYTGYMIIKPEMHPWFVWLFWINPMAYGFEALLGNEFHSSIIPCVGPNLIPNGPGYTNGEGGQSCAGVGGASPGATSVTGREYLASMSFSHSHVWRNFGIICAWWVLFVALTIFFTSRWKLPGEGARSLLVPREQQYKSKHLLLGDEESQSMKTLPNSEANTSQETIGKELNGNRSIFTWKNLTYTVKTSSGDRVLLDNVQGYVKPGMLGALMGSSGAGKTTLLDVLAQRKTDGTIHGSVLVDGRPIPISFQRSAGYVEQLDVHESLATVREALEFSALLRQPRDTPIDEKLRYVDTIIDLLELRDLEFTLVGRPGAGLSVEQRKRLTIAVELVAKPSILIFLDEPTSGLDGQAAFNIVRFLRKLAEAGQAVLVTIHQPSAQLFAQFNTLLLLAKGGETVYFGDIGDNASTVKAYFARHGAPCPPEANPAEHMIDVVSGAASENADWNKIWLESPEHDQLTTELDAMATEAAARPSGTVDDGHEFAASMWTQVKLVTHRMNVSLFRNTEYIDNKFALHISLALLNGFSFWMIGDRLTDLQKNLFTVFNFIFVAPGVISQLQPLFIDRRDLYETREKKSKMYHWAPFVAGLIISEIPYLIVCALLYYFCWYFTCGLPTAPGNAGSVFFVVVMYECLYTGIGQMIAAYAPNAVFASLVNPLVITTLVSFCGVMVPYSQIEPFWKYWMYYIDPFNYLMSSLLVFTTWSKPVTCTPDEVALFNPPVNQTCGEYLATYQQGMGVGTNLLNPSANADCQVCQYTTGGDYLKSLNLAEEYFGWRNAGLVALFVLGIYGLVFLMMKLRTKATKKAEN

>AfuQ4WFQ9 [XP_748461.2](https://www.ncbi.nlm.nih.gov/protein/XP_748461.2?report=genbank&log$=protalign&blast_rank=1&RID=DDXAGCGW016) 1424 aa

MEDQGHLPSEPRALFDRRDDTDSTNTALDETDLSRTPLQDTSHTPHAEDWSLMPDLKKQHDRNVASGFRRRELGVTWKNLSVDVVSADAAINENVLSQFNIPQHIRESRNKAPLRTILHESHGCVKPGEMLLVLGRPGSGCTTLLRMLSNHRLGYKAIRGDVRFGSLTPEEASKYRGQIVMNTEEELFFPTLTVAQTLDFATRLKVPFNLPDGVTSPEAFRQETREFLLKSMGISHTSDTKVGNEYVRGVSGGERKRVSIIECLATRGSVFCWDNSTRGLDASTALEWAKAVRAMTDVFGLSSIVTLYQAGNGIYDLFDKVLVLDEGKQIYYGPMSQARPFMEEQGFVCREGSNVADFLTGVTVPTERKIRPGYENRFPRNADELLAAYEKSPIRAQMAIEYDYPDTESTRERTEEFKLGVLDEKAKRLSKNSPFTVDFLQQVKACIIRQYQIIWTDKATFAIKQISTVIQALVAGSLFYNAPDNSGGLFIKSGALFFSLLYNSLLAMSEVTDSFSGRPVLIKHKYFAFFHPAAFCIAQIAADIPVLLFQISMFAVVVYFMVGLTTSAGAFFSYWIIIFVATMVMTALFRAIGALFSTFDGASKVSGFLISALIMYCGYLEPYHAMHPWFIWIYWINPLAYAFDALLSIEFHNKIIPCVGNNLVPFGPGYDDTTFQSCAGVGGAVRGMTYVTGDQYLASLTYSYSHVWRNFGILWAWWALFVAVTIIATSRWKSAAEAGNSLLIPRETVAKHHAVVRKDEEAQLNEKAGHKGTGTDSEAQSNVDQHLVRNTSVFTWKNLTYTVKTPSGDRVLLDNVYGWVKPGMLGALMGSSGAGKTTLLDVLAQRKTDGTIRGSIMVDGRPLPVSFQRSAGYCEQLDVHEPFATVREALEFSALLRQPRHIPREEKLKYVDVIIDLLELHDLEHTLIGRVGAGLSVEQRKRVTIGVELVSKPSILIFLDEPTSGLDGQSAFNTVRFLRKLADVGQAVLVTIHQPSAQLFAEFDTLLLLAKGGKMVYFGDIGDNAQTVKDYFARYGAPCPANVNPAEHMIDVVSGHLSQGRDWNQVWLESPEHSSASRELDSIISEAASKPPGTVDDGYEFAMPLWEQTKIVTQRMSTSLYRNCDYIMNKIALHIGSALFNGFSFWMIGDSVADMQLKLFTIFNFIFVAPGVINQLQPLFIERRDIYDAREKKSKMYSWVAFVTALIVSEFPYLCVCAVLYFVCWYYTVGFPSDSDKAGAIFFIMLCYEFLYTGIGQFIAAYAPNATFAALTNPLILGTLVSFCGVLVPYAQIQAFWRYWIYWLNPFNYLMGSMLVFSVFDTDVKCKEGEFAVFDTPNGTTCADYLSTYLQGVGSRANLVNPEATSGCRVCQYRYGSDYLYTINLKDYYYGWRDTAIVCIFVLSSYALVYALMKLRTKASKKAE

>PytrCDR3 [XP_001933058.1](https://www.ncbi.nlm.nih.gov/protein/XP_001933058.1?report=genbank&log$=protalign&blast_rank=1&RID=DDX2AADR016) 1420 aa

MDTQGSTPVATPSSSSEKMQHHDKGSMQSGKDGSVKTHVEWRLAEDVKDFDMNNQAGVRRLGVTWRDLSVEVVPSDERLQENIISQFNVPQLIKDARRKPALKPILESTSGCVRPGEMLLVLGRPGSGCSTLLKMLANKRNGYAKVNGDVHFGSLDAKQAEQYRGSIVINNEEELFYPTLTVGETMDFATRLNTPETIQDGRSQEEARNKFKGFLLNSMGISHTENTKVGDAYVRGVSGGERKRVSIIETLATRPSIACWDNSTRGLDASTALEYTRALRCLTDTMGMATIVTLYQAGNGIYDLFDKVLVLDEGKQIYYGPREEARPFMESLGFICGDGANVADYLTGVTVPSEREIKPYFEDKFPRTAAEIQQAYQQSKIKAAMDRELDYPVSSEAKTNTQAFCQAVDSEKSRRLPKSSPMTVSFPAQVKACVIRQYQILWNDKPTLLIKQATNIVQALITGSLFYNAPDNSAGLFLKSGALFLSLLFNALFTLSEVNDSFTGRPILAKQKNFAFFNPAAFCIAQVAADIPILLFQITSFTLILYWMTALKATAAAFFINWFVVYVVTLVMTAMMRTIGAGFPTFNEASKISGFAITATIVYMGYEIPKPAMHPWLVWMYWINPLAYGFESLMANEYEGTTIPCVYDNLIPNYLPQYQDPNSQACAGIGGARPGANKVSGEDYLASLSYSPSNIWRNVGILFAWWAFFVALTIFFTCRWDDTSASSTAYVPREKSKKVAKLRASRAQDEEAQLGEKLSSNNATLGASGETKTGLEKSLIRNTSIFTWRNLTYTVKTPTGDRTLLDNVHGYVKPGMLGALMGSSGAGKTTLLDVLAQRKTQGTIKGEVLVDGRPLPVSFQRSAGYCEQLDVHDAYSTVREALEFSALLRQGRDVSKEEKLAYVDTIIDLLELHDLENTLIGKVGAGLSVEQRKRVTIGVELVSKPSILIFLDEPTSGLDGQAAFNTVRFLRKLADIGQAVLVTIHQPSALLFAQFDTLLLLAKGGKTVYFGDIGDNAETIKEYFGRYDCPCPPGANPAEHMIDVVSGYDPAGRDWHQVWLDSPESAALNQHLDEIISDAASKEPGTKDDGHEFATTFWTQARLVTNRMNISFFRDLDYFNNKLILHIGVAFFIGLTFFQIGNSVAEQKYVLFSLFQYIFVAPGVIAQLQPIFLERRDIYEAREKKSKMYSWQSFVTALITSEMPYLLICGTLYFLIFYFIAGLPAEASKAGAVFFVFLVYQFIYTGFGQFVAAYAPNAVFASLVNPLLLSTLCCFCGVLVPYAQIQDFWRYWLYYLNPFNYLMGSLLIFTDFDWKIECRESEFALFDPPAANGNQTCAQYLEAWLSGPGSRTNLVNPDATSGCKVCQYTVGRDYLATVNLGERYLGWRDAGICVIFAFSGYALVFLFMKLRTKQSKKAEK
